# Iron Catalyzed Double Bond Isomerization: Evidence for an Fe^I^/Fe^III^ Catalytic Cycle

**DOI:** 10.1002/chem.202004980

**Published:** 2021-03-15

**Authors:** Callum R. Woof, Derek J. Durand, Natalie Fey, Emma Richards, Ruth L. Webster

**Affiliations:** ^1^ School of Chemistry University of Bath Claverton Down Bath BA2 7AY UK; ^2^ School of Chemistry University of Bristol Cantock's Close Bristol BS8 1TS UK; ^3^ School of Chemistry Cardiff University Main Building, Park Place Cardiff CF10 3AT UK

**Keywords:** homogeneous catalysis, iron, isomerization, reaction mechanisms, redox chemistry

## Abstract

Iron‐catalyzed isomerization of alkenes is reported using an iron(II) β‐diketiminate pre‐catalyst. The reaction proceeds with a catalytic amount of a hydride source, such as pinacol borane (HBpin) or ammonia borane (H_3_N⋅BH_3_). Reactivity with both allyl arenes and aliphatic alkenes has been studied. The catalytic mechanism was investigated by a variety of means, including deuteration studies, Density Functional Theory (DFT) and Electron Paramagnetic Resonance (EPR) spectroscopy. The data obtained support a pre‐catalyst activation step that gives access to an η^2^‐coordinated alkene Fe^I^ complex, followed by oxidative addition of the alkene to give an Fe^III^ intermediate, which then undergoes reductive elimination to allow release of the isomerization product.

## Graphical abstract

**Using deuterium labelling**, electrochemistry, EPR and DFT, an iron catalyzed double bond isomerization reaction was disclosed that is likely to proceed through on‐cycle Fe^I^ and Fe^III^ intermediates. The reaction uses an Fe^II^ pre‐catalyst that undergoes a one‐electron reduction to get to the first on‐cycle species. These studies on mechanism, along with synthetic scope of the reaction, are presented.

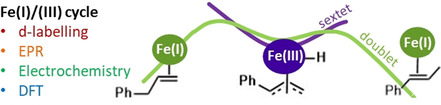

## Introduction

**Scheme 1:**
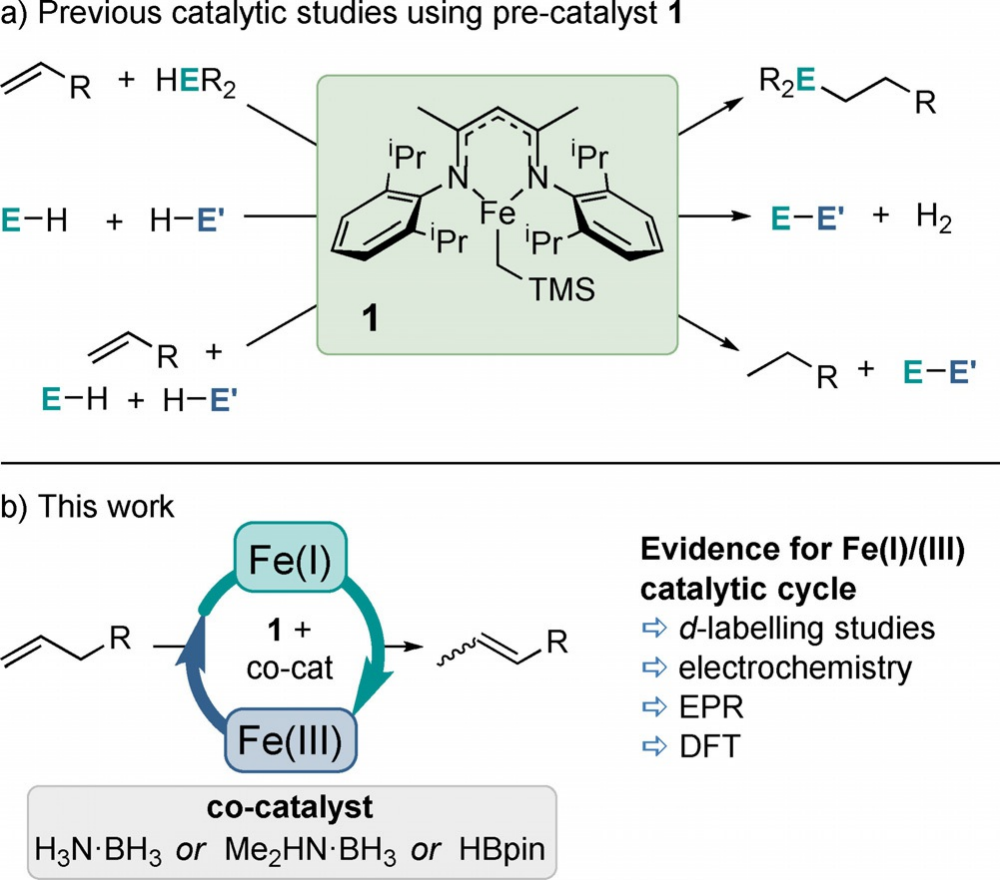
a) Previous catalytic reactions undertaken using **1**; b) this work.

Two‐electron chemistry within the field of iron catalysis is incredibly rare.^[1]^ We envisioned that if a controlled one electron reduction of an Fe(II) species could be carried out with a well‐defined iron pre‐catalyst, then it might be feasible to access an oxidative addition (OA)/reductive elimination (RE) (i.e. Fe^I^/Fe^III^) catalytic cycle to afford activity similar to that typically observed with precious metal congeners. Previous catalytic studies within our group using an iron(II) β‐diketiminate complex (hereafter referred to as complex **1**) have focused on bond forming transformations (for example hydrophosphination, hydrogenation and dehydrocoupling, Scheme 1 a). However, double bond isomerization gives the ideal opportunity to explore a unimolecular transformation that relies on a 1,3‐hydrogen shift,^[2]^ the simplicity of which may give increased likelihood of OA/RE chemistry (Scheme 1 b). Carbon–carbon double bonds are important in synthetic chemistry: they are used to introduce diverse functionality to organic molecules, including pharmaceuticals, and they are also used extensively in the petrochemical‐based polymer industry. Double bond isomerization can change the properties of a molecule without the need for stoichiometric exogenous reagents.^[3]^

Although there are several elegant examples of earth abundant metal‐catalyzed isomerization/functionalization^[4]^ (e.g. hydroboration,^[5]^ hydrosilylation,^[6]^ allylic alcohol to ketone^[7]^), which ultimately provide an energetically favorable driving force for the isomerization step, there are far fewer examples of the use of abundant metal pre‐catalysts to only invoke isomerization. Isomerization‐only processes catalyzed by iron are dominated by thermodynamically favored *cis*‐to‐*trans* and/or terminal to internal double bond isomerization. Some of the earliest examples of iron‐catalyzed reactions date back to the 1960’s when Frankel, Emken and Davison reported the use of Fe(CO)_5_ as a catalyst in double bond isomerization in fatty acids and esters.^[8]^ Triiron dodecacarbonyl was used by Casey and Cyr to undertake isomerization of 3‐ethyl‐1‐pentene and detailed mechanistic studies suggested that the reaction likely proceeded via an Fe(η^3^‐allyl)(H) intermediate.^[9]^ Beyond catalysis performed using iron carbonyl complexes,^[10]^ von Wangelin has reported isomerization using Fe(acac)_3_ with a Grignard additive at room temperature.^[11]^ More recently, Koh and co‐workers have reported an elegant regiodivergent isomerization process that uses B_2_pin_2_ or PhMe_2_SiBpin in conjunction with an iron catalyst and LiO*t*Bu. Mechanistic studies revealed that the reaction is likely to proceed via an iron‐hydride with subsequent olefin insertion and β‐hydride elimination.^[12]^ Pertinent to this study, Smith has employed an Fe^I^ species, exploiting the propensity for spin crossover, to achieve iron catalyzed double bond isomerization via a two‐electron catalytic cycle.^[13]^

From the synthetic studies we have already disclosed in hydrofunctionalization chemistry,^[14]^ we postulated that access to a two‐electron isomerization catalytic cycle would be possible by limiting the quantity of hydride source employed in order to access Fe^I^, meanwhile preventing competitive Fe^II^ hydroboration. We herein report evidence to support this hypothesis, using pre‐catalyst **1**.

## Results and Discussion

Following a short optimization procedure we found that a co‐catalytic quantity (10 mol %) of pinacol borane (HBpin), ammonia borane (H_3_N⋅BH_3_) or dimethylamine borane (Me_2_HN⋅BH_3_) are all similarly competent at transforming allyl benzene (**2 a**) into β‐methylstyrene (**3 a**) with 5 mol % **1** at 60 °C in 16 h (Table 1, also see Supporting Information for further optimization).

**Table 1:** Optimization of iron catalyzed double bond isomerization using allylbenzene as the substrate.

	Catalyst (5 mol %), additive (10 mol %)	Conversion to **3 a** (%)	*trans*:*cis*
1	**1**, none	0	0
2	None, HBpin	0	0
3	**1**, HBpin	93	7.6:1
4	**1**, H_3_N⋅BH_3_	99	25:1
5^[a]^	**1**, H_3_N⋅BH_3_	12	8:1
6	**1**, Me_2_HN⋅BH_3_	99	33:1
7^[b]^	**1**, TMP⋅BH_3_	73	3.6:1
8	**1**, PhH_2_N⋅BH_3_	44	2.9:1

Standard reaction conditions: C_6_D_6_ (0.6 mL), allylbenzene (0.5 mmol), catalyst (0.025 mmol), additive (0.05 mmol), 60 °C, 16 h. Conversion and selectivity determined by in situ ^1^H NMR spectroscopy. [a] RT. [b] TMP=2,2,6,6‐tetramethylpiperidine. 80 °C, 48 h.

**Scheme 2:**
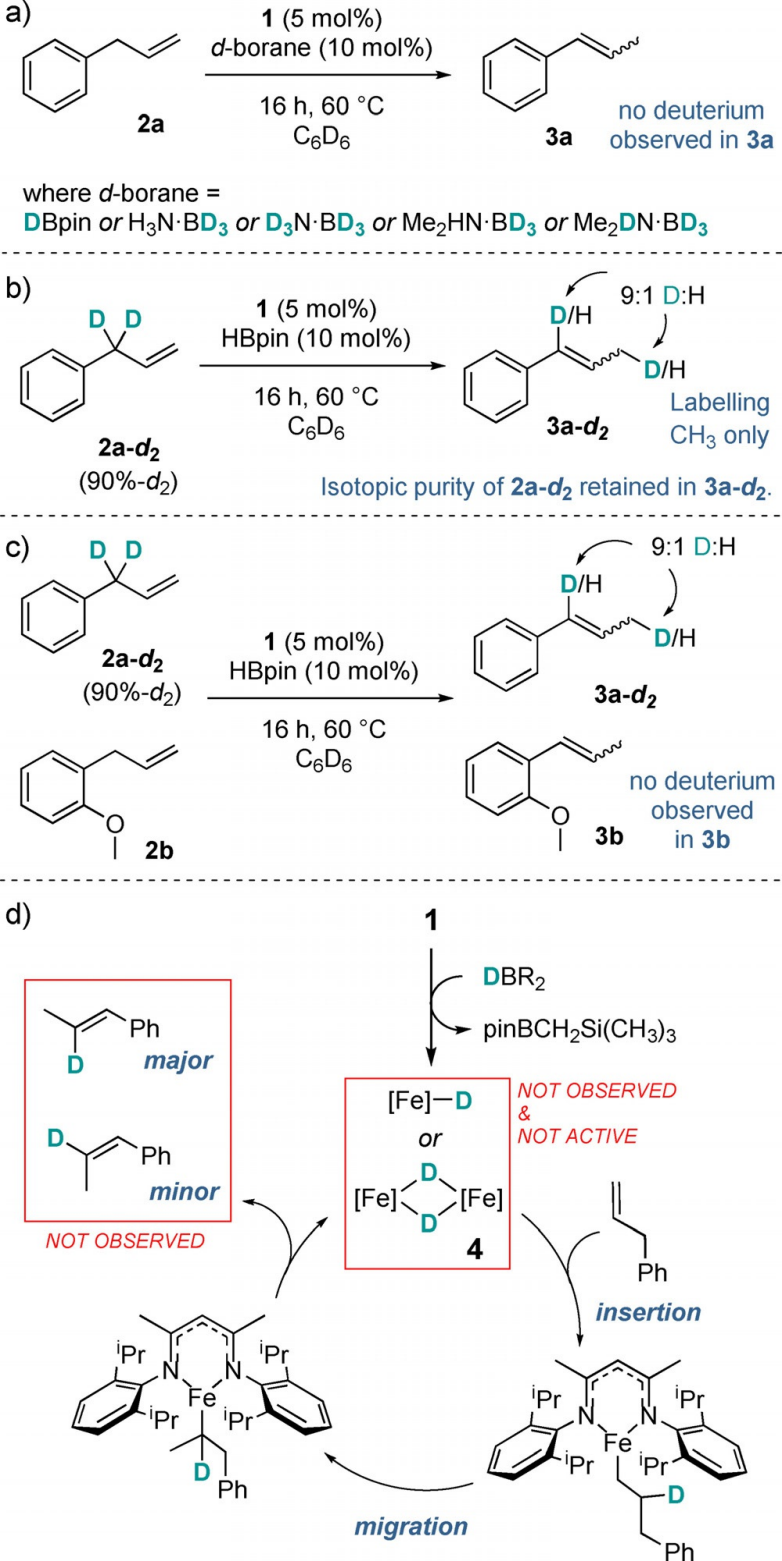
a)–c) Deuterium labelling studies; d) Discounted redox‐neutral catalytic isomerization cycle.

Our previous synthetic and theoretical studies with **1** have indicated that hydroboration^[14]^ and transfer hydrogenation^[15]^ reactions, amongst others, proceed in a redox‐neutral manner, in particular operating via an Fe^II^ hydride. In light of this, we sought to rule out an alkyl‐based mechanism where HBpin or an alternative reagent serves as a hydride source, forming an iron(II) hydride which can consequently undergo addition across a double bond and subsequent β‐hydride elimination at the more substituted position. Further investigation was able to discount this mechanism taking place. Firstly, the iron(II) hydride dimeric species **4** is poor in catalysis (16 % conversion to **3 a**,16 h, 60 °C as opposed to 93 % **3 a** with **1** and HBpin, see Scheme 2 d), contrasting with our dehydrocoupling work.^[16]^ Adding further hydride source to **4** does lead to catalysis, but the lack of NMR evidence for **4** forming in situ suggests it is not formed as part of a pathway to an active species. Secondly, deuteration studies give outcomes contradicting what would be expected for a redox neutral mechanism (Scheme 2).

**Scheme 3:**
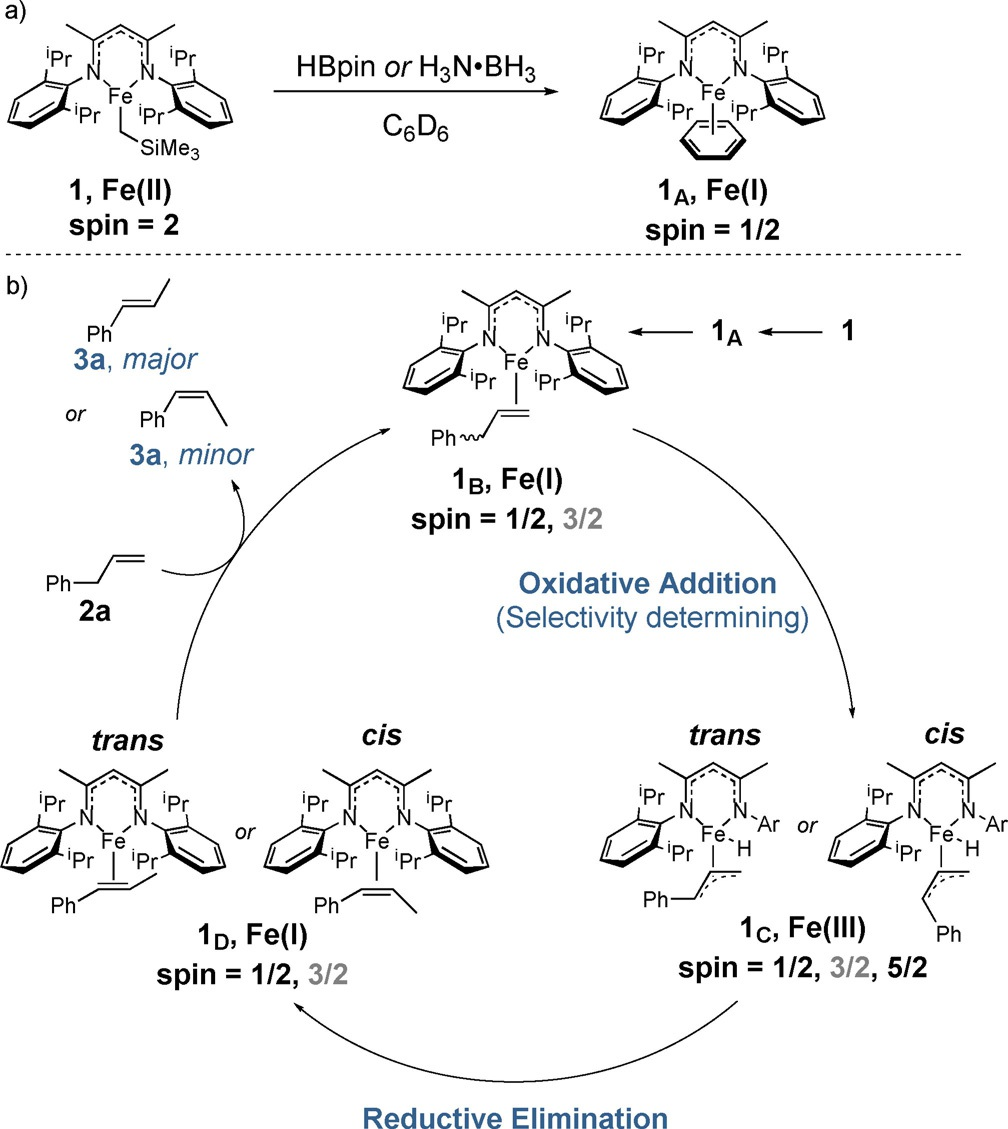
a) **1** reacts with borane to generate **1_A_**, which can then undergo further stabilization with alkene; b) proposed Fe^I^/Fe^III^ catalytic cycle for the isomerization of allylbenzene.

Firstly, there is no ^2^ h‐incorporation in substrates when using deuterated hydride sources (Scheme 2 a), and full isoretention is observed in the transformation of d‐labelled substrate **2 a**‐***d***_**2**_ (Scheme 2 b) to **3 a**‐***d***_**2**_. If **1** is activated to form an Fe‐hydride (or deuteride) then a small amount of H/D exchange would be expected owing to the intermolecular nature of catalyst activation and H transfer.

Secondly, in a competitive reaction of **2 a**‐***d***_**2**_ and **2 b**, isoretention is again observed (Scheme 2 c), indicating hydride transfer is intramolecular rather than intermolecular. At this stage, the deuterium labelling studies closely match the results one would expect from an η^3^‐allyl mechanism.^[3]^ Qualitatively, the reactions are observed to change from the dark yellow of **1** to a vivid red; a color that is synonymous with the formation of Fe^I^. Furthermore, upon investigating our proposed catalytic cycle with DFT calculations, we were able to demonstrate that a redox‐neutral Fe^II^ catalytic cycle of the form shown in Scheme 2 d is disfavored due to an exceptionally high energy barrier for the β‐hydride elimination step for the more stable, high‐spin quintet surface (Δ*G* ^≠^=37.4 kcal mol^−1^, see Supporting Information for details). Evans method NMR analysis^[17]^ gives μ_obs_=5.7 for the pre‐catalyst **1**, which is consistent with measurements from Hessen of similar Fe^II^ complexes.^[18]^ However, addition of HBpin, H_3_N⋅BH_3_ or Me_2_HN⋅BH_3_ to **1** results in a decrease in the magnetic moment to μ_obs_=5.1. We tentatively attribute this to a shift from pure Fe^II^ to a mix of oxidation/spin states being present. Adding **2 a** does not appear to affect μ_obs_ further. At this stage, we feel we have reasonable evidence to rule out the entirely redox‐neutral cycle of the form shown in Scheme 2 d. No reaction is observed with the radical clock 6‐bromo‐1‐hexene, therefore we are confident that there are no organic radicals present or propagating in solution.

**Figure 1:**
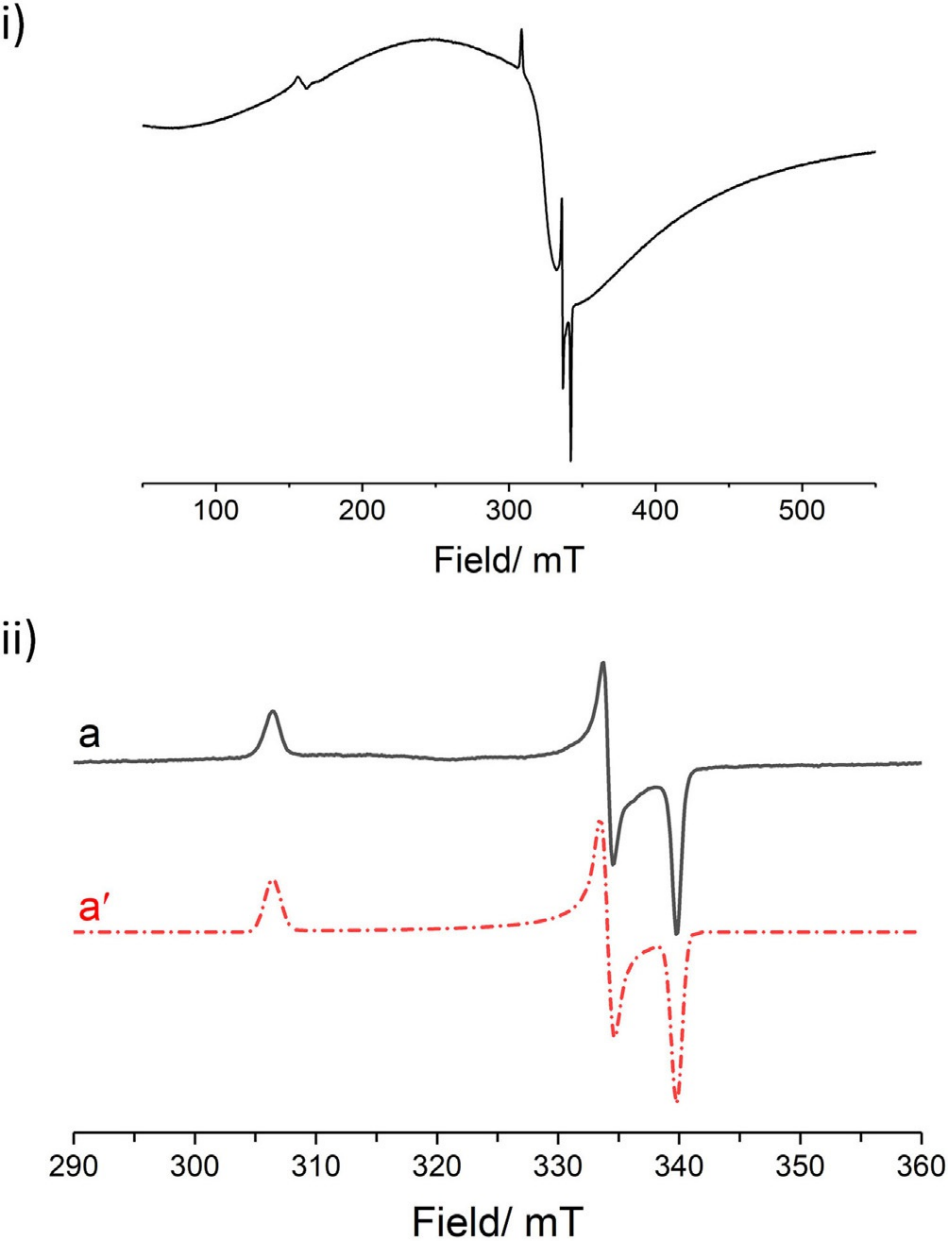
i) CW X‐band EPR spectrum [*T*=140 K] of **1** + HBPin. ii) Experimental (a), and simulation (a’) of CW X‐band EPR spectrum [*T*=140 K] of 1 + HBPin, focusing on the center‐field region. The broad signal originating from high‐spin Fe^III^ (seen in i) and also in Figure S1) has been background subtracted. See Supporting Information for corresponding simulations and discussion.

To support the NMR results, X‐band CW EPR studies were recorded in benzene:toluene (200:10 μL) frozen solution. A solution of **1** in the absence of substrate indicates the presence of a small amount of high‐spin Fe^III^, which we assign to the side formation of LFeCl_2_ during synthesis of the pre‐catalyst (see Supporting Information). Direct addition of **2 a** to **1** did not lead to any changes in the EPR spectrum (see Supporting Information), indicating no change in oxidation state induced by the alkene. However, upon addition of HBpin or H_3_N⋅BH_3_ to **1**, the appearance of a well‐resolved rhombic signal without any observable hyperfine coupling is observed, consistent with a low spin *S=*1/2 iron d^7^ center (Figure 1). Addition of **2 a** to this mixture does not lead to further spectral changes. Computer simulation of this species revealed the spin Hamiltonian parameters characterized by **g**=[1.984 2.018 2.200], which is analogous to Holland's characterization of an Fe^I^ species interacting with benzene, and our previous report of Fe^I^ complexes formed upon reaction of FeCl_3_ with aryl/alkyl Grignard reagents.^[19]^ The Fe^I^ signal is superimposed on a broad signal associated with a high‐spin Fe^III^ center, vide infra (also see Supporting Information for details). Cyclic Voltammetry was also used to determine whether HBpin, H_3_N⋅BH_3_ or Me_2_HN⋅BH_3_ are strong enough reductants to convert **1** into an Fe^I^ complex (see Supporting Information for details). Cyclic Voltammetry studies of **1** show two strong reduction peaks. We assign the first to the reduction of our Fe^II^ pre‐catalyst to an Fe^I^ species, which we determine to be −1.48 vs. Fc/Fc ^+^. The lack of a large peak in the oxidative direction suggests the irreversibility of the Fe^II^/Fe^I^ reduction process, potentially ruling out a one‐electron oxidation/reduction catalytic cycle. The second peak has a potential of −1.92 vs. Fc/Fc ^+^, and we believe it is ligand‐based and is unlikely to have significant bearing on the catalytic pathway. The relatively strong reducing potentials of both HBpin and H_3_N⋅BH_3_ indicate that such a reduction is thermodynamically feasible.^[20]^ Because of this, we propose that the active species in catalysis is initially an Fe^I^ species. This complex may be stabilized by either a molecule of solvent, as previously reported, or by a molecule of substrate. Substitution coefficient studies from Holland^[21]^ on **1_A_** (Scheme 3 a) indicate that, once formed, the η^2^‐allylbenzene complex **1_B_** is more stable, and we therefore believe that if **1_A_** forms, displacement to form **1_B_** is rapid and facile. Wide sweep width NMR spectroscopy of both stoichiometric and catalytic reactions (using HBpin or H_3_N⋅BH_3_) give complex spectra that we attribute to off‐cycle or catalytically inactive Fe^II^ complexes or Fe^III^ formed by disproportionation of Fe^II^ into Fe^I^ and Fe^III^ (see Supporting Information for extended discussion). Unfortunately, we have not been able to isolate any key intermediates from reaction mixtures^[22]^ and characterize them crystallographically, and detailed kinetics studies have been hampered by the reaction temperature coupled to the paramagnetic iron center, which have made in situ NMR monitoring challenging. However, in situ UV‐vis spectroscopy measurements give *λ*_max_=494 and 552 nm (for catalysis in the presence of H_3_N⋅BH_3_) or *λ*_max_=497 and 547 nm (for catalysis in the presence of HBpin); wavelengths that have been linked to both Fe^I^ and Fe^III^ β‐diketiminate species.[^21^, ^23^] Using the method reported by Scheer for the synthesis of the η^6^‐toluene analogue of **1_A_**,^[24]^ we obtain 87 % **3 a** (7.5:1 *trans*:*cis*) after 16 h at 60 °C when 5 mol % of this Fe^I^ species is used in catalysis (31 % in a 6.3:1 ratio after 2 h); these results are in‐line with those obtained using the same conditions with pre‐catalyst **1** (5 mol % 1 and 10 mol % HBpin, 27 %, 5.7:1 ratio after 2 h; and 93 %, 7.6:1 ratio after 16 h) and further support the proposal that the reaction proceeds via an Fe^I^ species.

To summarize the experimental results thus far: **1** appears to undergo a change in oxidation state in the presence of a hydride source and this is supported by NMR and EPR studies. Electrochemistry indicates that the hydride source can reduce **1** to an Fe^I^ species and work from Holland has demonstrated that alkenes are able to form η^2^‐Fe^I^ complexes (i.e. **1_A_** and **1_B_**). An η^6^‐arene Fe^I^ complex gives similar catalytic results to **1**. Deuterium labelling indicates that the hydride is not transferred to the isomerized product and therefore it appears to be acting as a pre‐catalyst activator only.

Having ruled out a redox‐neutral Fe^II^ pathway both experimentally and computationally, we further investigated the isomerization of allylbenzene to β‐methylstyrene using DFT calculations. We propose that the reaction proceeds from our active Fe^I^ η^2^‐alkene species **1_B_**, in the doublet spin‐state, via a selectivity‐determining oxidative addition of one of the phenyl adjacent C−H bonds to form either of Fe^III^ η^3^‐allyl species, **1_C_**_***trans***_ or **1_C_**_***cis***_. These intermediates then undergo a reductive elimination of the proton onto the terminal carbon to form the Fe^I^ allyl species, **1_D_**_***trans***_ or **1_D_**_***cis***_. A ligand exchange with the substrate **2 a** then yields the major product, *trans*‐β‐methylstyrene or the minor product, *cis*‐β‐methylstyrene, while also regenerating **1_B_** (Scheme 3 b).

We suggest that the doublet spin state of the complex is largely preserved throughout the catalytic cycle, although we have also investigated the other spin states, with a particular focus on the *trans*‐selective route. While the quartet **1_B_** was calculated to be more stable than the doublet by 21.0 kcal mol^−1^, we found, similarly to Smith,^[13]^ that the activation barrier for the *trans* selective oxidative addition on the quartet surface (Δ*G* ^≠^=45.0 kcal mol^−1^) was far in excess of what might be considered accessible under the experimental conditions used (see Supporting Information). In addition to this, our EPR results do not support the presence of a quartet Fe^I^ species, or a quartet Fe^III^ species.

Starting from our initial species **1_B_**, rotation of the Ph−C bond to give the appropriate orientation for each oxidative addition is a low‐energy process (<1 kcal mol^−1^), with the subsequent activation barriers (Δ*G* ^≠^=25.7 kcal mol^−1^ for *trans*, Δ*G* ^≠^=28.1 kcal mol^−1^ for *cis*) supporting the experimentally observed *trans* selectivity of the reaction (Table 2). Due to steric interactions arising from the proximity of the substrate phenyl ring to the 2,6‐diisopropylphenyl groups on the β‐diketiminate ligand, the *cis* η^3^‐allyl product of the oxidative addition, intermediate **1_C_**_***cis***_, generates an overall positive Δ*G* for that step (+19.7 kcal mol^−1^ relative to **1_B_**), while the less hindered **1_C_**_***trans***_ is 2.7 kcal mol^−1^ more stable than **1_C_**_***cis***_ and results in a slightly reduced free energy difference, Δ*G*=+18.0 kcal mol^−1^, making the formation of **1_C_**_***trans***_ slightly less reversible than **1_C_**_***cis***_. From there, both isomers have relatively small barriers for the reductive elimination (Δ*G* ^≠^=2.6 kcal mol^−1^ for *trans* and 4.8 kcal mol^−1^ for *cis*), the rate determining barrier for both is the oxidative addition, supported by a secondary kinetic isotope effect (*K*_D_/*K*_H_=1.03±0.06) observed experimentally. Both the *trans* (−4.6 kcal mol^−1^) and *cis* selective (−3.3 kcal mol^−1^) isomerization of allylbenzene are calculated to be exothermic overall.

**Table 2:** Calculated activation barriers Δ*G* ^≠^ (kcal mol^−1^) for the isomerization of allylbenzene for the proposed Fe^I^/Fe^III^ catalytic cycle.

Selectivity	Oxidative addition	Reductive elimination
***trans***	25.7	2.6
***cis***	28.1	4.8

**Figure 2:**
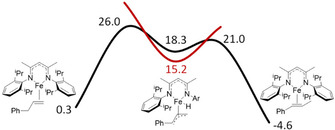
Calculated free energy surface for the *trans*‐selective isomerization of allylbenzene in doublet (black line) and sextet (red line) electronic configurations, relative to the initial on‐cycle species **1_B neutral_**. B3LYP‐D3/6‐31G(d), PCM=benzene, 298 K.

The sextet Fe^III^ intermediate **1_C_**_***trans***_ lies below the doublet electronic configuration at our chosen level of theory. As shown on the energy surfaces plotted in Figure 2, the high‐spin Fe^III^ species detected by EPR is likely to be **1_C_**_***trans***_ (3.1 kcal mol^−1^ more stable than the doublet), although we concede that computational method effects might affect this prediction. The observed reaction outcome could thus be explained either by considering only the low spin species, or by spin crossovers onto the high spin surface near the oxidative addition step and crossing back onto the low spin pathway near the reductive elimination. Unfortunately, we were unable to locate minimum energy crossing points (MECPs) between these surfaces with the method described by Harvey et al. (see Supporting Information for further details).^[25]^

**Scheme 4:**
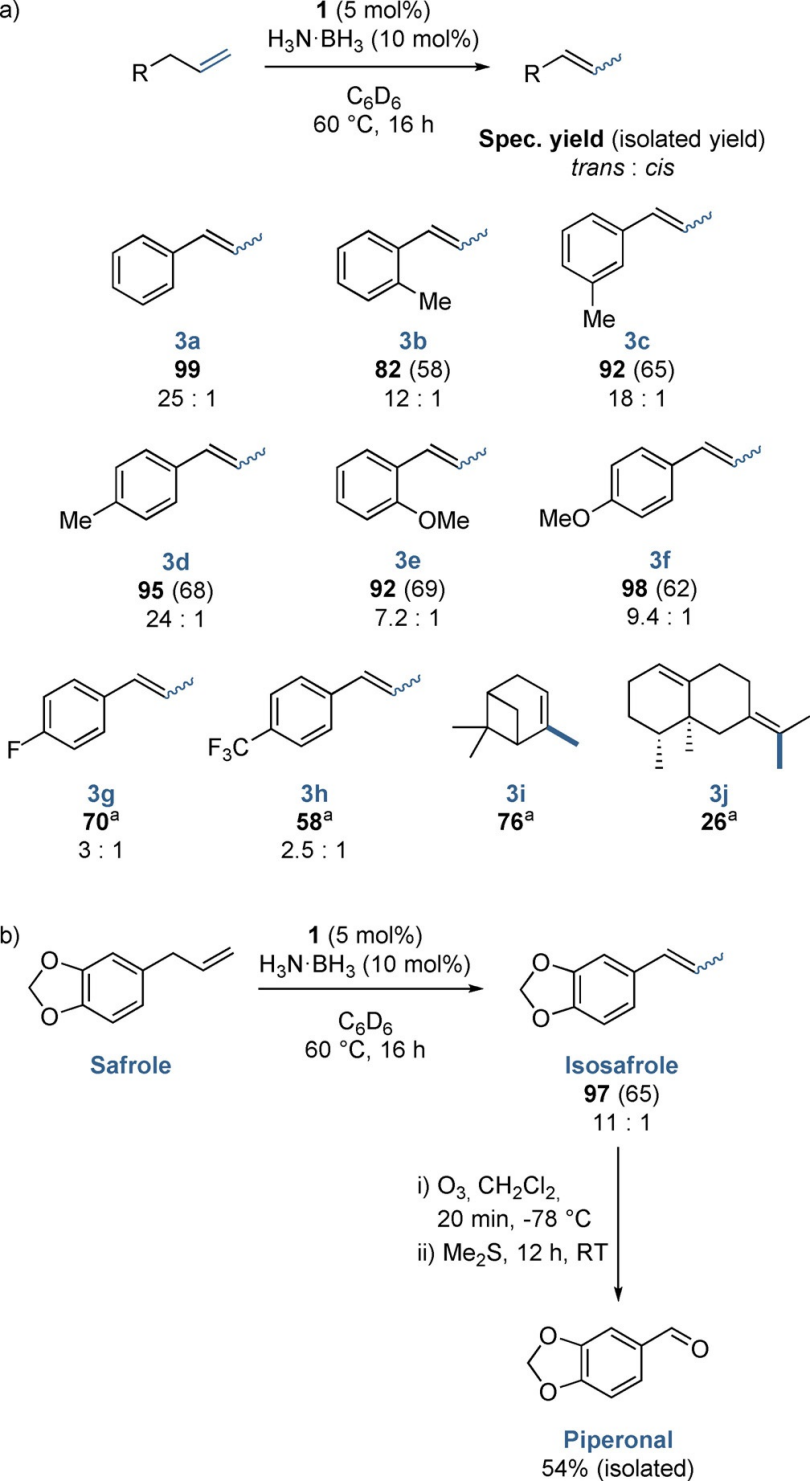
a) Double bond isomerization substrate scope. [a] 80 °C; b) Preparation of fragrance molecule piperonal using iron catalyzed double bond isomerization followed by ozonolysis.

To prove utility beyond allyl benzene we have explored a range of substrates (Scheme 4). We have opted to use H_3_N⋅BH_3_ as our hydride source as it is an easy to handle, inexpensive reagent with good sustainability credentials. In most cases there is good selectivity for the *trans*‐styrene product (**3 a** to **3 d**). However, when the aromatic ring is functionalized with strongly electron donating (**3 e** and **3 f**) or withdrawing groups (**3 g** and **3 h**), there is a drop‐off in *trans*‐selectivity and the *cis* product becomes more favorable. We link this, and the drop‐off in *trans* selectivity observed with HBpin (see Table 1), to rate of reaction: reactions of electron donating or withdrawing substrates, or those mediated by HBpin, are slower, meaning there is less opportunity for *cis*‐to‐*trans* isomerization to take place prior to product isolation. Good turnover is observed for the double bond isomerization of β‐ to α‐pinene (**3 i**) and a modest amount of *exo*‐ to *endo*‐double bond isomerization is observed for valencene (**2 j**). Unfortunately, no reactivity is observed with secondary and tertiary amine‐containing alkenes (diallylamine, *N*‐allylaniline and 4‐methyl‐diallylaniline), or with 1,1‐disubstituted alkenes such as (2‐methyl‐2‐propenyl)benzene or multiple double bond containing terpenes such as β‐farnesene (which showed good reactivity during hydroboration studies).^[14]^ We have explored the selectivity obtained for the isomerization of hexene (see Supporting Information) but more forcing reaction conditions are required (80 °C, 48 h, in comparison to 60 °C, 16 h).

Finally, to demonstrate the applicability of this isomerization catalysis to sustainable chemistry, we have utilized our catalysis to afford a key synthetic fragrance and flavor molecule (Scheme 4 b). In a reaction sequence involving iron‐catalyzed double bond isomerization followed by ozonolysis, we have transformed safrole into piperonal, a floral/vanilla scented compound with both fragrance and synthetic applications.

## Conclusions

In summary, we have reported a rare example of iron catalysis likely proceeding through a two‐electron oxidation/reduction mechanistic pathway. A catalytic amount of HBpin or H_3_N⋅BH_3_, rather than act as a source of hydride, acts to reduce our Fe^II^ pre‐catalyst to an Fe^I^ species, a proposal supported by EPR and electrochemical studies. An η^3^‐allyl‐type mechanism, proceeding via an Fe^I^/Fe^III^ redox active cycle, is supported by deuterium labelling and DFT studies, leading to terminal alkenes being isomerized to more substituted products in a *trans* selective manner. The presence of a redox active catalytic cycle raises the question of whether this chemistry is limited to double bond isomerization, or whether we can exploit this to other two‐electron transformations.

## Conflict of interest

The authors declare no conflict of interest.

## Supporting information

As a service to our authors and readers, this journal provides supporting information supplied by the authors. Such materials are peer reviewed and may be re‐organized for online delivery, but are not copy‐edited or typeset. Technical support issues arising from supporting information (other than missing files) should be addressed to the authors.

Supplementary
