# Supplementary material for: Iron Catalyzed Double Bond Isomerization: Evidence for an FeI/FeIII Catalytic Cycle
Source: Chemistry. 2021 Mar 15;27(19):5972–7. doi: 10.1002/chem.202004980 (PMC8048803; doi:10.1002/chem.202004980)
Supplement: Supplementary file 1 — Supplementary [file CHEM-27-5972-s001.pdf]

Chemistry–A European Journal

Supporting Information

Iron Catalyzed Double Bond Isomerization: Evidence for an Fe^{I}/Fe^{III} Catalytic Cycle

Callum R. Woof,[a] Derek J. Durand,[b] Natalie Fey,*[b] Emma Richards,*[c] and Ruth L. Webster*[a]

Contents

1. General Experimental Details 2
2. Synthesis of Ligands and Complexes 5
3. Synthesis of Boranes 8
4. Synthesis of Deuterated Substrates 9
5. Extended Optimization Data 12
6. Studies with Linear Alkenes 13
7. Characterisation of Products 13
8. Deuteration Experiments & Characterisation 18
9. Electrochemistry Experimental Data 25
10. NMR Spectra 28
11. EPR Spectroscopy 56
12. UV-Vis Spectroscopy 60
13. Further Studies into Catalyst Activation and Active Species 60
14. Computational Studies 66
    Computational Details: 66
    Fe (I/III) Mechanism: 67
    Computational Method Testing for Fe(I/III) catalytic cycle: 71
    Fe (II) mechanism: 74
15. References 76

1. General Experimental Details

Reagents were purchased from Sigma Aldrich or Acros and distilled before use. Pentane was purchased from Fisher and used without further purification (except for crystallisations). Solvents (e.g. C_6D_6) were dried with sodium/benzophenone before use. NMR data was collected on 300, 400 or 500 MHz Bruker or Agilent machines at 298 K and referenced to residual protic solvent (benzene for spectroscopic yields, chloroform for isolated products). All manipulations were carried out under an inert atmosphere using standard Schlenk/glovebox techniques unless stated.

General reaction setup

Experiments were performed under an argon atmosphere in an M-Braun glove box. The iron pre-catalyst was weighed, dissolved in dry deuterated benzene solvent and added to a J-Young tap NMR tube. To this was added pinacolborane / ammonia borane or alternative hydride source, followed by the substrate. The tube was sealed and the reaction allowed to proceed for the time and temperature stated. The product was isolated by exposing the reaction to air, careful removal of most of the solvent by passing nitrogen over the mixture, followed by filtration through a silica plug using pentane as the solvent. Pentane was removed by blowing nitrogen over the eluate.

Synthesis of piperonal from isosafrole

144 µL of isosafrole (**2K**, prepared from standard procedure) was dissolved in approximately 4 mL dichloromethane in a 250 mL round bottom flask, which was then cooled to – 78 °C. Ozone (supplied from a Wallace & Tiernan Ozone generator at 230 V) was then bubbled through the reaction for 20 minutes, at which point the solution turned from colourless to dark blue. Following this, the reaction was bubbled through for 20 minutes with oxygen and 1 hour with nitrogen, at which point the reaction turned colourless again. Dimethyl sulphide (3.1 eq., 165.7 µL, 196 mg) was then added and the reaction was stirred at room temperature overnight. The solution was washed with saturated sodium carbonate solution, followed by saturated brine solution twice, and the solvent (and acetaldehyde side-product) was removed under reduced pressure to leave piperonal as a white solid (81 mg, 54% yield)

Nanoparticle Test

Adapted from the procedure reported by Morris. [1] Reactions were set up under standard conditions but trimethylphosphine (1.3 µL, 2.5 mol%, 0.5 equiv. with respect to catalyst) was also added. In a nanoparticle-based system, the reaction would be expected to be fully quenched with a sub-stoichiometric amount of PMe_3 sufficient to bind to all active sites. In reactions with both pinacolborane and ammonia borane conversion was reduced but still present under standard reaction conditions, indicating nanoparticles are not active in catalysis.

Blank Reactions

To a solution of allylbenzene (66.1 µL, 0.5 mmol) in 600 µL of C_6D_6 was added 1.5 mg (0.05 mmol, 0.1 eq) of amine borane. After heating at 80 °C for 48 hours, no reactivity was observed. The same outcome was observed when 7.2 µL (0.05 mmol, 0.1 eq) of HBpin was used

To a solution of 14 mg (0.025 mmol) of catalyst **1** in 600 µL of C_6D_6 was added 66.1 µL (0.5 mmol, 20 eq) of allylbenzene. After heating at 80 °C for 48 hours, no reactivity was observed.

Evans Method Setup

Measurements were performed on a Bruker 500 MHz NMR spectrometer, in a J-Young NMR tube under an argon atmosphere with a sealed C_6D_6 capillary inserted. To 600 µL C_6D_6 was added the required solid (between 3-5 mg, accurately weighed). The magnetic susceptibility was determined by the difference in ^{1}H NMR chemical shift of the residual protic peaks of benzene-d_6 in the solution and the capillary.

Kinetic Reactions

Experiments were set up as per standard reaction setup, with the addition of 1,3,5-trimethoxybenzene (15-20 mg, accurately weighed) to act as an internal standard. The sealed reaction vessel was monitored in a Bruker 400 MHz NMR Spectrometer, with measurements taken every 10 minutes at room temperature for two hours.

Substrate	Rate
$PhCHCHCH_2$ **2a**	2.91×10^{-3} min^{-1}
$PhCHCHCD_2$ **2a-terminal-d$_2$**	2.82×10^{-3} min^{-1}

2a

2a-terminal-*d*$_2$

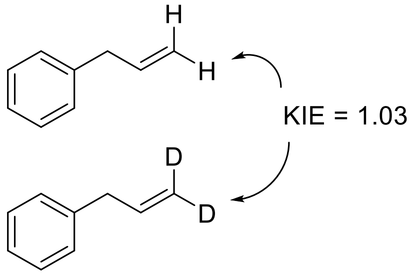

The secondary kinetic isotope effect of this reaction was therefore determined to be 1.03 ± 0.06

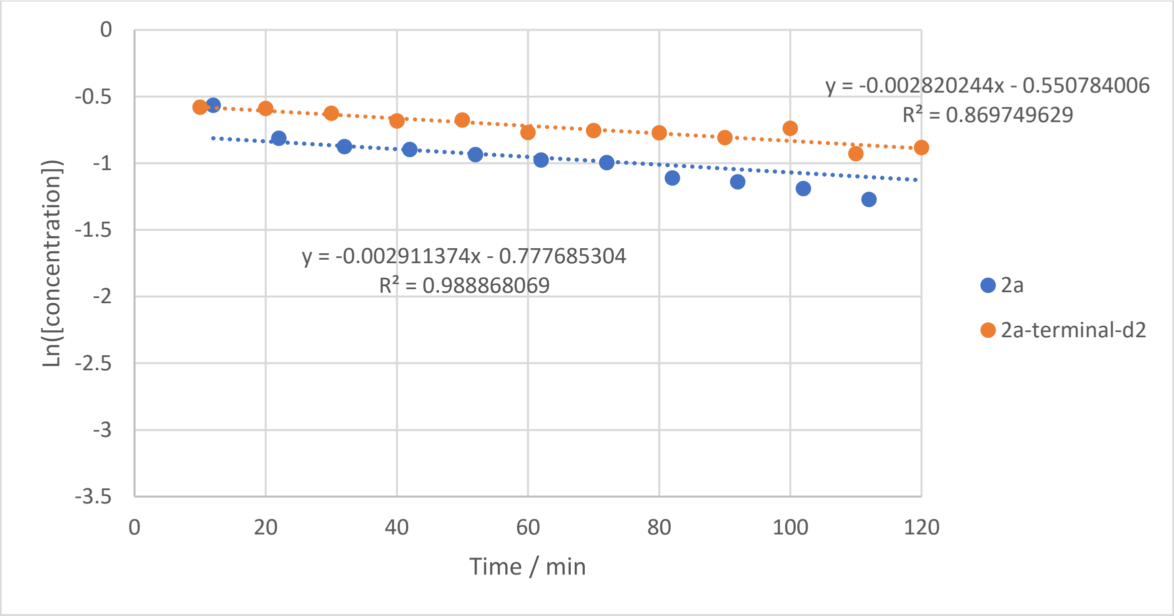

Test for activation of catalyst using H_2

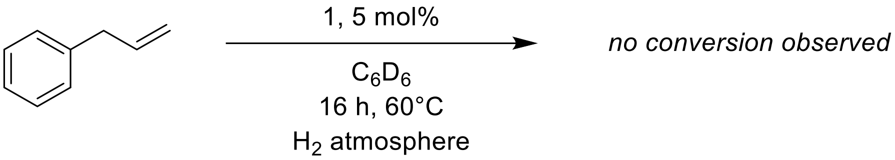

Allylbenzene (66.1 µL, 0.5 mmol) and **1** (14.0 mg, 0.025 mmol, 5 mol%) were added to 600 µL benzene-d_6 in a J-Young NMR tube under an argon atmosphere in a glovebox as per the standard reaction setup. The reaction vessel was then removed from the glovebox and the atmosphere removed by freeze-pump-thaw technique on a high-vacuum Schlenk line three times. The vessel was then purged with 1 atm hydrogen gas, sealed, and subject to standard reaction conditions (16 h, 60°C). Following this, no reactivity was observed. ^{1}H NMR spectrum only indicate allylbenzene present in the δ = 0-10 ppm region

^{1}H NMR

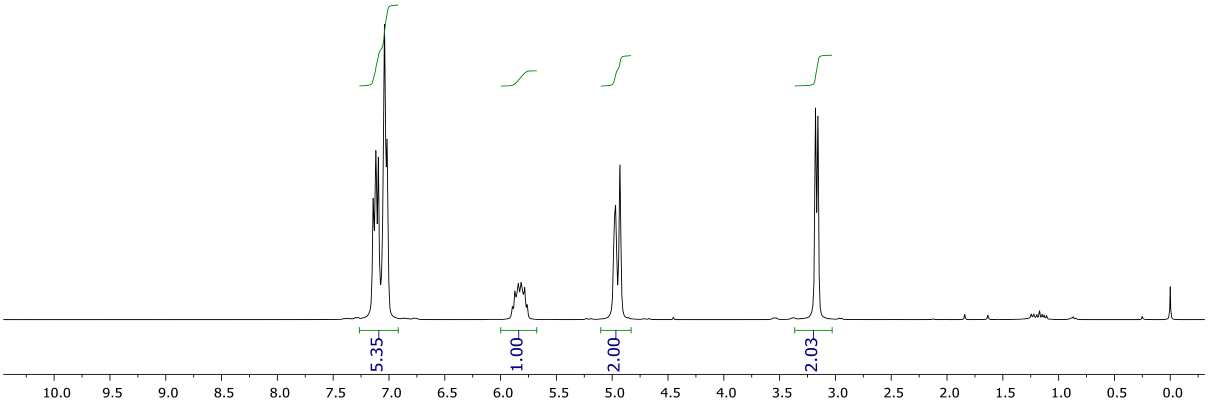

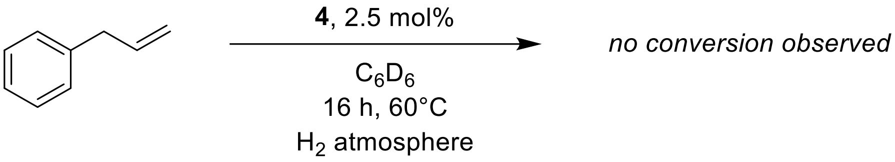

To further discount the role of iron hydrides in catalysis, the same experiment was repeated, with the exception of using the dimeric Fe(II) hydride **4** (11.8 mg, 0.0125 mmol, 2.5 mol%) in place of **1**. In this case, a small amount of isomerisation is observed (approx. 7%) which is similar to the same reaction under an argon atmosphere. There is also a small amount of conversion to the hydrogenated product (approx. 9 %)

^{1}H NMR (the isomerised products are identified with ●, the hydrogenated products with ●)

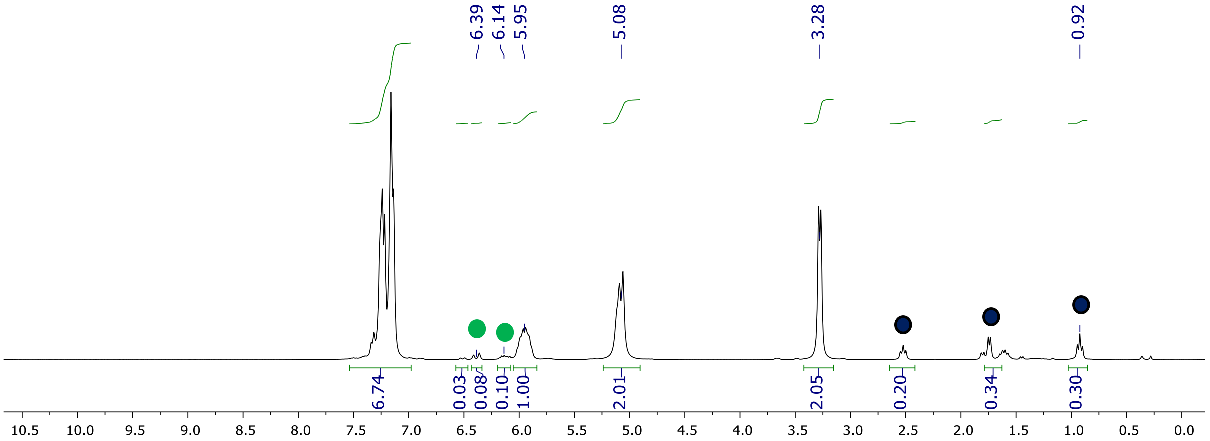

Further discussion on the setup of the hydrogen supply is provided by Whittlesey et.al. (J. Am. Chem. Soc. 2020, 142, 6340–6349). The authors would like to thank the group of Professor Mike Whittlesey for assistance with these experiments.

Radical Clock Test

We have previously reported this radical clock test for other catalytic reactions, for an example see ACS Catal. 2016, 6, 11, 7892–7897. The reaction was prepared under normal reaction conditions using HBpin (10 mol%). The reaction was allowed to proceed for 4 hours at 60 °C, at which point conversion to product was observed to be partial (28%). (Chloromethyl)cyclopropane (0.025 mmol, 4.6 µL) was then added to the reaction and heating was continued. Although reactivity was perturbed, catalysis still proceeded beyond addition of the radical trap, and the ring opened product expected from a radical system (1-butene) was not observed, only the initial (chloromethyl)cyclopropane (after a further 4 hours 44% conversion).

2. Synthesis of Ligands and Complexes

(2,6-diisopropylphenyl)imino)pent-2-en-2-yl-2,6-diisopropylaniline

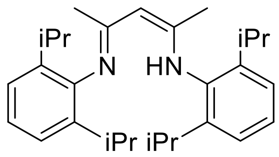

As previously reported, [2] in a two-neck 500-mL round-bottomed flask 2,4-pentanedione (6.68 g, 0.067 mol) was mixed with 300 mL of ethanol and 2,6-diisopropylaniline (28.67 g, 0.162 mol). To the mixture was added 7.5 mL of 12 M hydrochloric acid and the solution was refluxed with vigorous stirring for 3 days. The resulting slurry was then allowed to cool to room temperature and filtered. The filtered solid was dried under reduced pressure, and the filtrate was evaporated on a rotary evaporator. The dried mass was combined with the filtrate residue and the mixture was refluxed in 250 mL hexane at 80 °C for 1 h. The mixture was then cooled and filtered. Next the solid residue was treated with 300 mL of a saturated aqueous solution of Na_2CO_3 and 500 mL of Dichloromethane (DCM). The slurry was stirred until the solid dissolved. Stirring was then ceased giving a yellowish organic solution and a pale yellow aqueous layer. The organic layer was separated using a separatory funnel and then dried over $MgSO_4$. The solution was filtered and dried under reduced pressure to yield an off-white solid that upon washing with 50 mL of cold methanol (-20 °C) yields the desired proligand as a white powder.

^{1}H (500 MHz, $CDCl_3$) δ 12.11 (s, 1H, N*H*), 7.11 (m, 6H, aryl-H), 4.85 (s, 1H, C*H*), 3.11 (hep, 4H, C*H*(CH_3)$_2$, ^{3}J = 6.9 Hz), 1.71 (s, 6H, NCC*H*$_3$), 1.21, (d, 12H, CH(C*H*$_3$)$_2$, ^{3}J = 6.9 Hz), 1.11, (d, 12H, CH(C*H*$_3$)$_2$, ^{3}J = 6.9 Hz)

^{13}C{^{1}H} (126 MHz, $CDCl_3$) δ 161.1, 142.4, 140.1, 125.0, 123.1, 93.1, 28.2, 24.2, 23.2, 20.7

Data is concordant with previous literature [3]

Synthesis of Precatalyst 1

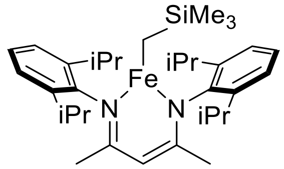

(1)

As previously reported, [3] n-Butyl lithium (solution in hexane, 1.67 mL, 4.18 mmol) was added to a Schlenk containing a stirred solution of β-diketimine (1.75 g, 4.18 mmol) in tetrahydrofuran (THF) (20 mL), at -78 °C. This was then allowed to warm to room temperature and the resulting pale yellow solution was stirred for 30 mins. Next solid $FeCl_2(THF)_{1.5}$ (982 mg, 4.28 mmol) was added to the solution. The resulting yellow solution was stirred for 45 min. Solid $LiCH_2SiMe_3$ (394 mg, 4.18 mmol) was then added and a very dark orange solution was obtained. After 30 mins the solution had turned dark red. After another 15 mins stirring, the solvent was removed in vacuo. Any residual THF was then removed by stirring the residue with pentane (3 x 25 mL) and subsequent evaporation of all volatiles. Next, the residue was extracted with pentane via filtration through a pad of celite. The yellow/orange extract was

concentrated to approximately 10 mL and cooled to -25 °C to afford **1** as yellow crystals (1.28 g, 58% yield).

Synthesis of Iron Hydride 4

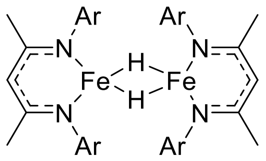

(4)

Iron hydride was prepared as reported previously by our research group.[4]

Synthesis of (NACNAC)Fe-Toluene-μ6

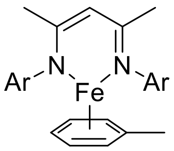

(NACNAC)Fe-Toluene-μ6 was prepared using the method reported by Scheer. [5] A yellow slurry of 6.68 g (24.0 mmol) (2,6-diisopropylphenyl)imino)pent-2-en-2-yl-2,6-diisopropylaniline in 100 mL THF was treated with a solution of 15 mL (24.0 mmol) nBuLi (1.6 M in n-hexane). The formed clear red solution was stirred at room temperature for 1 hour. The solution was slowly transferred into a slurry of anhydrous $FeCl_2$ (3.04 g, (24.0 mmol) in 5 mL THF, forming an intense dark yellow solution, which was stirred at room temperature for 12 h. After removal of solvent, the brownish solid was dissolved in 50 mL of toluene. The intense dark yellow solution was transferred into a slurry of 1.05 equivalents of potassium graphite in 10 mL toluene. The mixture was stirred at room temperature for 98 hours and a color change to olive green was observed. Remaining graphite and salts were removed via filtration of the olive-green solution over celite. The solvent was removed under reduced pressure and a dark green brown solid was obtained. The solid was dissolved in 100 mL n-hexane and the solution was filtered over celite, with the desired dark green solid precipitating at -20°C under reduced pressure.

The ^{1}H NMR was found to be very broad in both toluene-d_8 and benzene-d_6 – the data below is reported in toluene-d_8 to prevent any toluene-benzene ligand exchange. The spectrum is referenced to 1,3,5-trimethoxybenzene (OMe δ = 3.84 ppm) – the aryl peak is subsumed by the metal complex signals. No peaks were observed in the far downfield region (512.9 ppm and 487.6 ppm) as reported.

^{1}H (500 MHz, C_7D_8) δ 10.7 (3H), 7.60 (7H), 2.56 (3H), 1.73 (12H)

Literature Data

^{1}H (C_7D_8) δ 512.9 (2H), 487.6 (1H), 10.9 (4H), 9.8 (2H), 7.76 and 7.65 (ca. 5H), 2.78 (3H), 1.9 (12H)

Use of Fe(I) complex in catalysis

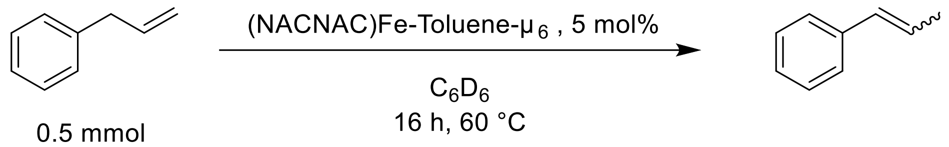

The reaction was as per the standard reaction setup, with the exception of 11 mg of (NACNAC)Fe-Toluene-μ_6 in place of pre-catalyst and hydride source. Conversion and selectivity after both 2 hours and 16 hours were very similar to the conversion and selectivity when using HBpin (Table 1, Entry 3) under the same conditions (see table below)

Time	Conversion (%), Selectivity (*Trans* : *Cis*)	
	(NACNAC)Fe-Toluene-μ_6, 5 mol%	**1**, 5 mol% & HBpin, 10 mol%
2 h	31 (6.3 : 1)	27 (5.7 : 1)
16 h	87 (7.5 : 1)	93 (7.6 : 1)

3. Synthesis of Boranes

Deuterated pinacolborane was synthesised as previously reported. [4] Deuterated amine boranes were synthesised as previously reported. [6]

Tetramethylpiperidine borane ($C_9H_{19}N{\cdot}BH_3$)

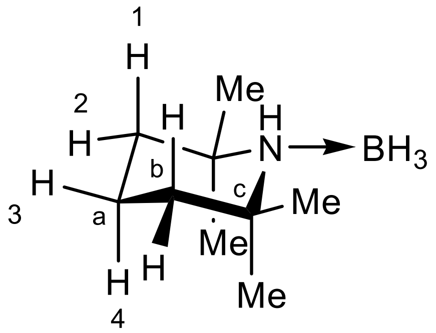

The synthesis was adapted from previous methodology. [7] Tetramethylpiperidine (TMP) (4 mmol, 565 μL) was placed in a Schlenk flask under an atmosphere of N_2. The vessel was then cooled to -78 °C and a solution of $BH_3{\cdot}THF$ in THF (1 M, 6 mmol, 6 mL) was added slowly dropwise, with stirring. The solution was then stirred at room temperature for 16 hours. The solvent and excess $BH_3{\cdot}THF$ were removed under vacuum, affording the product as a white solid

^1H (500 MHz, C_6D_6) δ 2.55-1.88 (N*H* and B*H*$_3$, 4H), 1.21 (H_3, dt, $^3J_{eq\text{-}eq}$ = 13.7 Hz, $^3J_{eq\text{-}ax}$ = 3.3 Hz, 1H), 1.15 (Me, s, 12H), 1.05 (H_1, dt, $^3J_{ax\text{-}ax}$ = 14.1 Hz, $^3J_{ax\text{-}eq}$ = 3.3 Hz, 2H), 0.88 (H_4, dt, $^3J_{ax\text{-}ax}$ = 14.1 Hz, $^3J_{ax\text{-}eq}$ = 3.7 Hz, 1H), 0.68 (H_2, td, $^3J_{eq\text{-}eq}$ = 13.7 Hz, $^3J_{eq\text{-}ax}$ = 3.7 Hz, 2H)

$^{13}C\{^1H\}$ (126 MHz, C_6D_6) δ 40.2 (C_b), 33.4 (Methyl-C), 20.2 (C_c), 16.2 (C_a)

^{11}B (160 MHz, C_6D_6) δ -20.7 (q, $^1J_{HB}$ = 99 Hz)

Data is concordant with previous literature [6, 8]

4. Synthesis of Deuterated Substrates

Synthesis of Wittig Reagents

Triphenylphosphine (5.62 g, 21.4 mmol) was dissolved in 40 mL dry THF in a flame-dried Schlenk flask under a nitrogen atmosphere. Iodomethane (1.60 mL, 3.65 g, 25.7 mmol) was added dropwise, and the flask was then sealed and heated to 70 °C for 1 hour. A white precipitate was observed to form. The precipitate was filtered on a Buchner funnel and washed with xylenes. Vacuum drying for 1 hour led to a white powder (8.68 g, 99% yield). Spectroscopic data was consistent with commercial material (Acros, 99%)

For the preparation of CD_3PPh_3I, an identical preparation was used with the exception of using iodomethane-d_3 (1.61 mL, 3.67 g, 25.7 mmol) in place of iodomethane (8.67 g, 97% yield, 98% d-incorporation)

NMR data for CD_3PPh_3I

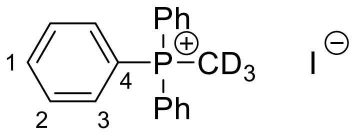

^{1}H (500 MHz, $CDCl_3$) δ 7.86-7.64 (ar, m, 15H)

^{13}C{^{1}H} (126 MHz, $CDCl_3$) δ 135.4 (C_1, d, ^{4}J = 3.0 Hz), 133.5 (C_3, d, ^{2}J = 10.9 Hz), 130.6 (C_2, d, ^{3}J = 12.9 Hz), 119.0 (C_4, d, ^{1}J = 88.7 Hz), 25.8 (Me, s)

^{2}H (77 MHz, $CDCl_3$) δ 3.21 (s)

^{31}P{^{1}H} (202 MHz, $CDCl_3$) δ 22.5 (s)

Synthesis of 2,2-d$_2$-phenylacetic acid

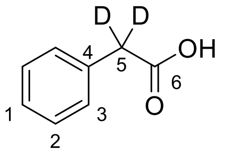

Adapted from the procedure reported by Gao *et.al.* [9] In a J-Young-Schlenk flask were added 6.8 g (50 mmol) of phenylacetic acid and 20 mL of 3.5 M sodium deuteroxide solution in D_2O, under air. The flash was sealed and heated to 100 °C overnight with stirring. After cooling, 20 mL of 4 M hydrochloric acid solution was used to neutralise the mixture. The crude product was extracted with 30 mL of dichloromethane, dried with magnesium sulphate and the solvent was removed under reduced pressure. The process was then repeated with the crude product to give 2,2-d$_2$-phenylacetic acid with 97% deuterium incorporation (98% yield, 6.72 g).

^{1}H (500 MHz, $CDCl_3$) 7.26-7.37 (ar, m, 5H)

^{13}C{^{1}H} (126 MHz, $CDCl_3$) 178.4 (C_6), 133.3 (C_4), 129.5 (C_3), 128.8 (C_2), 127.5 (C_1), 40.7 (C_5)

^{2}H (77 MHz, $CHCl_3$) 3.72 (s)

FTMS (NSI) $[2M-2H+Na]^-$ 297.1046 (calc) 297.1045 (obs) $[2M-H]^-$ 275.1227 (calc) 275.1226 (obs)

Data is concordant with previous literature [9]

Synthesis of 2,2-d$_2$-2-phenylethanol

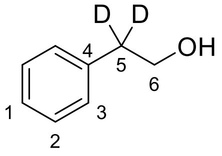

1.64 g of lithium aluminium hydride was suspended in 50 mL of (SPS dried) tetrahydrofuran and cooled to 0 °C. To this was added dropwise a solution of 2,2-d$_2$-phenylacetic acid (5 g, 36 mmol) in 40 mL tetrahydrofuran. The mixture was stirred for 90 minutes and quenched with 0.5 M hydrochloric acid. The mixture was then filtered and washed with ethyl acetate. The organic phase of the eluent was washed with both water and brine, dried with magnesium sulphate and concentrated under reduced pressure. The crude product was then purified with column chromatography (5:1 petroleum ether:ethyl acetate). Careful rotary evaporation yielded the purified product containing some residual ethyl acetate (Floral-smelling clear liquid 4.0 g, 90% yield)

^{1}H (300 MHz, $CDCl_3$) 7.37-7.21 (ar, m, 5H), 3.86 ($PhCD_2C\mathit{H_2}OH$, br s, 2H), 1.51 ($PhCD_2CH_2O\mathit{H}$, s, 1H)

^{13}C{^{1}H} (126 MHz, $CDCl_3$) 138.5 (C_4), 129.2 (C_2), 128.7 (C_3), 126.6 (C_1), 63.7 (C_6) (C_5 not observed)

^{2}H (77 MHz, $CDCl_3$) 2.86 (s)

Data is concordant with previous literature [9]

Synthesis of 2,2-d$_2$-phenylacetaldehyde

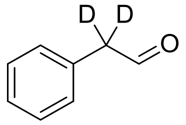

Pyridinium chlorochromate (1.6 g, 7.5 mmol) and 7.5 g of silica gel were mixed in 15 mL dichloromethane and 2,2-d$_2$-2-phenylethanol (0.62 g, 5 mmol) dissolved in 10 mL dichloromethane was added; the resultant mixture was stirred for 5 hours. Following this, the reaction was filtered through a silica plug and washed through with diethyl ether, and the solvent was removed to give the crude 2,2,-d$_2$-phenylacetaldehyde product. (Clear oil 0.65 g, 97% yield)

^{1}H (500 MHz, $CDCl_3$) 9.75 ($PhCD_2C\mathit{H}O$, s, 1H) 7.18-7.41 (ar, m, 5H)

MS [M-H] 121.0701 (theoretical) 121.0635 (obs)

Synthesis of 1,1-d$_2$-allylbenzene (2a-d$_2$)

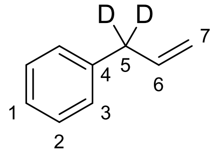

To a stirred solution of methyltriphenylphosphonium iodide (5.5 mmol, 2.22 g) in 10 mL dry THF was added n-butyl lithium solution (6 mmol, 1.54 M in hexanes, 3.89 mL) under a nitrogen atmosphere. The solution was cooled to 0 °C and crude phenyacetaldehyde-2-2-d_2 (566 µL, 0.61 g, 5 mmol) was added. The reaction was then allowed to warm to room temperature and stirred for 12 hours. The reaction was then quenched with 0.5 M HCl, and the product was extracted with diethyl ether and washed with water. The organic solution was concentrated to give a crude product, which was purified through column chromatography (pentane:ethyl Acetate 9:1). Careful rotary evaporation yielded the deuterated substrate with a small amount of remaining ethyl acetate (Clear yellow liquid, 0.49 g, 82% yield, 90% deuterium incorporation)

^{1}H (300 MHz, $CDCl_3$) 7.36-7.17 (ar, m, 5H) 5.97 ($PhCD_2C\mathit{H}CH_2$, m, 1H) 5.09 ($PhCD_2CHC\mathit{H_2}$, m, 2H)

^{13}C{^{1}H} (126 MHz, $CDCl_3$) 137.4 (C_4), 128.6 (C_3), 128.4 (C_2), 126.0 (C_6), 125.8 (C_1), 115.7 (C_7), 16.2 (C_5)

^{2}H (77 MHz, $CHCl_3$) 3.36 (s)

TOF-MS [M-] 129.0908 (theoretical) 129.0918 (observed)

Data concordant with previous literature [9]

Synthesis of 3,3-d_2-allylbenzene (2a-d_2-terminal)

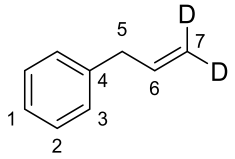

Methyltriphenylphosphonium iodide-d_3 (8 mmol, 3.25 g) was dissolved in 30 mL dry THF in a flame-dried J-Young Schlenk and cooled to -78 °C. To this solution, n-butyl lithium (8 mmol, 1.43 M in hexanes, 5.59 mL) was added dropwise, and the reaction was allowed to stir for 15 minutes before warming to room temperature. Following a further 30 minutes of stirring, the reaction was cooled again to -78 °C and phenylacetaldehyde (8 mmol, 961 mg, 890 µL) was added dropwise. After 20 minutes of stirring, the reaction was allowed to warm to room temperature and stirred for an additional 16 hours. The reaction was quenched by the addition of 10 mL deionized water (initially added dropwise), and the product was extracted with petroleum ether (2 x 20 mL). The organic layers were washed with brine, dried with magnesium sulphate, and concentrated under reduced pressure. The product was purified by column chromatography (using pentane as the eluent) and carefully concentrated *via* rotary evaporation to yield a colourless oil (26% yield, 250 mg, 82% d-incorporation)

^{1}H (500 MHz, $CDCl_3$) 7.35-7.16 (ar, m, 5H), 5.92 ($PhCH_2C\textit{H}CD_2$, m, 1H), 3.40 ($PhC\textit{H}_2CHCD_2$, d, 2H, ^{3}J = 6.7 Hz)

$^{13}C\{^{1}H\}$ (126 MHz, $CDCl_3$) 140.2 (C_4), 137.4 (C_6), 128.7 (C_3), 128.6 (C_2), 126.2 (C_1), 40.3 (C_5) (C_7 not observed)

^{2}H (77 MHz, $CDCl_3$) 5.13 (s)

TOF-MS [$M+Na^+$] 143.0840 (theoretical), 143.0824 (observed)

Data concordant with previous literature [10]

5. Extended Optimization Data

CONDITIONS	CATALYST (5 mol%)	CO-CATALYST (10 mol%)	CONVERSION / % (*cis* : *trans*)
16 hr, 60 °C, C_6D_6	None	$H_3N{\cdot}BH_3$	0
16 hr, 60 °C, CD_3CN	(1)	HBpin	0
16 hr, 60 °C, CD_3CN	(1)	$NH_3{\cdot}BH_3$	18 (27 : 1)
16 hr, 30°C, CD_2Cl_2	(1)	HBpin	0
16 hr, 30°C, CD_2Cl_2	(1)	$H_3N{\cdot}BH_3$	0
16 hr, 60°C, CD_2Cl_2	(1)	$H_3N{\cdot}BH_3$	0
16 hr, 60 °C, C_6D_6	(1)	HBcat	0
48 hr, 80 °C, C_6D_6	(1)	HBcat	21 (9.6 : 1)
16 hr, 60 °C, C_6D_6	(1)	$H_2SiMePh$	2 (*not determined*)
48 hr, 80 °C, C_6D_6	(1)	$H_2SiMePh$	15 (4.1 : 1)
16 hr, 60 °C, C_6D_6	(1)	$HSiPh_3$	0

Standard reaction conditions – 600 µL solvent, 0.5 mmol allylbenzene. Conversion and selectivity determined by *in situ* ^{1}H NMR spectroscopy

6. Studies with Linear Alkenes

	Conversion / %				
Substrate	1-Hexene	Trans-2-Hexene	Cis-2-Hexene	Trans-3-Hexene	Cis-3-Hexene
1-Hexene	31	42	10	15	2
Trans-2-Hexene	33	37	12	16	2
Cis-2-Hexene	44	9	33	13	1
Trans-3-Hexene	54	13	15	14	4
Cis-3-Hexene	44	28	7	17	3

Conditions: 600 µL of C_6D_6, 0.5 mmol substrate, 0.025 mmol (5 mol%) catalyst **1**, 0.05 mmol $NH_3{\cdot}BH_3$, 48 hr, 80 °C. Reactions performed in a J-Young Schlenk flask under an Argon atmosphere. Spectroscopic yield and selectivity obtained by *in situ* ^{13}C NMR spectroscopy

7. Characterisation of Products

Products **3A** through **3J** isolated as clear, volatile oils.

3B (E)-1-(2-methylbenzyl)prop-1-ene

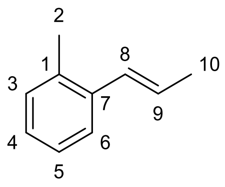

Obtained from standard procedure, 82% Spectroscopic Conversion, 58% Isolated, 38.3 mg

^{1}H (500 MHz, $CDCl_3$) 7.44-7.09 (aromatic, m, 4H), 6.62 ($CH_3PhC\mathit{H}CHCH_3$, dq, 1H, ^3J = 15.6 Hz, ^4J = 1.8 Hz), 6.13 ($CH_3PhCHC\mathit{H}CH_3$, dq, 1H, ^3J = 15.6 Hz, ^3J = 6.6 Hz), 2.36 ($C\mathit{H_3}PhCHCHCH_3$, s, 3H), 1.93 ($CH_3PhCHCHC\mathit{H_3}$, dd, 3H, ^3J = 6.6 Hz, ^4J = 1.8 Hz)

^{13}C{^{1}H} (126 MHz, $CDCl_3$) 137.2 (C_7), 134.9 (C_1), 130.9 (C_3), 130.2 (C_9), 129.0 (C_6), 127.1 (C_5), 126.8 (C_4) 123.6 (C_8), 55.3 (C_2), 18.9 (C_{10})

TOF-MS (EI+) [O] 148.0888 (calc) 148.0889 (obs)

Data concordant with previous literature [11]

3C (*E*)-1-(3-methylbenzyl)prop-1-ene

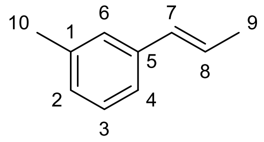

Obtained from standard procedure, 92% Spectroscopic Conversion, 65% Isolated, 43.0 mg

^{1}H (500 MHz, CDCl$_3$) 7.10-7.25 (aromatic, m, 4H), 6.39 (CH$_3$PhC*H*CHCH$_3$, dq, 1H, ^3J = 15.7 Hz, ^4J = 1.7 Hz), 6.24 (CH$_3$PhCHC*H*CH$_3$, dq, 1H, ^3J = 15.7 Hz, ^3J = 6.6 Hz), 2.35 (C*H$_3$*PhCHCHCH$_3$, s, 3H), 1.89 (CH$_3$PhCHCHCH$_3$, dd, 3H, ^{3}J = 6.6 Hz, ^{4}J = 1.7 Hz)

^{13}C{^{1}H} (126 MHz, CDCl$_3$) 138.0 (C$_5$), 137.9 (C$_1$), 131.1 (C$_7$), 128.3 (C$_6$), 127.5 (C$_3$), 126.6 (C$_2$), 125.5 (C$_8$), 122.9 (C$_4$), 21.5 (C$_{10}$), 18.5 (C$_9$)

TOF-MS (EI+) [M] 132.0939 (calc) 132.0933 (obs)

Data concordant with previous literature [12]

3D (*E*)-1-(4-methylbenzyl)prop-1-ene

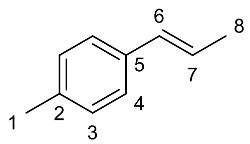

Obtained from standard procedure, 98% Spectroscopic Conversion, 72% Isolated, 47.6 mg

^{1}H (300 MHz, CDCl$_3$) 7.01-7.18 (ar, m, 4H), 6.30 (CH$_3$PhC*H*CHCH$_3$, ^3J = 15.7 Hz, ^{4}J = 1.6 Hz, 1H), 6.11 (CH$_3$PhCHC*H*CH$_3$, dq, ^3J = 15.7 Hz, ^3J = 6.5 Hz, 1H), 2.25 (C*H$_3$*PhCHCHCH$_3$, s, 3H), 1.79 (CH$_3$PhCHCHC*H$_3$*, dd, ^3J = 6.5 Hz, ^4J = 1.6 Hz, 3H),

^{13}C{^{1}H} (126 MHz, CDCl$_3$) – 136.4 (C$_2$), 135.2 (C$_5$), 130.8 (C$_6$), 129.2 (C$_3$), 125.7 (C$_4$), 124.6 (C$_7$), 21.1 (C$_1$), 18.4 (C$_8$)

TOF-MS (ASAP+) [M-H] 131.0861 (calc) 131.0851 (obs)

Data concordant with previous literature [11]

3E (E)-1-(2-methoxybenzyl)prop-1-ene

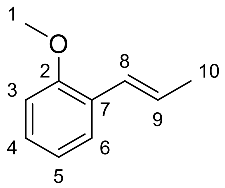

Obtained from standard procedure, 92% spectroscopic yield, 69% isolated product, 51.1 mg

^{1}H (500 MHz, CDCl$_3$) 7.42-6.83 (ar, m, 4H), 6.73 (CH$_3$OPhC*H*CHCH$_3$, dq, ^3J = 15.9 Hz, ^4J = 1.8 Hz, 1H), 6.24 (CH$_3$OPhCHC*H*CH$_3$, dq, ^3J = 15.9 Hz, ^3J = 6.6 Hz, 1H), 3.85 (C*H$_3$*OPhCHCHCH$_3$, s, 3H), 1.91 (CH$_3$OPhCHCHC*H$_3$*, dd, ^3J = 6.6 Hz, ^4J = 1.8 Hz, 3H)

^{13}C{^{1}H} (126 MHz, CDCl$_3$) 156.2 (C$_2$), 127.8 (C$_4$), 127.0 (C$_7$), 126.5 (C$_9$), 126.4 (C$_6$), 125.6 (C$_8$), 120.6 (C$_5$), 110.7 (C$_3$), 55.4 (C$_1$), 18.9 (C$_{10}$)

TOF-MS (ASAP+) [M+O+H] 165.0916 (calc) 165.0909+ (obs)

Data concordant with previous literature [11]

3F (E)-1-(4-methoxybenzyl)prop-1-ene ((*E*)-Anethole)

Obtained from standard procedure, 98% spectroscopic yield, 62% isolated, 45.9 mg

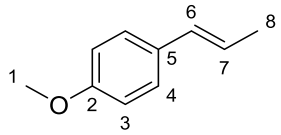

^{1}H (500 MHz, $CDCl_3$) 7.11 (*H*C_4, d, ^{3}J = 8.6 Hz), 6.84 (*H*C_3, d, ^{3}J = 8.6 Hz), 6.42 (CH_3OPhC*H*CHCH_3, dq, ^{3}J = 15.8 Hz, ^{4}J = 1.7 Hz, 1H), 6.17 (CH_3OPhCHC*H*CH_3, dq, ^{3}J = 15.8 Hz, ^{3}J = 6.6 Hz, 1H) 3.86 (C*H₃*OPhCHCHCH_3, s, 3H), 1.93 (CH_3OPhCHCHCH_3, dd, ^{3}J = 6.6 Hz, ^{4}J = 1.7 Hz, 3H)

^{13}C{^{1}H} (126 MHz, $CDCl_3$) 158.6 (C_2), 130.8 (C_5), 130.4 (C_6), 126.9 (C_4), 123.5 (C_7), 113.9 (C_3), 55.2 (C_1), 18.4 (C_8)

TOF-MS (ASAP+) [M+H] 149.0966 (calc) 149.1058 (obs)

Minor amounts of the hydrogenated product **2F*** were also observed in the ^{1}H and ^{13}C NMR (18% spectroscopic, 15% isolated)

Data concordant with previous literature [13]

3F* 1-methoxy-4-propylbenzene

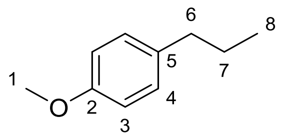

^{1}H (500 MHz, $CDCl_3$)7.11 (*ortho* to allyl group, m, 2H), 6.84 (*ortho* to methoxy group, m, 2H), 3.80 (CH_3OPhCH_2CH_2CH_3, s, 3H), 2.54 (CH_3OPhCH_2CH_2CH_3, m, 2H), 1.62 (CH_3OPhCH_2CH_2CH_3, m, 2H), 0.95 (CH_3OPhCH_2CH_2CH_3, t, ^{3}J = 7.3 Hz, 3H)

^{13}C (126 MHz, $CDCl_3$) – 157.6 (C_2), 134.8 (C_5), 129.3 (C_4), 113.6 (C_3), 55.3 (C_1), 37.2 (C_6), 24.9 (C_7), 13.7 (C_8)

Data concordant with previous literature [14]

3G (E)-1-(4-Fluorophenyl)prop-1-ene

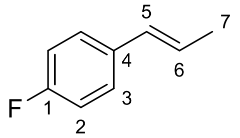

Obtained from modified procedure, 70% spectroscopic yield, 41% isolated product, 28.0 mg

^{1}H (500 MHz, $CDCl_3$) 7.25-7.31 (ar, m, 2H), 7.01-6.95 (ar, m, 2H), 6.37 (FPhC*H*CHCH$_3$, dq, ^3J = 15.7 Hz, ^4J = 1.8 Hz, 1H), 6.15 (FPhCHC*H*CH$_3$, dq, ^3J = 15.7 Hz, ^3J = 6.6 Hz, 1H), 1.88 (FPhCHCHC*H*$_3$, dd, ^3J = 6.6 Hz, ^4J = 1.8 Hz, 3H)

^{13}C{^{1}H} (126 MHz, $CDCl_3$) 161.8 (C_1, d, ^1J = 245.5 Hz) , 134.1 (C_4, d, ^4J = 3.3 Hz), 129.8 (C_6), 127.2 (C_3, d, ^3J = 7.7 Hz), 125.4 (C_5, d, ^5J = 1.9 Hz), 115.2 (C_2, d, ^2J = 21.4 Hz) 18.4 (C_7)

^{19}F (470 MHz, $CDCl_3$) -116.0 (s)

TOF-MS (ASAP+) [M-H] 135.0610 (calc) 135.0607 (obs)

The minor product (Z)-1-(4-Fluorophenyl)prop-1-ene was also observed in the ^{19}F NMR and ^{1}H NMR

^{1}H (500 MHz, $CDCl_3$) 7.20-6.92 (m, 4H) 5.95 (m, 1H), 5.78 (dq ^3J = 11.5 Hz, ^4J = 7.1 Hz, 1H), 1.62 (m, 3H)

^{19}F (470 MHz, $CDCl_3$) -118.2 (s)

Data concordant with previous literature [11]

3H (E)-1-(4-Trifluorophenyl)prop-1-ene

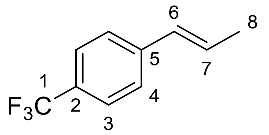

Obtained from modified procedure, 58% spectroscopic yield, 39% isolated product, 36.3 mg

^{1}H (500 MHz, $CDCl_3$), 7.62-7.36 (ar, m, 4H), 6.43 (CF_3PhC*H*CHCH$_3$, dq, ^3J = 15.8 Hz, ^4J = 1.5 Hz, 1H) 6.35 (CF_3PhCHC*H*CH$_3$, dq, ^3J = 15.8 Hz, ^3J = 6.4 Hz, 1H), 1.92 (CF_3PhCHCHC*H*$_3$, dd, ^3J = 6.4 Hz, ^4J = 1.4 Hz, 3H)

^{13}C{^{1}H} (126 MHz, $CDCl_3$) 141.3 (C_2), 129.9 (C_5), 128.9 (C_6), 128.6 (C_7), 125.9 (C_4), 125.4 (C_3), 123.2 (C_1) 18.5 (C_8)

^{19}F (470 MHz, $CDCl_3$) -62.4 (s)

TOF-MS (ASAP+) [M] 186.0656 (calc) 186.0653 (obs) [M-H] 185.0578 (calc) 185.0575 (obs)

The minor product (Z)-1-(4-Trifluorophenyl)prop-1-ene was also observed ^{19}F NMR and ^{1}H NMR

^{1}H (500 MHz, $CDCl_3$) 7.60-7.25 (m, 4H), 6.00-5.87 (m, 2H), 1.53 (s, 3H)

^{19}F (470 MHz, $CDCl_3$) -62.3 (s)

Data concordant with previous literature [15]

3I Pinene

Pinene conversion determined by comparison of ^{1}H NMR peaks of starting material and product as shown below

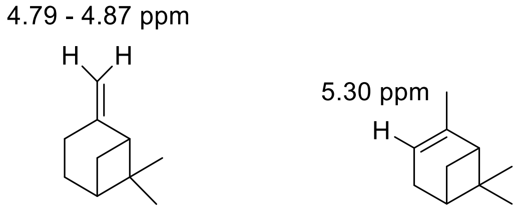

3J Naphthalene,1,2,3,5,6,7,8,8a-octahydro-1,8a-dimethyl-7-(1-methylethylidene)- (1R, 8aS)

Conversion from valencene was determined by comparison of ^{1}H NMR peaks of starting material and product as shown below

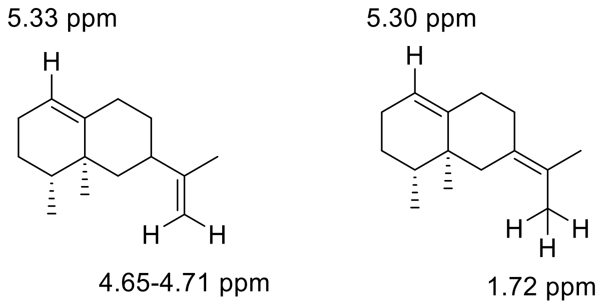

3K Isosafrole

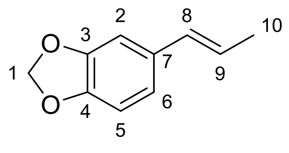

Obtained from standard procedure, 97% spectroscopic yield, 65% isolated, 52.7 mg

^{1}H (500 MHz, $CDCl_3$) 6.89 - 6.74 (m, 3H) 6.32 (PhC*H*CHCH$_3$, ^{3}J = 14.4 Hz, ^{4}J = 1.2 Hz, 1H), 6.08 (PhCHC*H*CH$_3$, dq, ^{3}J = 14.4 Hz, ^{3}J = 6.6 Hz, 1H), 5.92 (OC*H$_2$*O, d, ^{4}J = 1.2 Hz, 2H), 1.86 (PhCHCHC*H$_3$*, dd, ^{3}J = 7.5 Hz, ^{4}J = 1.2 Hz, 3H)

$^{13}C\{^{1}H\}$ (126 MHz, $CDCl_3$) 147.8 (C_3), 145.7 (C_4), 132.9 (C_7), 130.9 (C_8), 124.3 (C_9), 120.4 (C_6), 108.3 (C_5), 105.7 (C_2), 101.0 (C_1), 18.7 (C_{10})

Data concordant with previous literature [16]

3K* Piperonal (Prepared from isosafrole *via* ozonolysis)

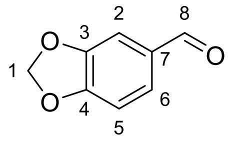

Isolated as white solid (81 mg, 54% yield)

^{1}H (500 MHz, $CDCl_3$) 9.82 (PhC*HO*, s, 1H) 7.42 (*H*C$_6$, dd, ^{3}J = 7.9 Hz, ^{4}J = 1.6 Hz, 1H), 7.34 (*H*C$_2$, d, ^{4}J = 1.6 Hz, 1H), 6.94 (*H*C$_5$, d, ^{3}J = 7.9 Hz), 6.08 (OC*H$_2$*O, s, 2H)

$^{13}C\{^{1}H\}$ (126 MHz, $CDCl_3$) 190.4 (C_8), 153.3 (C_4), 148.9 (C_3), 132.1 (C_7), 128.8 (C_6), 108.5 (C_5), 107.1 (C_2), 102.3 (C_1)

TOF-MS [M+H] 151.0389 (calc) 151.0387 (obs)

Data concordant with previous literature [17]

8. Deuteration Experiments & Characterisation

D1

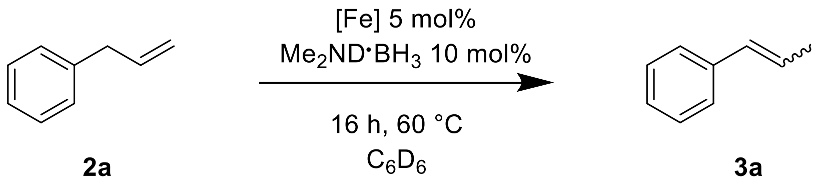

No deuterium incorporation on product

Trans/E product ●

6.31 (PhC*H*CHCH3, ∫ = 1.00), 6.15 (PhCHC*H*CH3, 1.00), 1.79 (PhCHCHC*H$_3$*, 3.02)

Expected integrals for no deuterium incorporation : 1.00, 1.00, 3.00

Result 1.00, 1.00, 3.02

Cis/Z product ●

6.36 (PhC*H*CHCH3, 0.18), 5.70 (PhCHC*H*CH3, 0.19), 1.82 (PhCHCHC*H₃*, 0.59)

Expected integrals for no deuterium incorporation 0.19, 0.19, 0.57

Result 0.18, 0.19, 0.59

Starting material ●

5.88 ($PhCH_2C$*H*CH2, 0.24), 4.98 ($PhCH_2CHC$*H_2*, 0.49), 3.30 (PhC*H_2*$CHCH_2$, 0.51)

Hydrogenated side-product observed – alkyl peaks marked with ● (^{1}H 2.50, 1.44, 0.85 ppm)

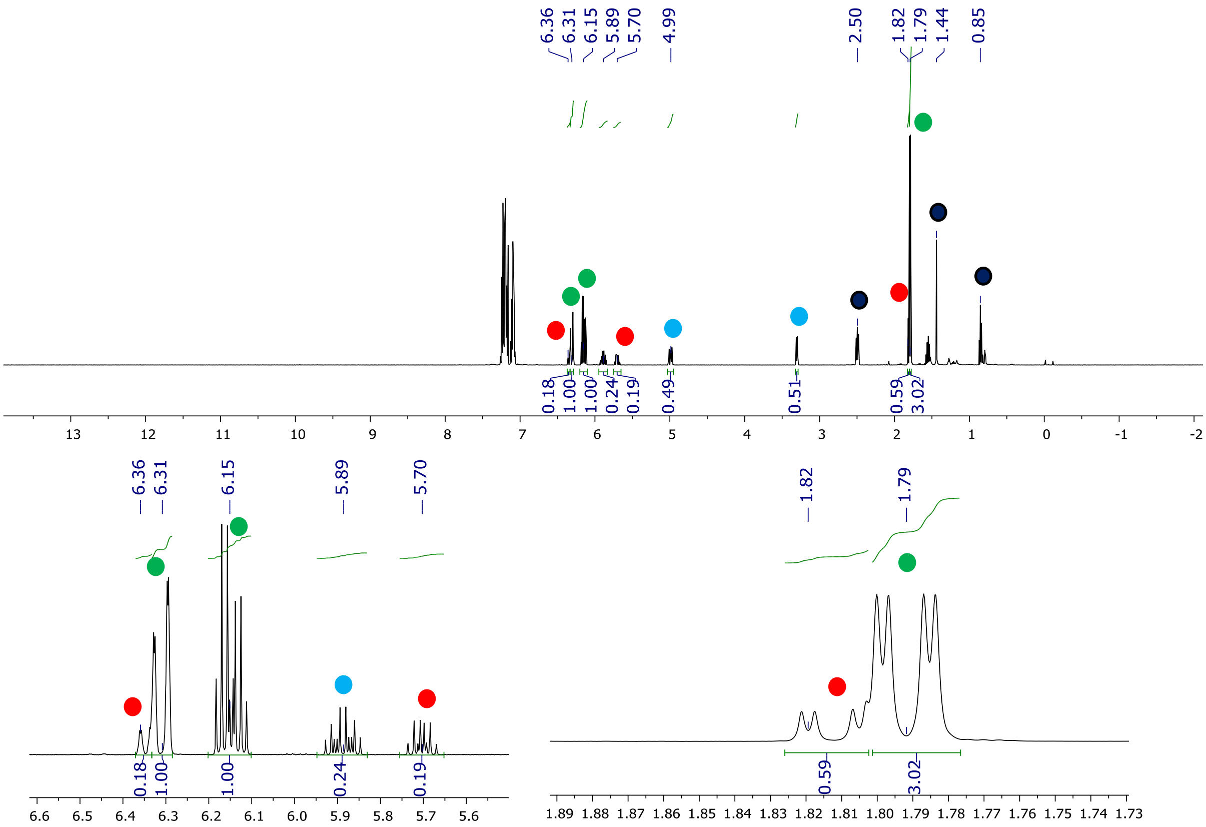

D2

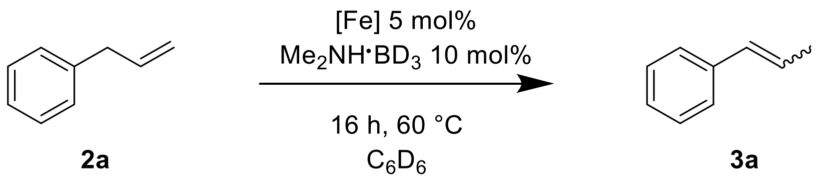

No deuterium incorporation on product

Trans/E product ●

6.31 (PhC*H*CHCH3, ∫ = 1.00), 6.15 (PhCHC*H*CH3, 1.00), 1.79 (PhCHCHC*H_3*, 2.99)

Expected integrals for no deuterium incorporation : 1.00, 1.00, 3.00

Result 1.00, 1.00, 2.99

Cis/Z product ●

6.36 (PhC*H*CHCH3, 0.19), 5.70 (PhCHC*H*CH3, 0.19), 1.82 (PhCHCHCH_3, 0.56)

Expected integrals for no deuterium incorporation 0.19, 0.19, 0.57

Result 0.19, 0.19, 0.57

Starting material ●

5.88 (PhCH_2C*H*CH2, 1.01), 4.98 (PhCH_2CHCH_2, 2.09), 3.30 (PhCH_2CH$_2$, 2.16)

Hydrogenated side-product observed – alkyl peaks marked with ● (^{1}H 2.49, 1.44, 0.89 ppm)

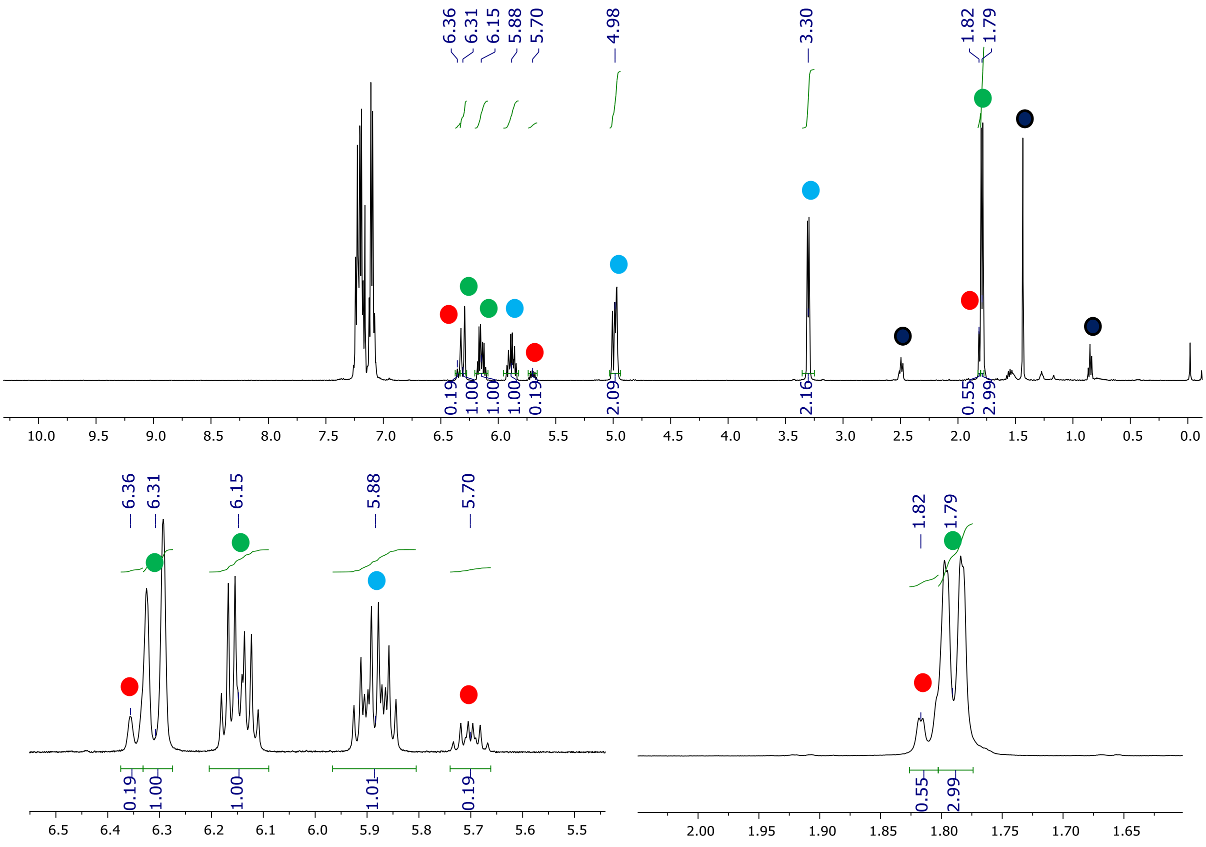

D3

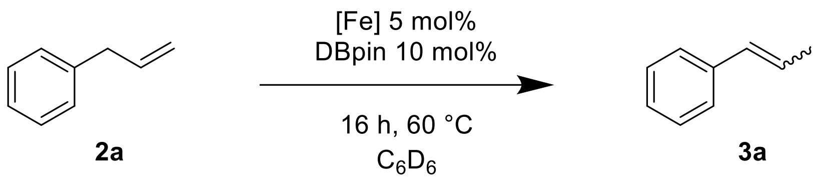

No deuterium incorporation

Trans/E product ●

6.31 (PhC*H*CHCH3, ∫ = 1.00), 6.15 (PhCHC*H*CH3, 1.00), 1.79 (PhCHCHCH_3, 3.08)

Expected integrals for no deuterium incorporation : 1.00, 1.00, 3.00

Result 1.00, 1.00, 3.08

Cis/Z product ●

6.36 (PhC*H*CHCH3, 0.13), 5.70 (PhCHC*H*CH3, 0.15), 1.82 (PhCHCHCH_3, 0.45)

Expected integrals for no deuterium incorporation 0.14, 0.14, 0.42

Result 0.13, 0.15, 0.42

Starting material ●

5.91 (PhCH_2C*H*CH2, 0.06), 5.01 (PhCH_2CHCH_2, 0.13), 3.30 (PhCH_2CHCH_2, 0.13)

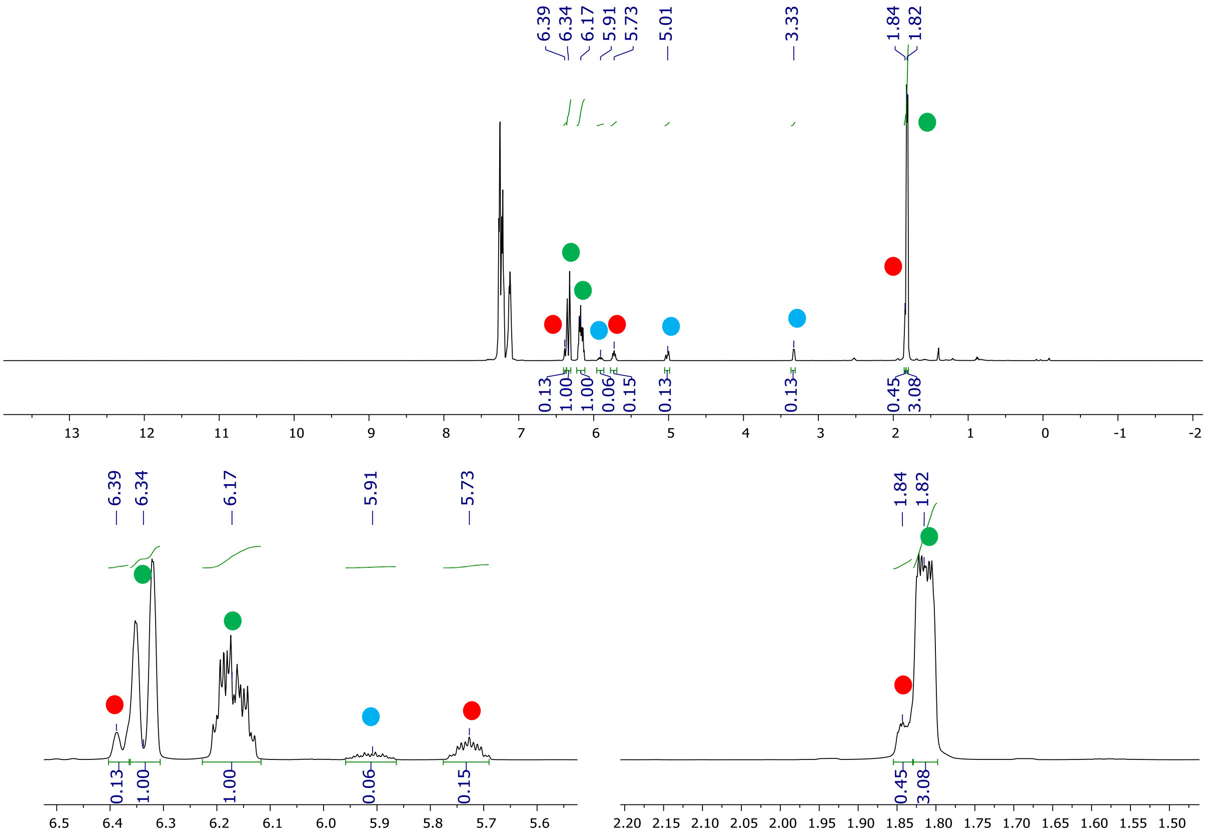

D4

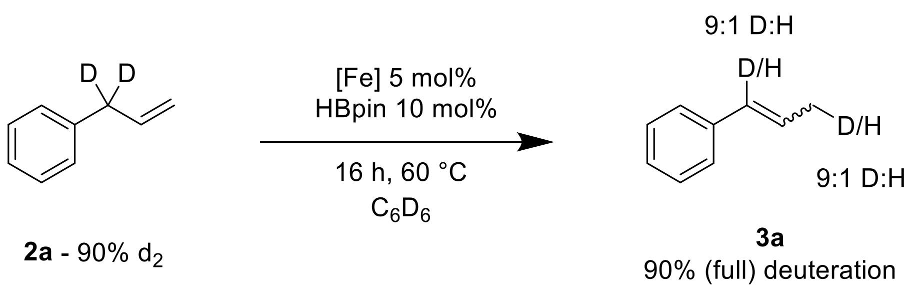

2a - 90% d_2

3a
90% (full) deuteration

The reaction was quenched at 40% to observe if any there was any variation in H/D exchange over the course of the reaction. The reaction was then repeated and run to full (90%) conversion. In both cases NMR data is consistent will full D retention on substrate. H/D NMR spectra of both cases are shown

At 40% conversion

Trans/E product ●

6.32 (PhC*H*CHCH3, residual protic product, 0.15), 6.17 (PhCHC*H*CH3, 1.00), 1.81 (PhCHCHCH_3, 2.22)

Expected integrals for full deuteration of 90%-d_2 substrate 0.1, 1.00, 2.10

Result 0.15, 1.00, 2.22

Cis/Z product ●

6.43 (PhC*H*CHCH3, residual protic product, 0.03), 5.62 (PhCHC*H*CH3, 0.13), 1.84 (PhCHCHCH_3, 0.28)

Expected integrals for full deuteration of 90%-d_2 substrate 0.02, 0.13, 0.27

Result 0.03, 0.13, 0.28

Starting material ●

5.90 (PhCH_2C*H*CH2, 1.98), 5.01 (PhCH_2CHCH_2, 3.63), 3.32 (PhCH_2CHCH_2, 0.43, residual protic starting material)

At 90% conversion

Trans/E product ●

6.32 (PhC*H*CHCH3, residual protic product, 0.21), 6.17 (PhCHC*H*CH3, 1.00), 1.81 (PhCHCHCH_3, 2.21)

Expected integrals for full deuteration of 90%-d_2 substrate 0.10, 1.00, 2.10

Result : 0.21, 1.00, 2.21

Cis/Z product ●

6.43 (PhC*H*CHCH3, residual protic product, 0.07), 5.62 (PhCHC*H*CH3, 0.33), 1.84 (PhCHCHCH_3, 0.44)

Expected integrals for full deuteration of 90%-d_2 substrate 0.06, 0.33, 0.44

Starting material ●

Small amounts of starting material remain at 5.91, 5.02, and protic residue at 3.42 ppm respectively

40% Conversion

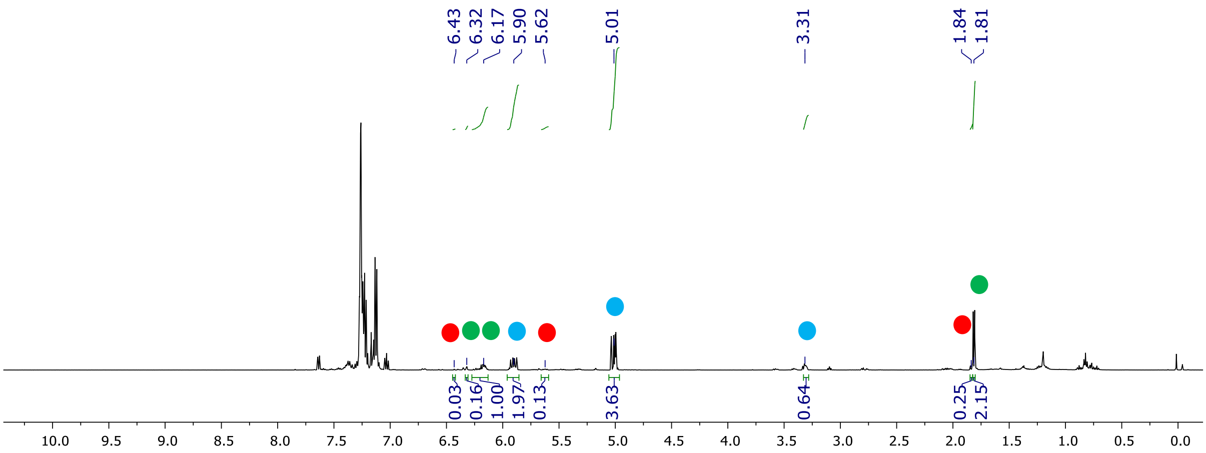

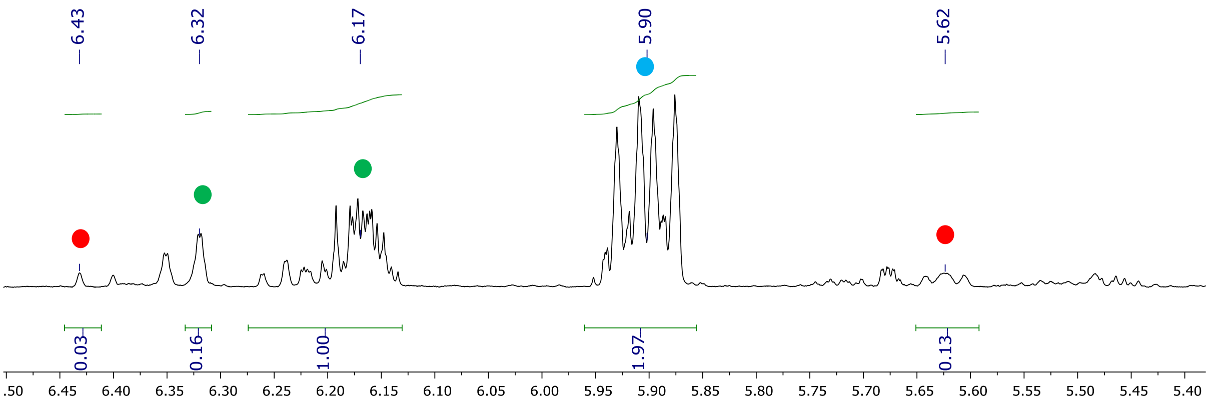

90% Conversion (some side-products also isolated)

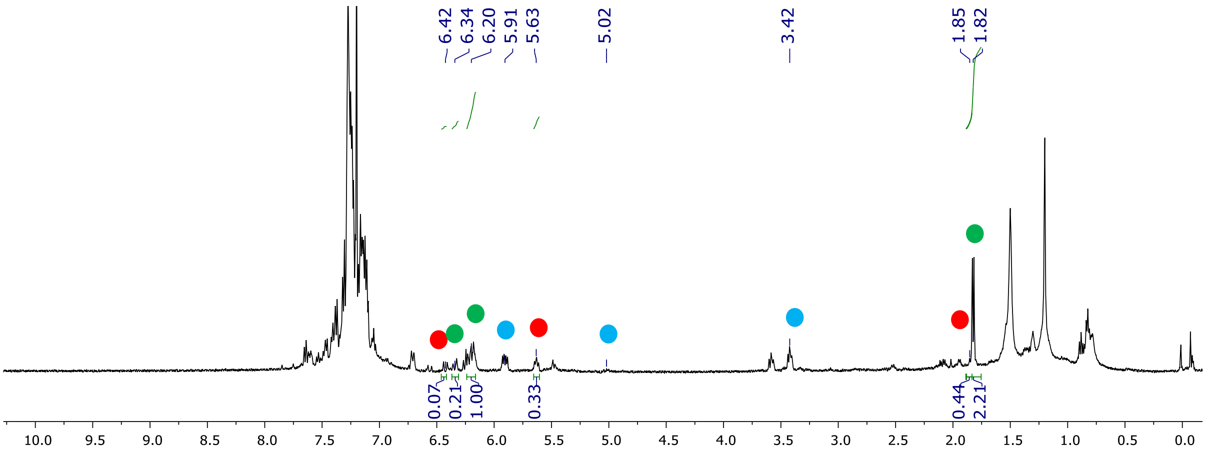

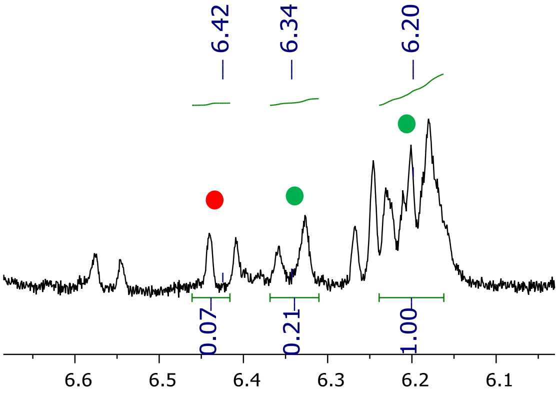

D5

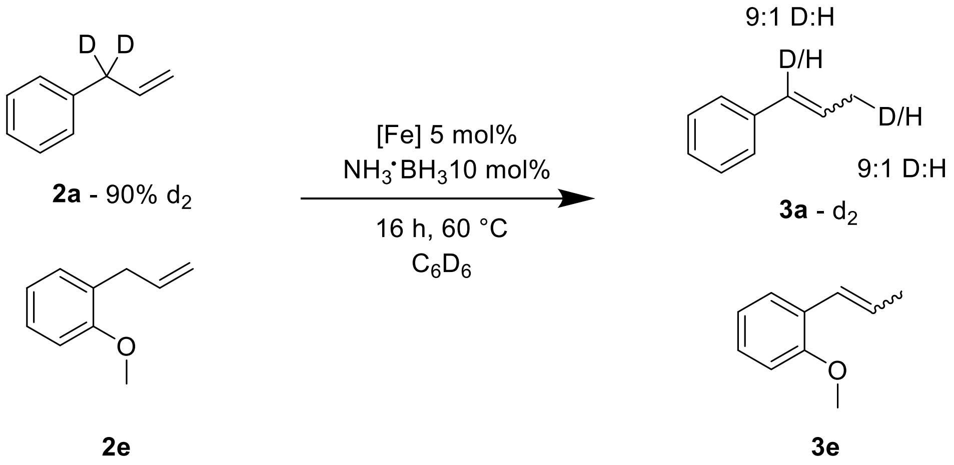

No D incorporation on 2-methoxy substrate

The 2-methoxy products were carefully isolated from the reaction mixture through a silica gel plug (pentane as solvent)

Trans/E product ●

6.72 (CH_3OPhC*H*CHCH3, 1.00), 6.24 (CH_3OPhCHC*H*CH3, 1.02), 2.45 (C*H*$_3$OPhCHCHC*H*$_3$, 3.05), 1.79 (CH_3OPhCHCHC*H*$_3$, 3.01)

Expected integrals for no deuterium incorporation : 1.00, 1.00, 3.00, 3.00

Result 1.00, 1.02, 3.05, 3.01

Cis/Z product ●

6.59 (CH_3OPhC*H*CHCH3, 0.08), 5.96 (CH_3OPhCHC*H*CH3, 0.09), 1.88 (CH_3OPhCHCHC*H*$_3$, 0.24), 1.88 (CH_3OPhCHCHC*H*$_3$, 0.26)

Expected integrals for no deuterium incorporation 0.08, 0.08, 0.24, 0.24

Result 0.08, 0.09, 0.24, 0.24

The hydrogenated side-product was also isolated – alkyl peaks marked with ● (^{1}H 2.73, 1.73, 1.12 ppm)

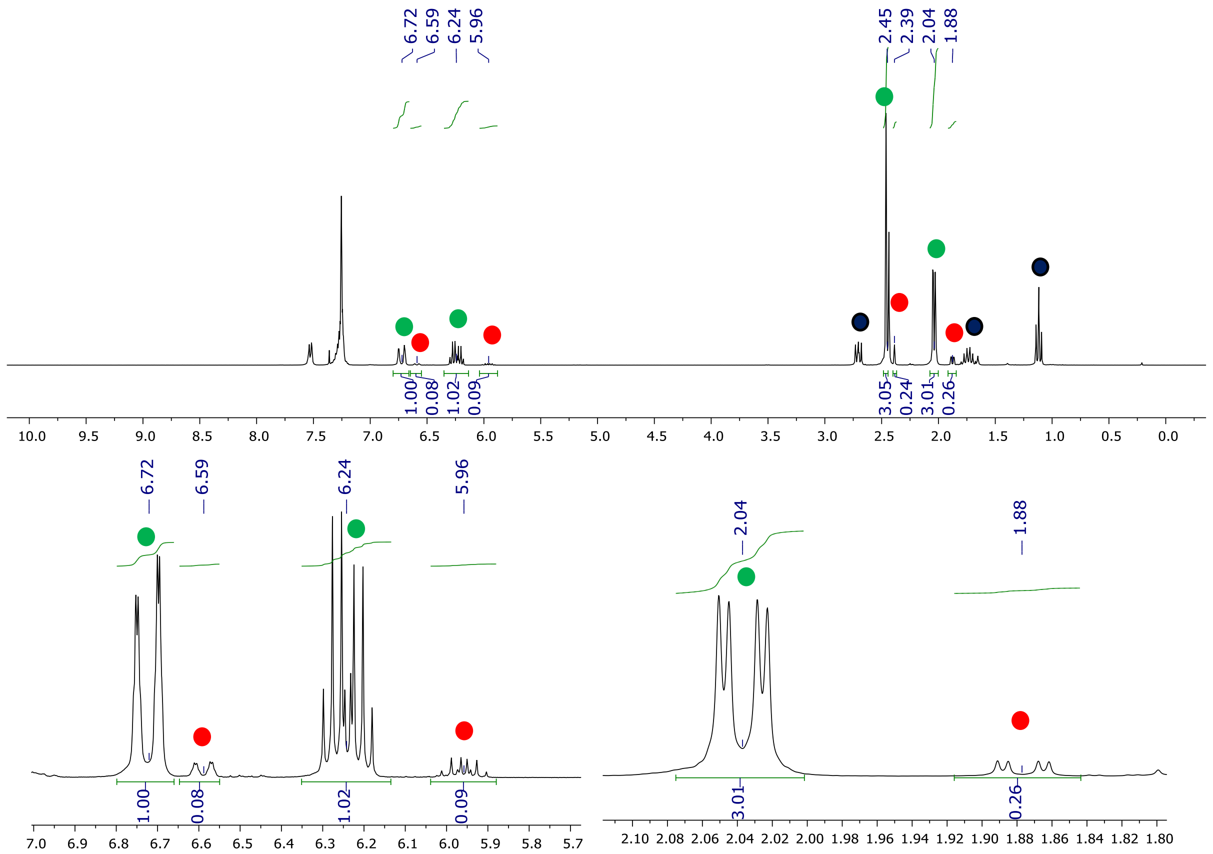

9. Electrochemistry Experimental Data

Cyclic Voltammetry experiments were performed under an argon atmosphere using standard Schlenk techniques and a custom built three-necked Schlenk flask. To 25 mL of dry THF was added the required precatalyst (37 mg, 5 mM concentration) and tetrabutylammonium hexafluorophosphate (1.16 g, 0.1 M concentration). A standard three electrode setup was used (glassy carbon working electrode, platinum counter electrode and silver pseudo-reference electrode). Voltammograms were referenced to Fc/Fc^{+} oxidation potential by addition of 1 mg Ferrocene to the solution.

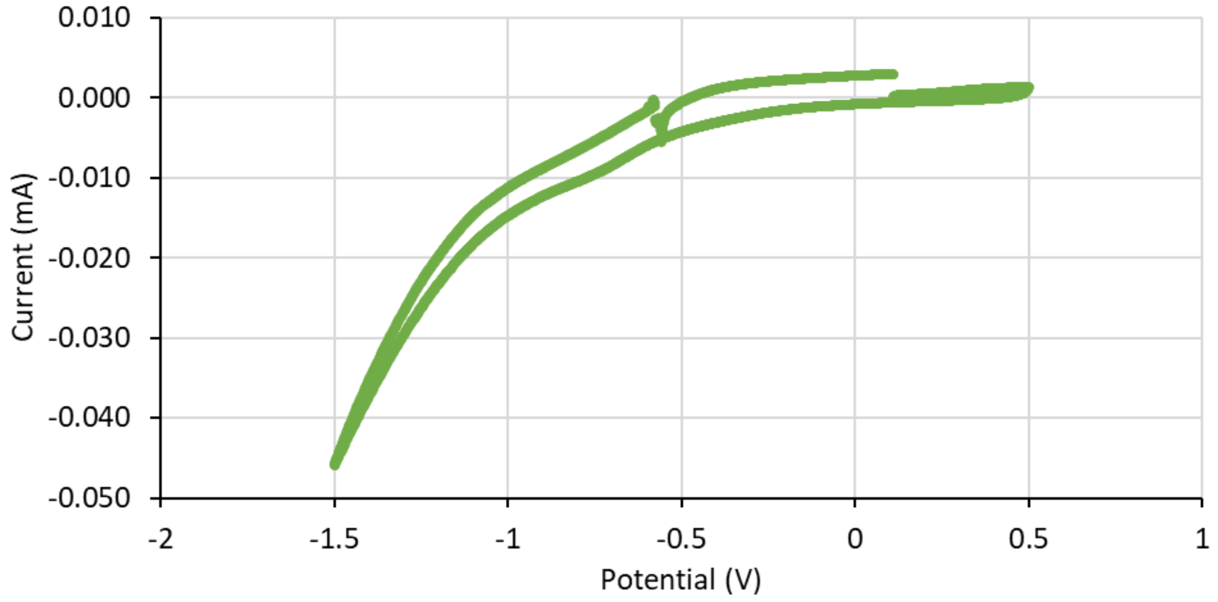

Fig S1 0.1 M tBu_4NPF_6 in 25 mL THF only (100 mVs^{-1}, not referenced)

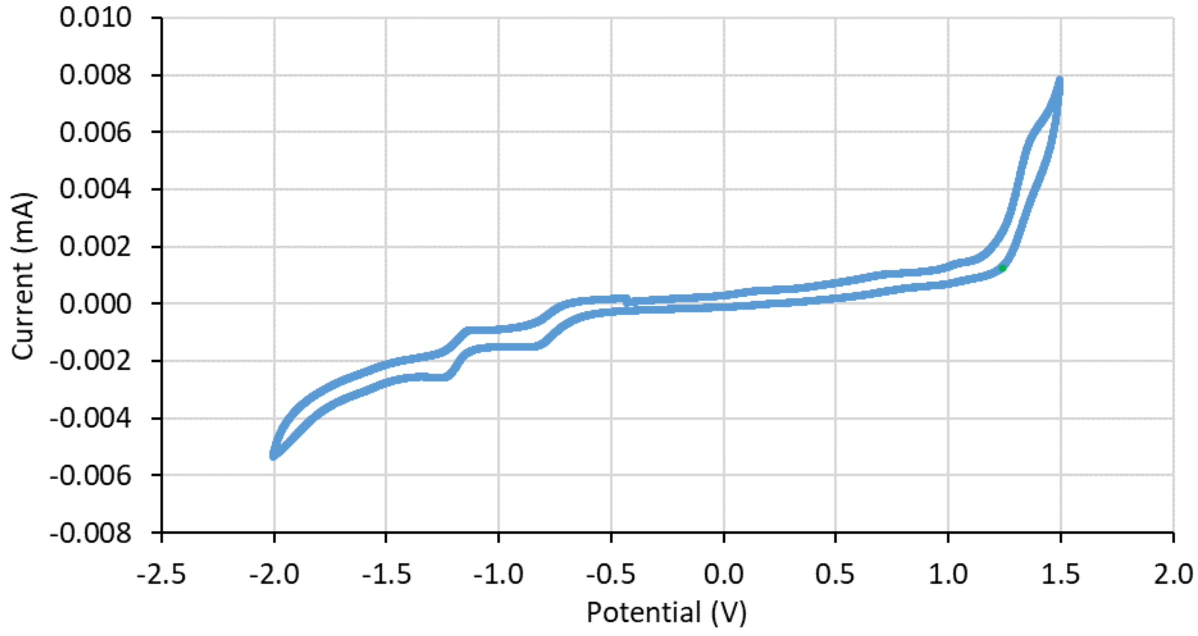

Fig S2 5mM **1** + 0.1 M tBu_4NPF_6 in 25 mL THF (Wide Sweep, 10 mVs^{-1}, not referenced)

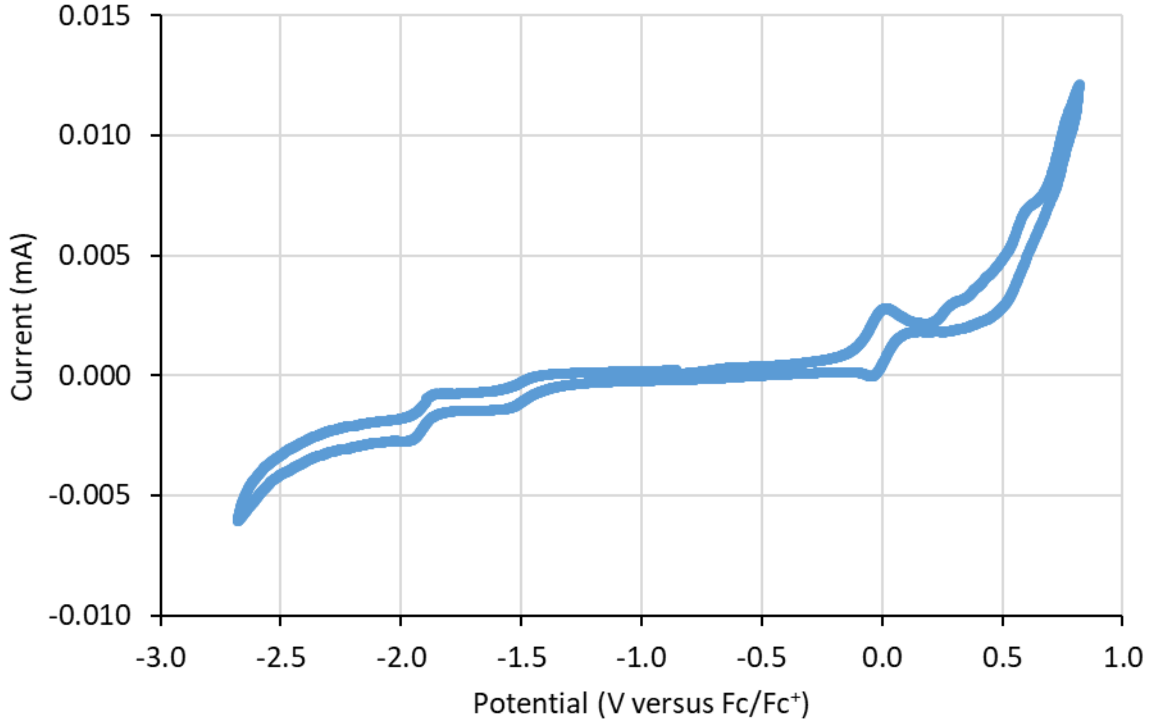

Fig S3 5mM **1** + 0.1 M tBu_4NPF_6 + 1 mg Fc in 25 mL THF (Wide Sweep, 10 mVs^{-1}, referenced to Fc/Fc^+)

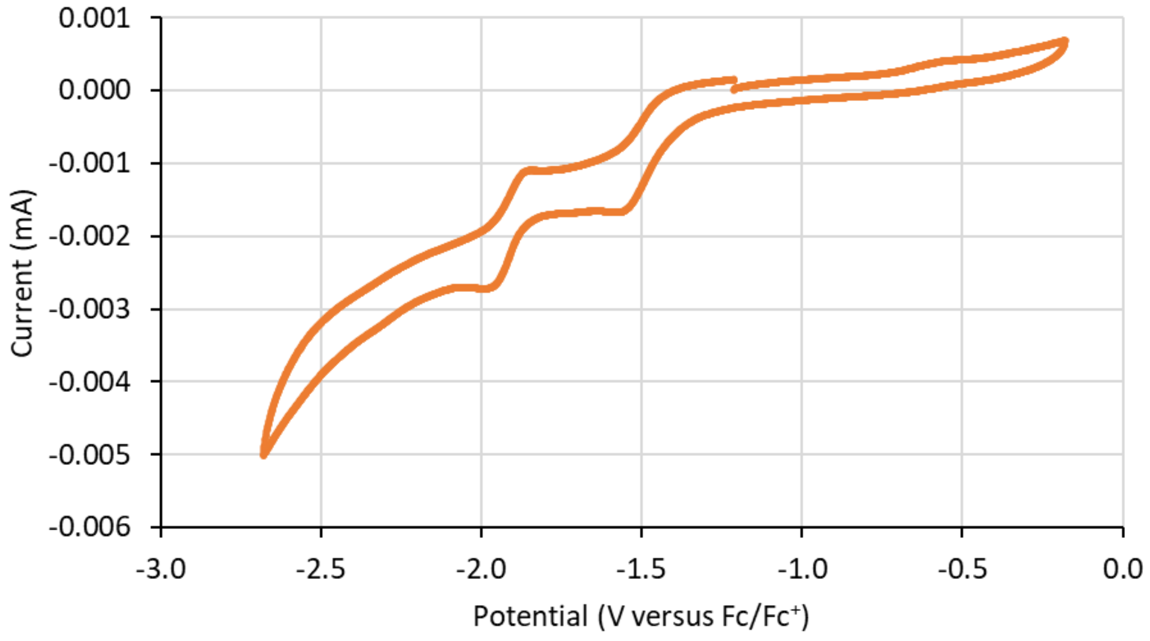

Fig S4 5mM **1** + 0.1 M tBu_4NPF_6 in 25 mL THF (select region only, 10 mVs^{-1})

10. NMR Spectra

10.1 Boranes

TMP·BH_3

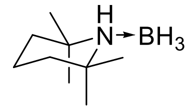

^{1}H

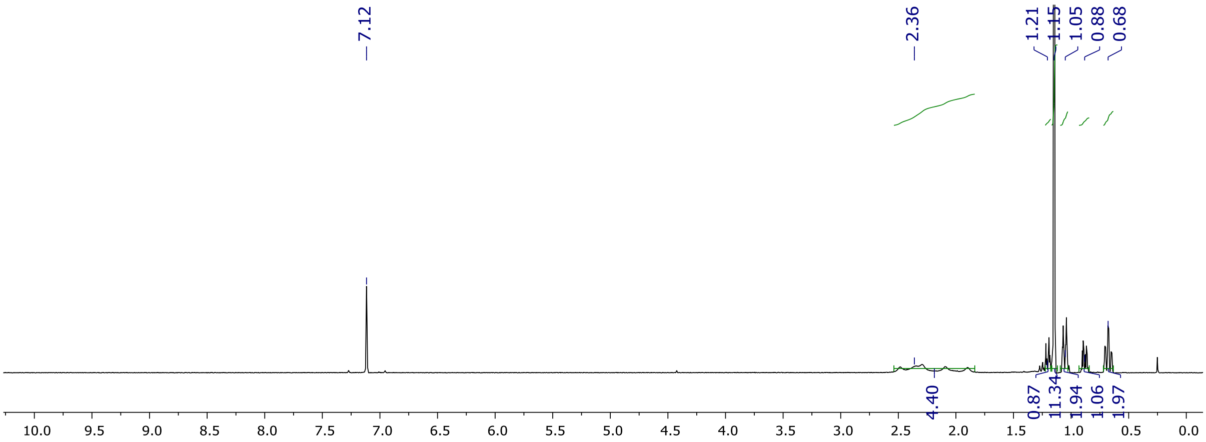

$^{13}C\{^{1}H\}$

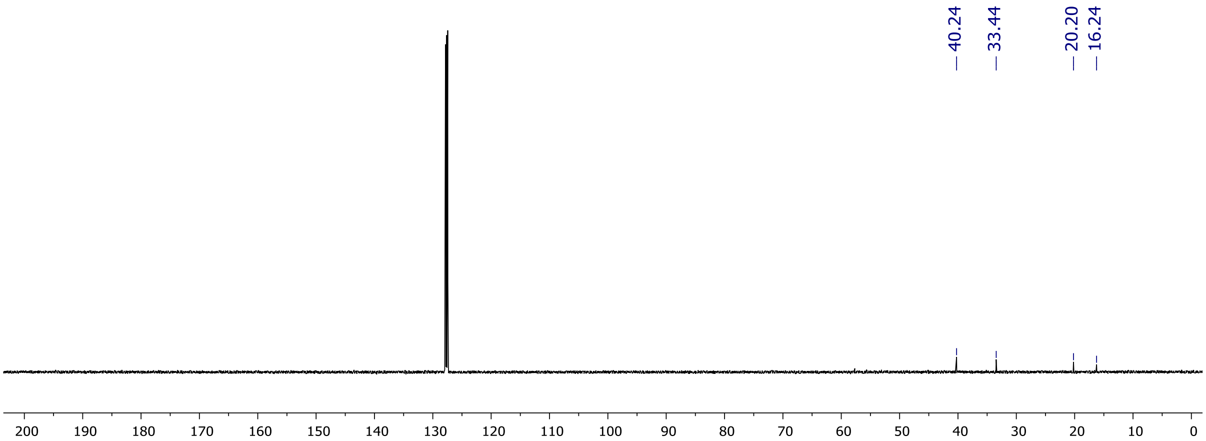

$^{11}B\{^{1}H\}$

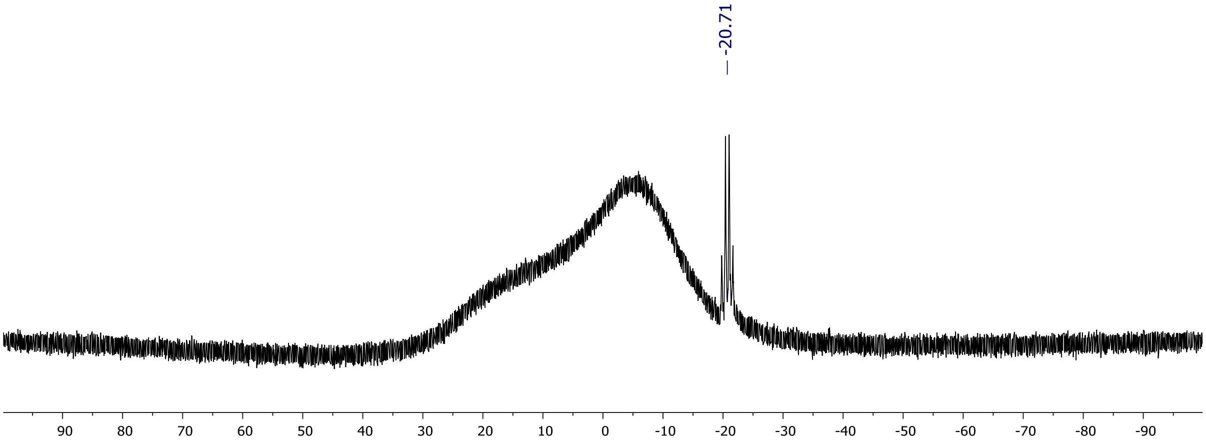

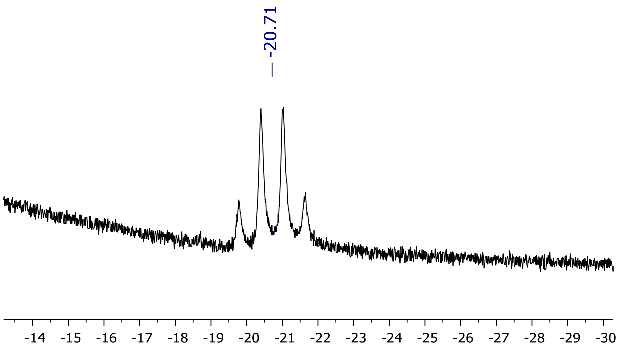

10.2 Deuterated Substrates

Methyltriphenylphosphonium iodide-d$_3$ (CD_3PPh_3I)

^1H

Some residual THF can be observed (3.76 & 1.85 ppm respectively)

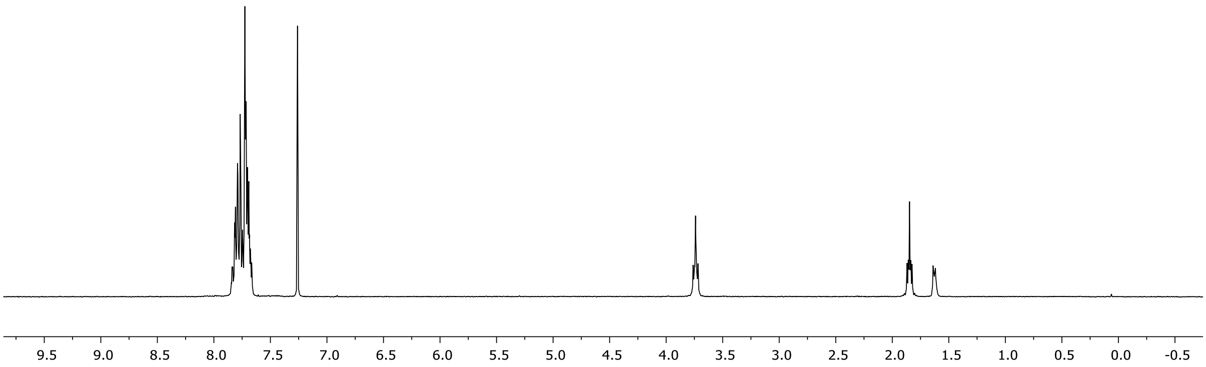

c

$^{13}C\{^{1}H\}$

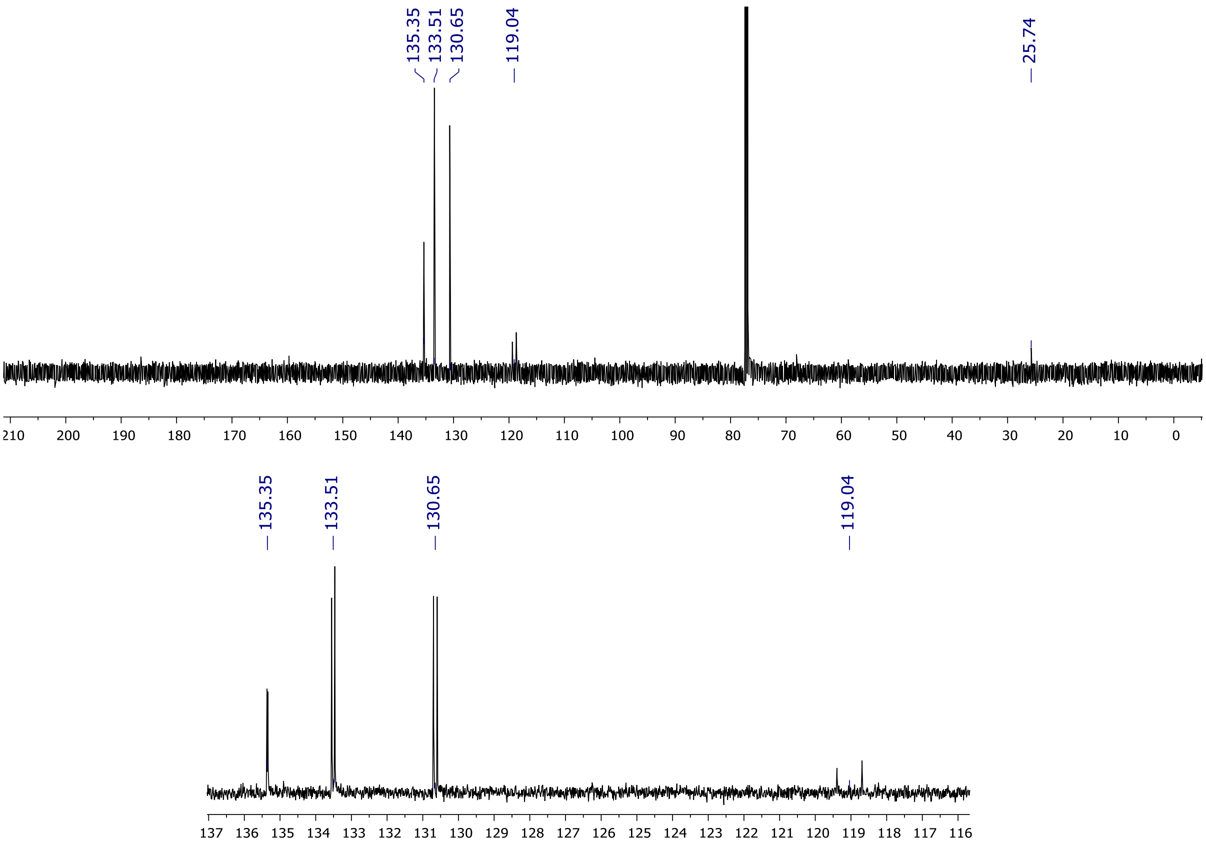

^{2}H

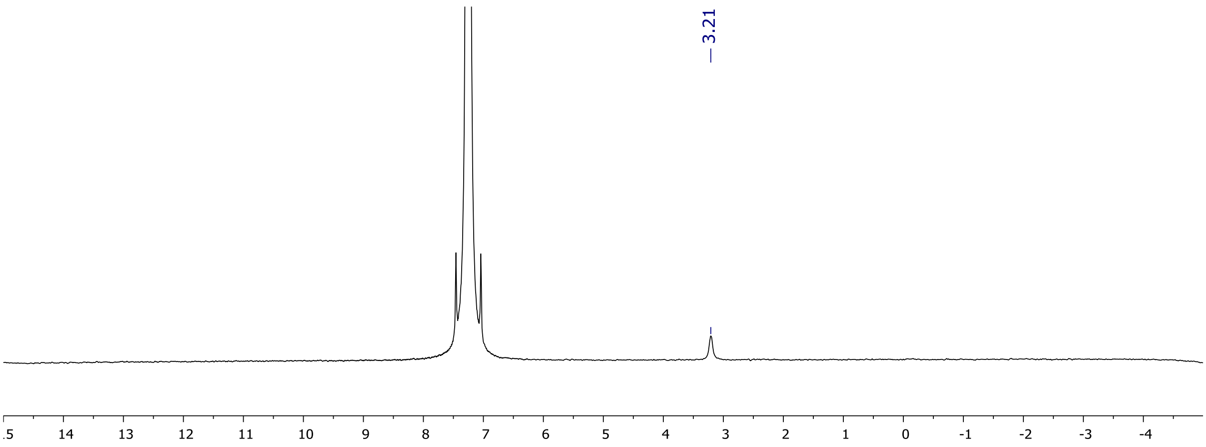

$^{31}P\{^1H\}$

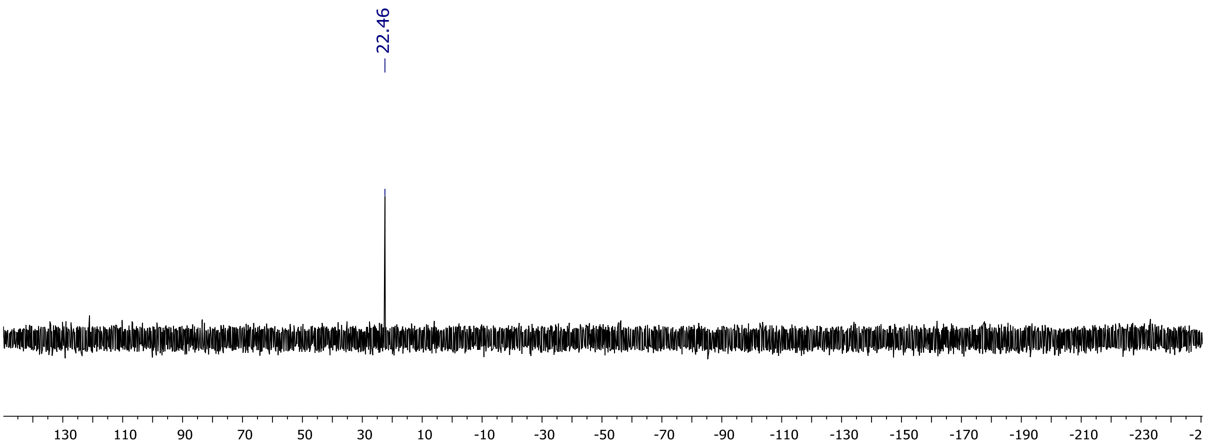

2,2-d$_2$-phenylacetic acid (PhCD_2COOH)

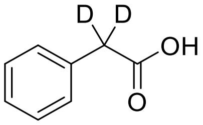

^1H

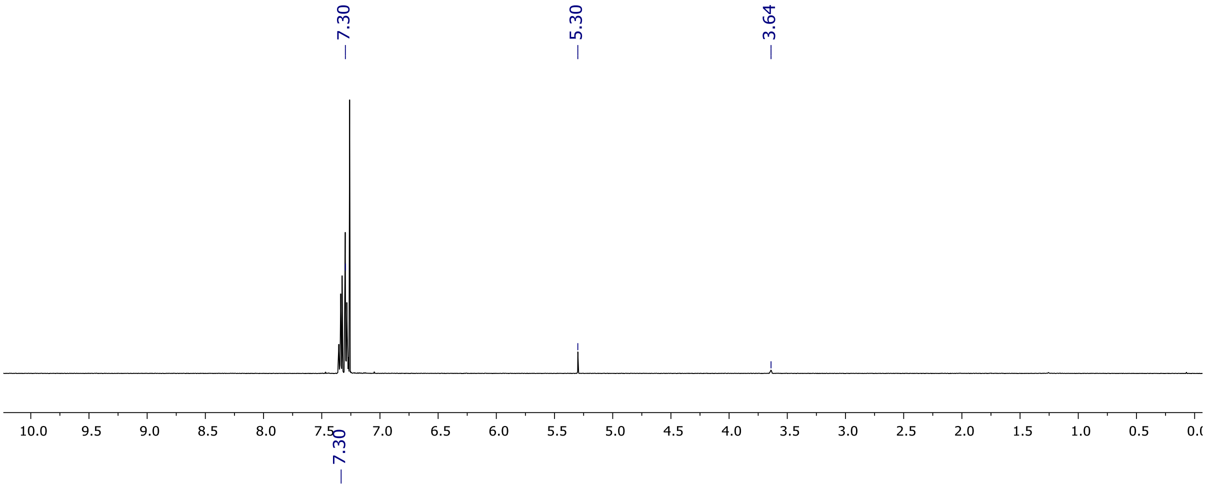

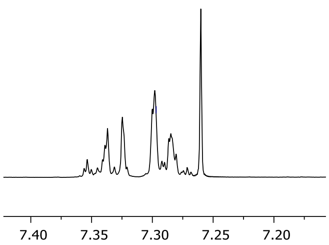

$^{13}C\{^1H\}$

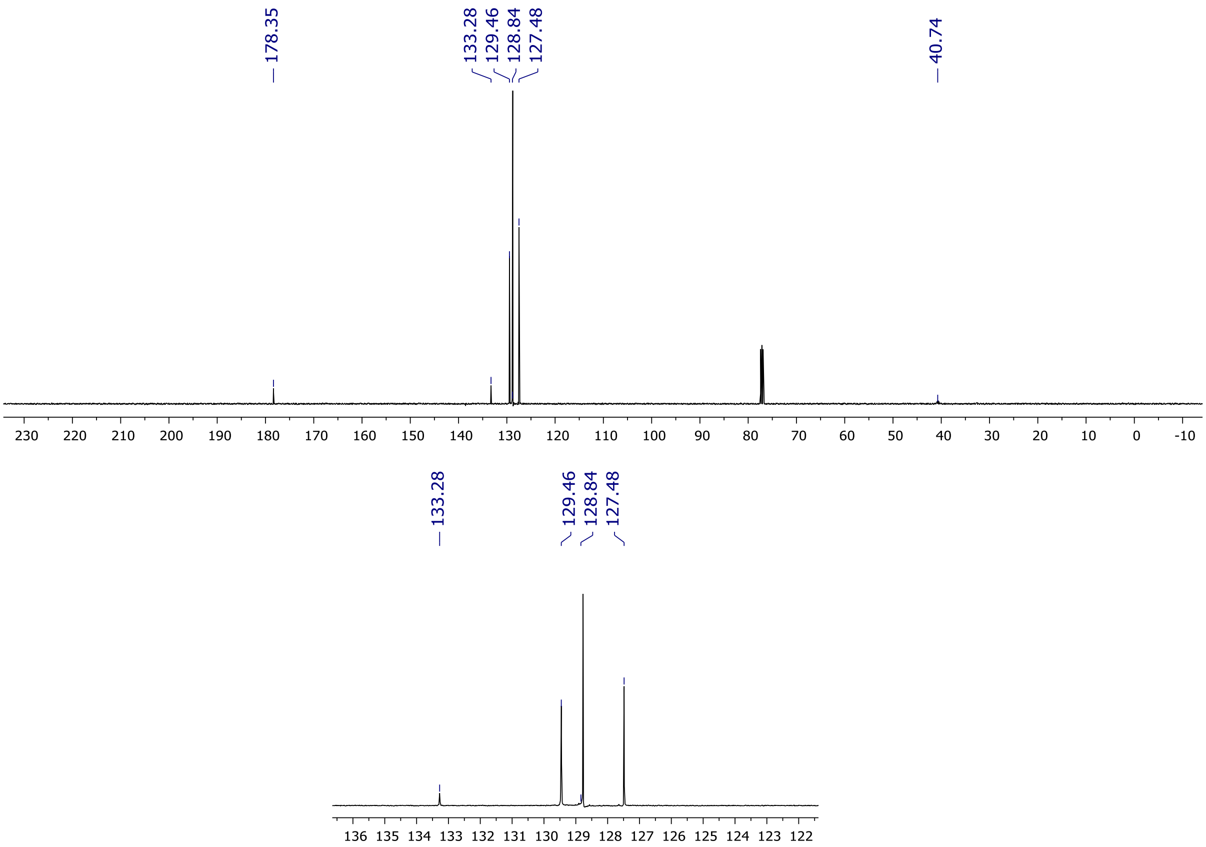
178.35
133.28
129.46
128.84
127.48
40.74
230 220 210 200 190 180 170 160 150 140 130 120 110 100 90 80 70 60 50 40 30 20 10 0 -10
133.28
129.46
128.84
127.48
136 135 134 133 132 131 130 129 128 127 126 125 124 123 122

^2H

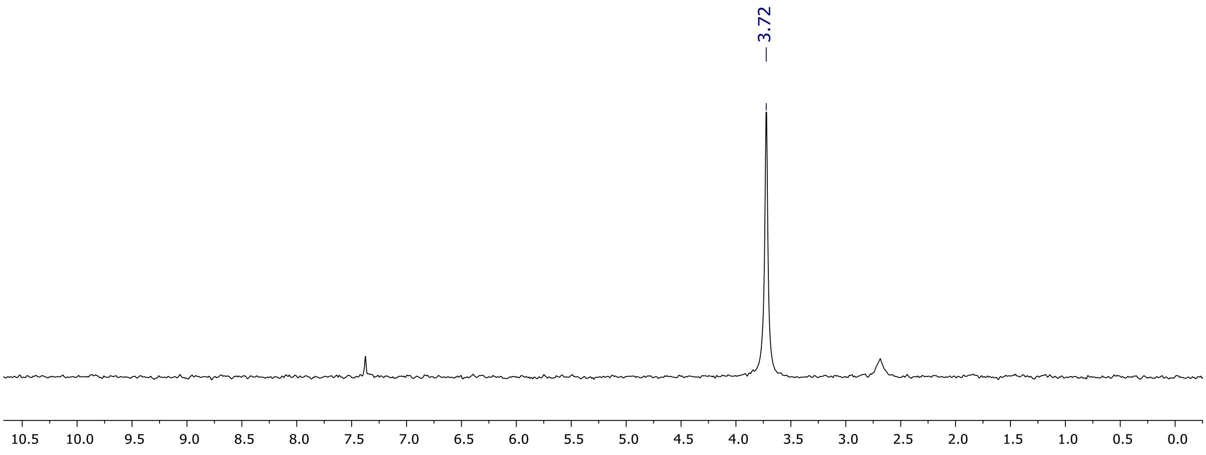
3.72
10.5 10.0 9.5 9.0 8.5 8.0 7.5 7.0 6.5 6.0 5.5 5.0 4.5 4.0 3.5 3.0 2.5 2.0 1.5 1.0 0.5 0.0

2,2-d$_2$-2-phenylethanol ($PhCD_2CH_2OH$)

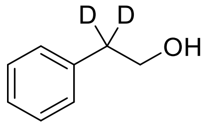

^1H

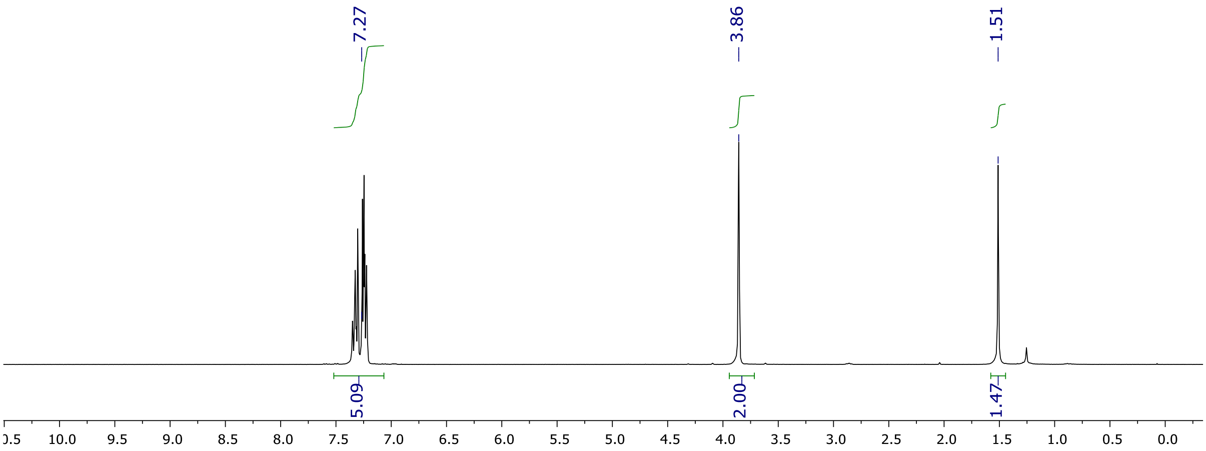

$^{13}C\{^1H\}$

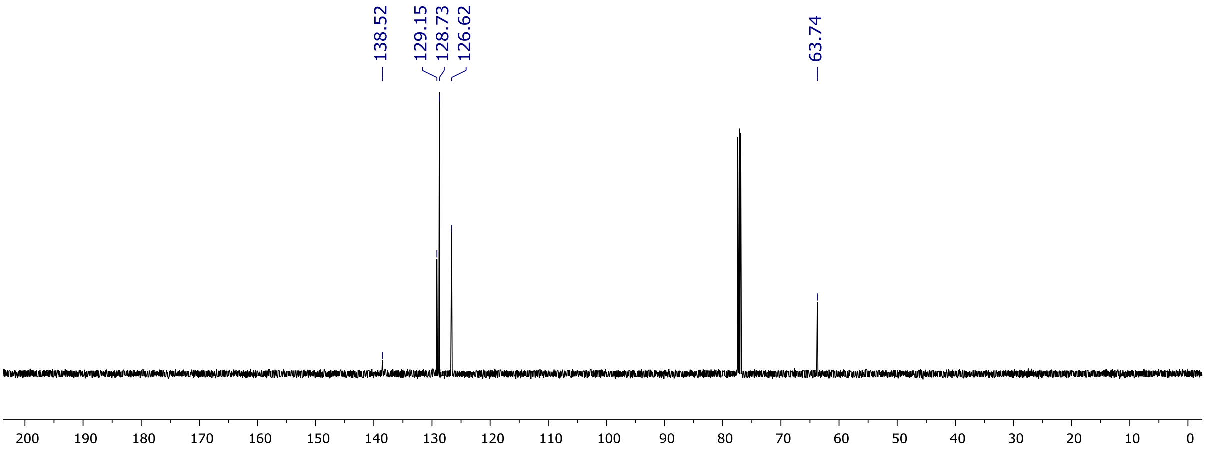

^2H (Deuterated solvent peak observed)

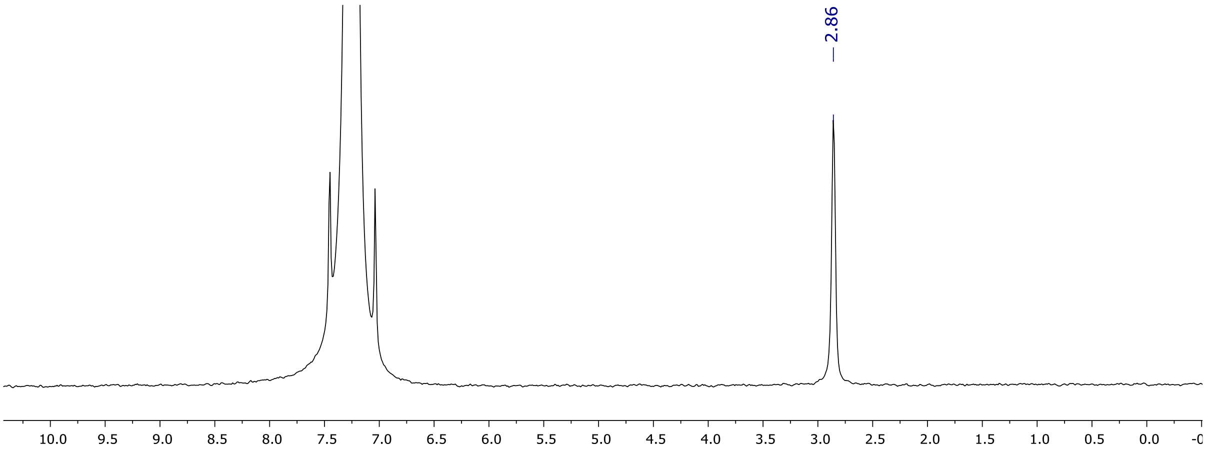

2,2-d$_2$-phenylacetaldehyde ($PhCD_2CHO$, Crude material immediately reacted further)

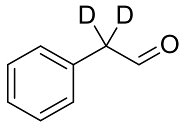

^{1}H

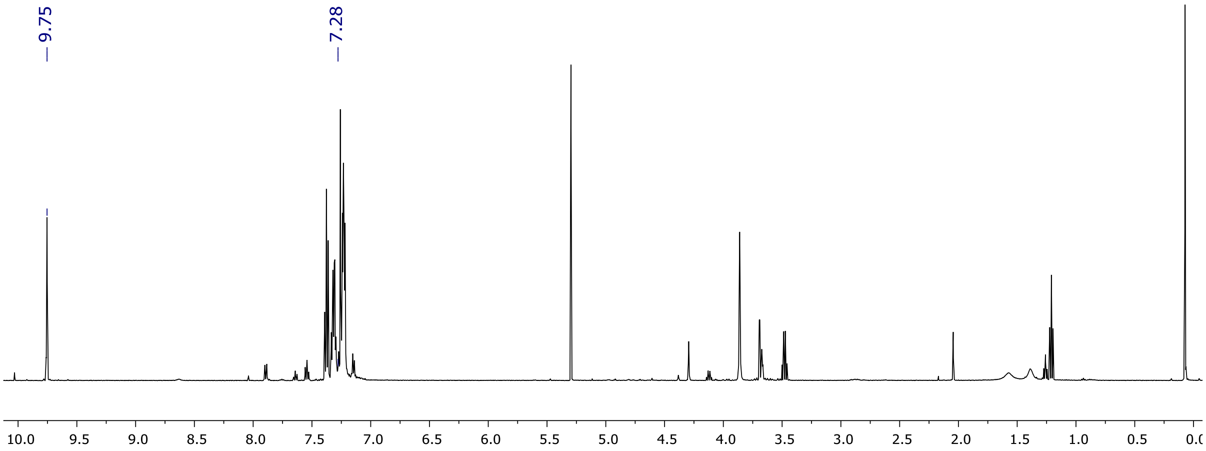

1,1-d$_2$-allylbenzene ($PhCD_2CHCH_2$)

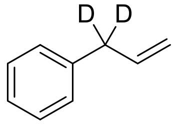

^{1}H

Residual protic signal at 3.39 ppm, some alkyl side-products from Wittig reaction also observed at ^{1}H δ = 2.31 & 1.88 ppm respectively

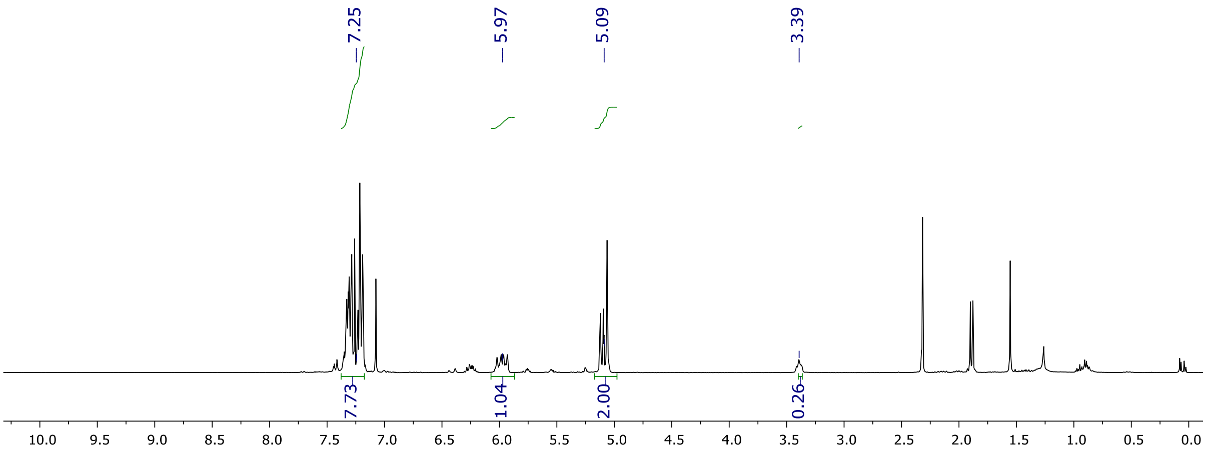

^{2}H

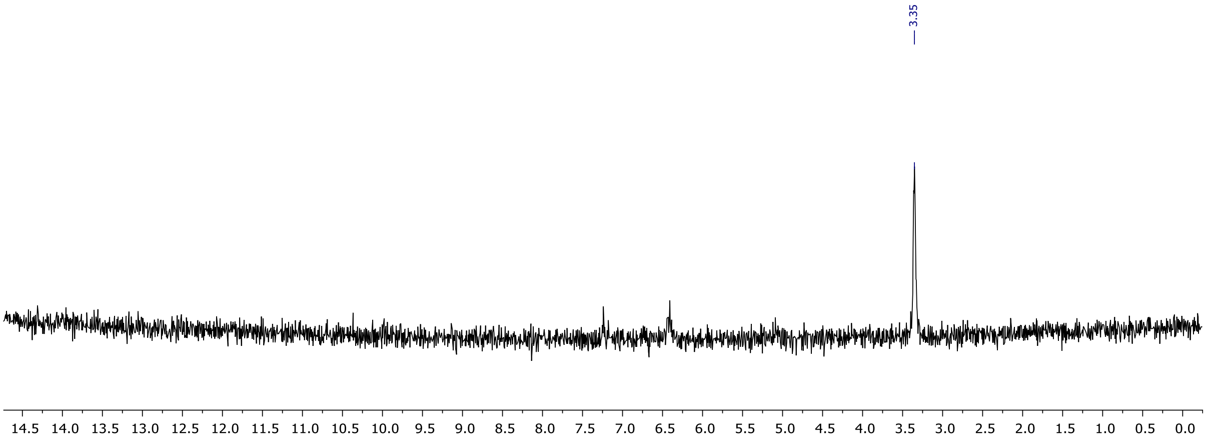

$^{13}C\{^{1}H\}$

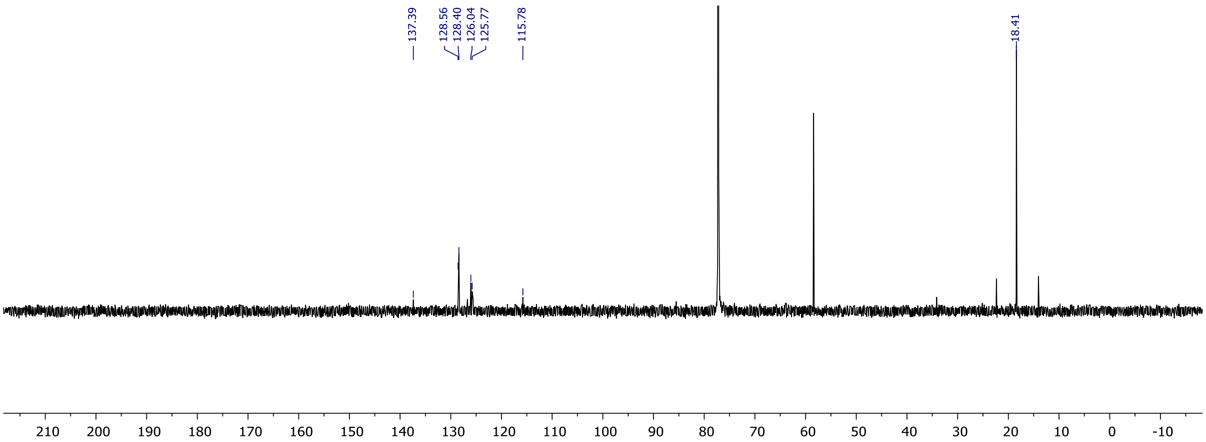

3,3-d_2-allylbenzene

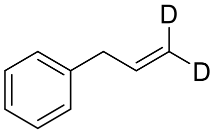

Residual protic signal at 5.07 ppm

^{1}H

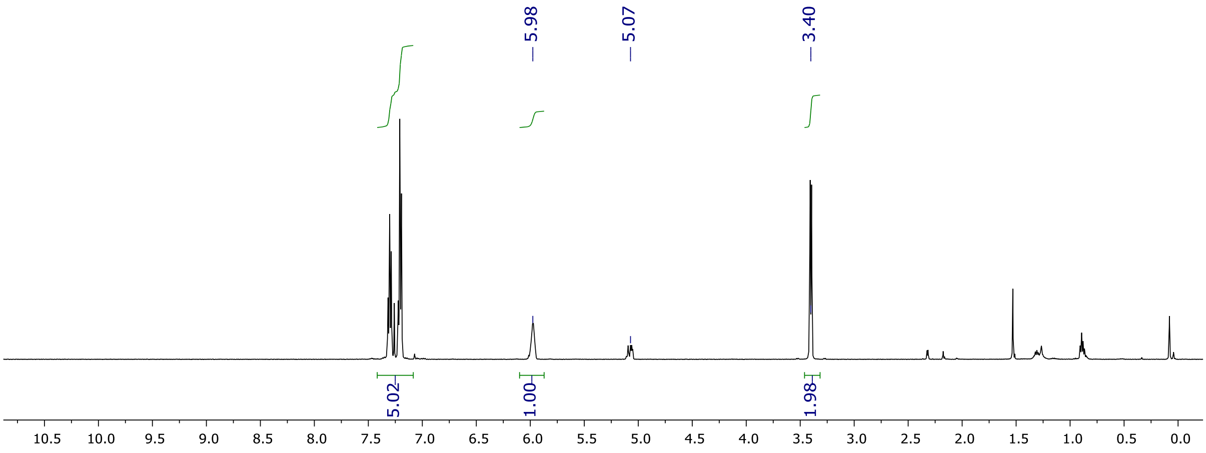

$^{13}C\{^1H\}$

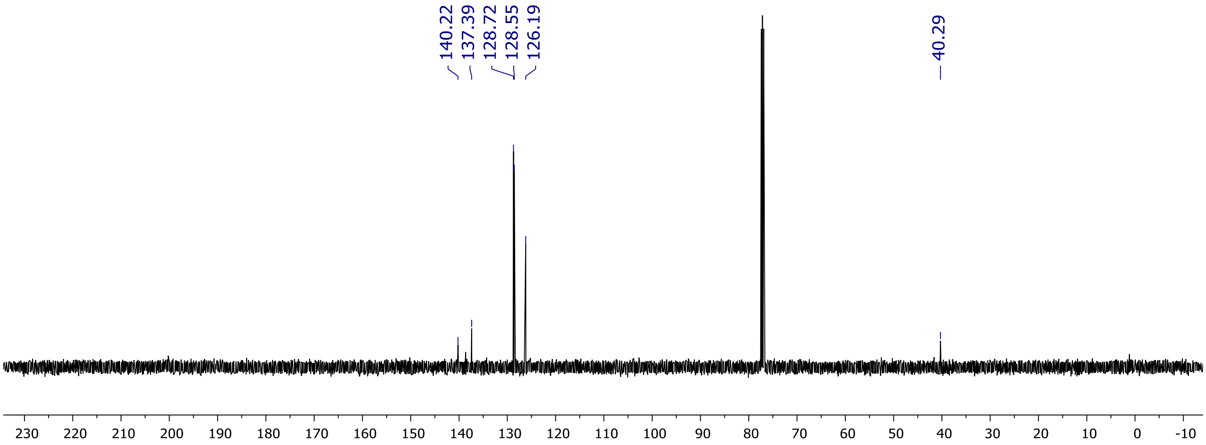

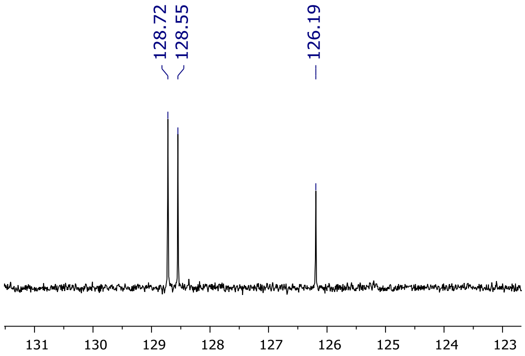

^2H

$CDCl_3$ peak at 7.26 ppm

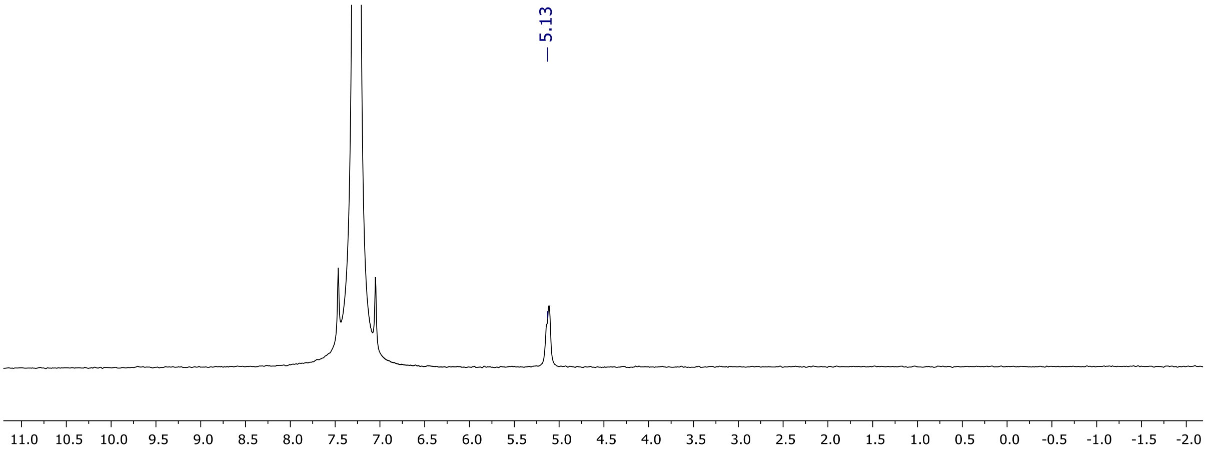

10.3 Iron hydride (4) in catalysis

Hydride conversion monitored by consumption of alkenyl proton peaks. ● indicating starting material (5.95, 5.08, 3.28 ppm), ● indicating cis and ● indicating trans

2 hours, 60°C

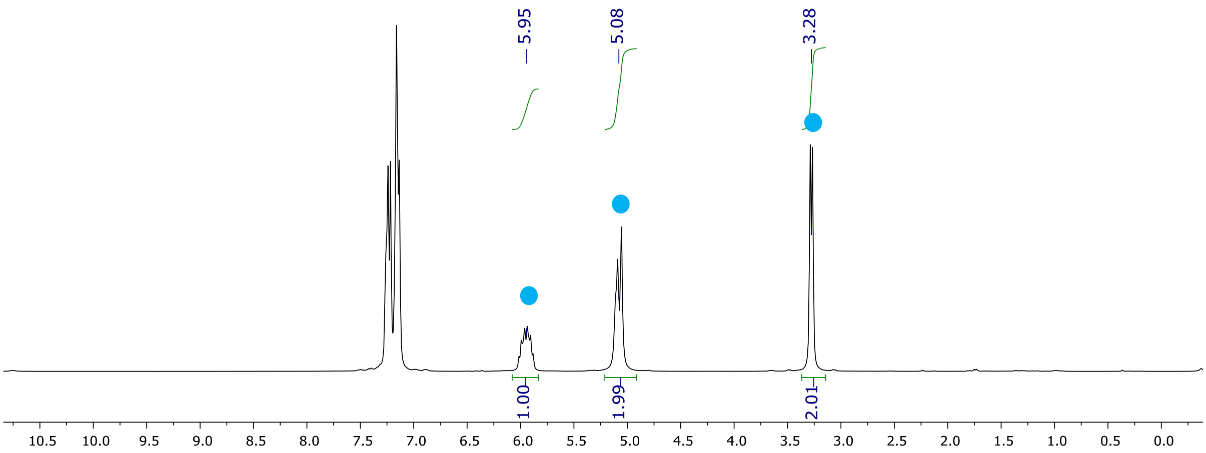

16 hours, 60°C

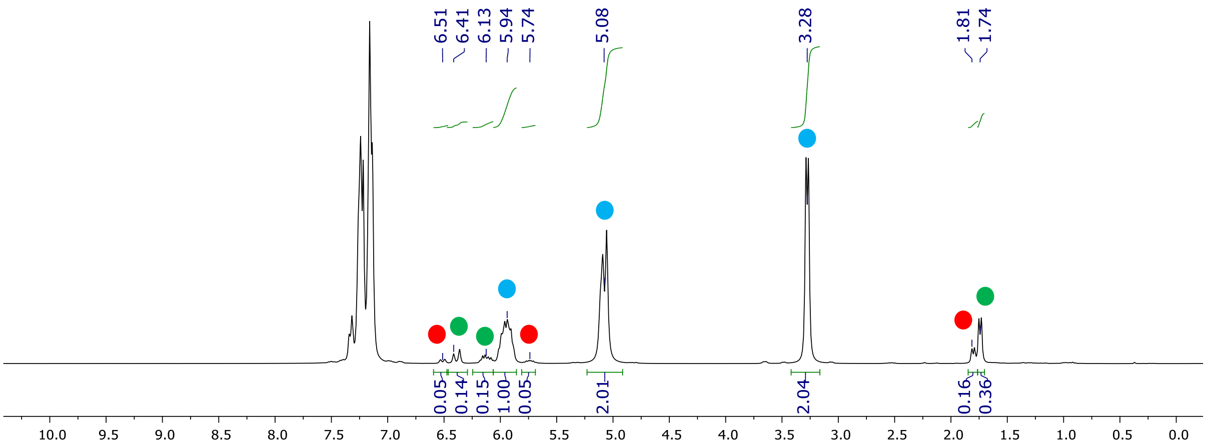

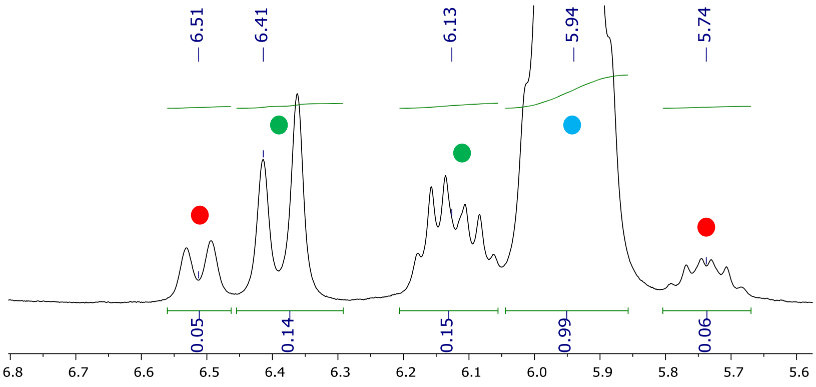

24 hours, 80°C

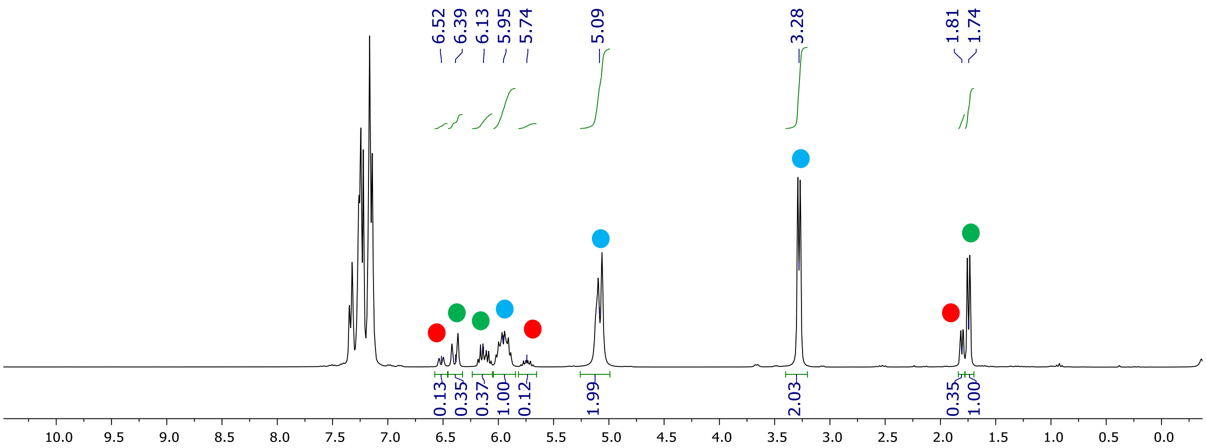

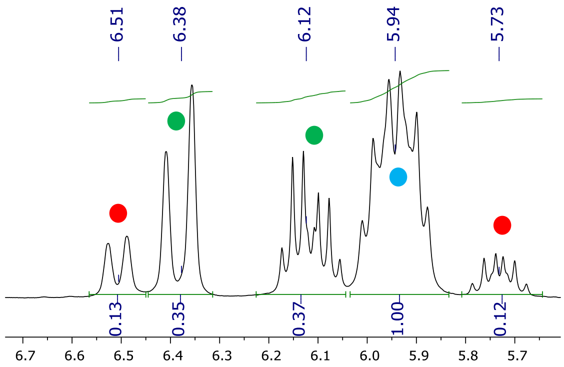

10.4 Radical Clock Test

^{1}H immediately before addition of (Chloromethyl)cyclopropane (Starting material indicated by ●, trans product by ●, cis product by ●)

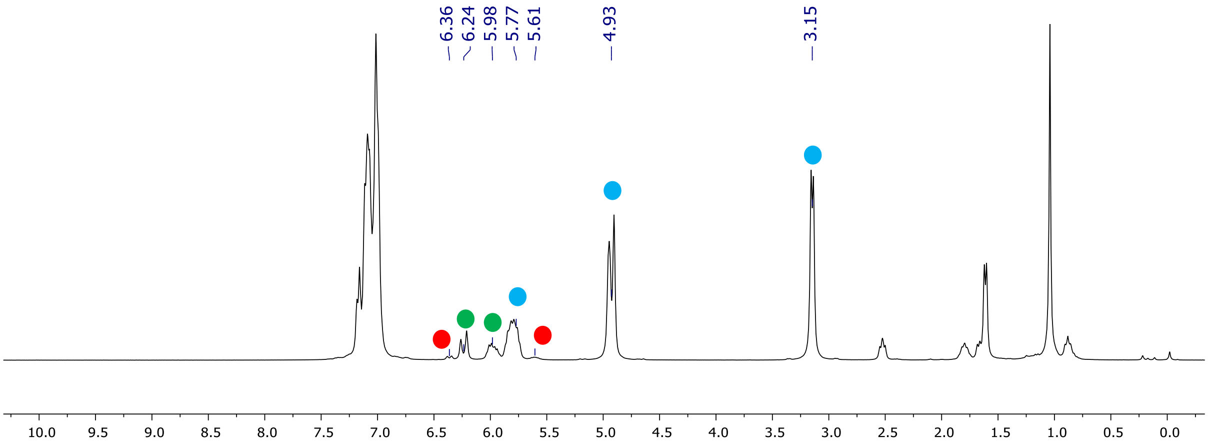

^{1}H 4 hours after addition of (Chlormethyl)cyclopropane (Starting material indicated by ●, trans product by ●, cis product by ●, radical trap indicated by ● (the remaining peak (^{1}H δ = 1.02 ppm, 1H) is obscured by the methyl groups on the pinacol backbone))

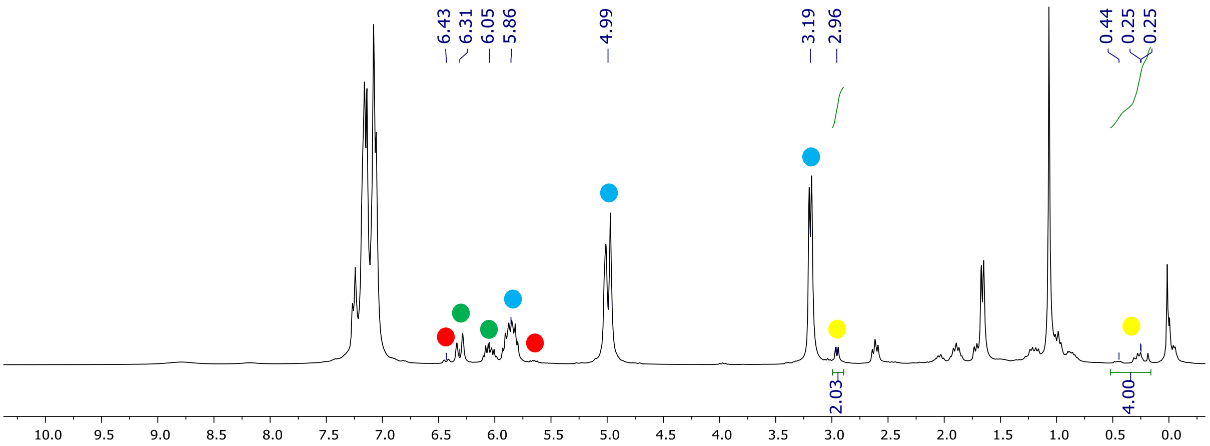

10.5 Product NMRs

3B

(*E*)-1-(2-methylbenzyl)prop-1-ene

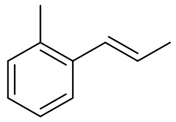

Trace amounts of hydrogenated side-product observed – alkyl peaks marked with ● (^{1}H 2.32, 2.05, 1.11 ppm and ^{13}C{^{1}H} 35.6, 20.1, 14.3 ppm respectively)

^{1}H

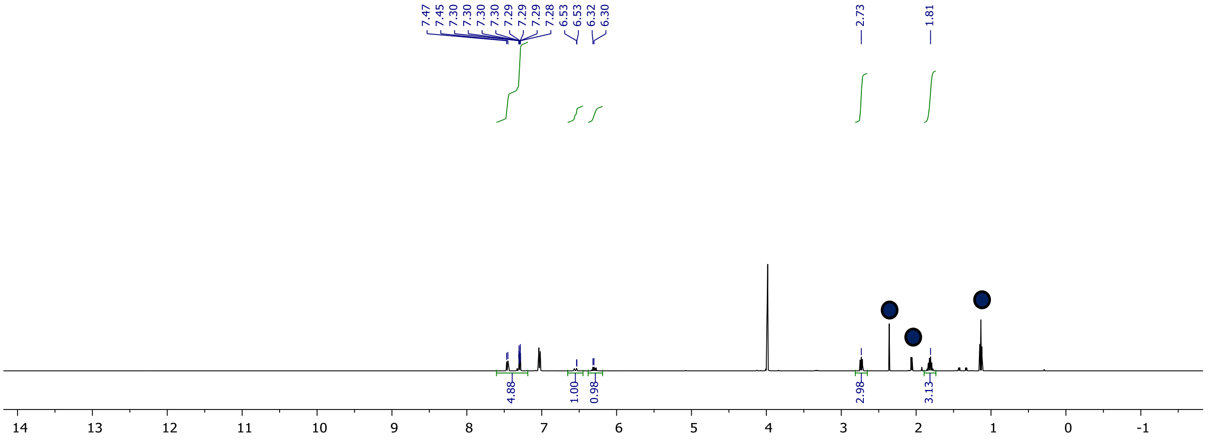

$^{13}C\{^1H\}$

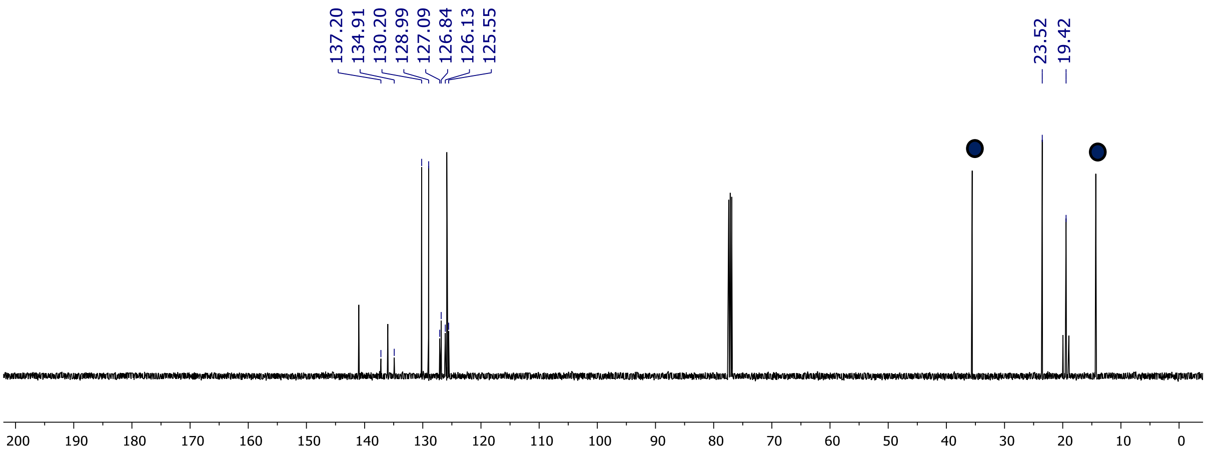

3C

(*E*)-1-(3-methylbenzyl)prop-1-ene

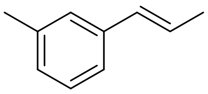

Trace amounts of hydrogenated side-product observed – alkyl peaks marked with ● (^1H 2.55, 1.65, 0.96 ppm and $^{13}C\{^1H\}$ 38.0, 24.6, 13.9 ppm respectively)

^1H

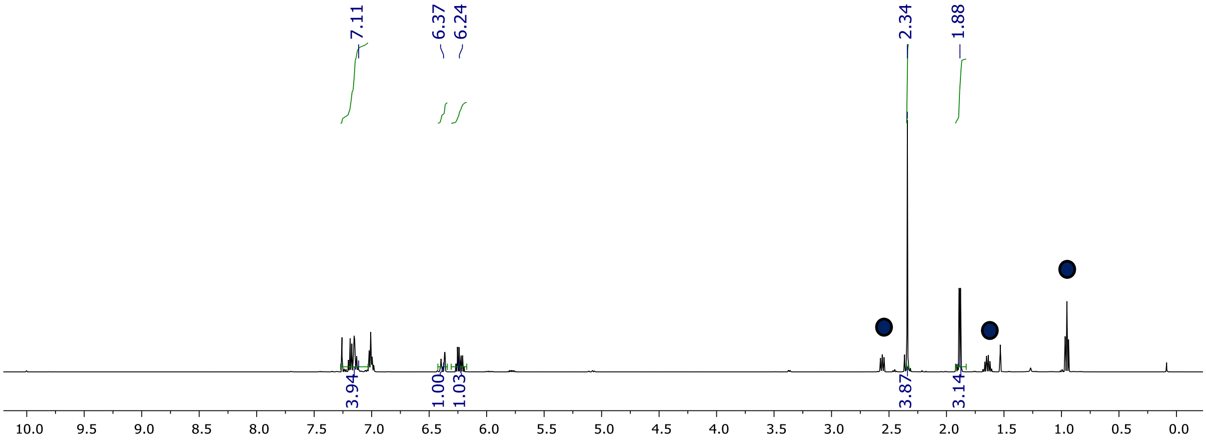

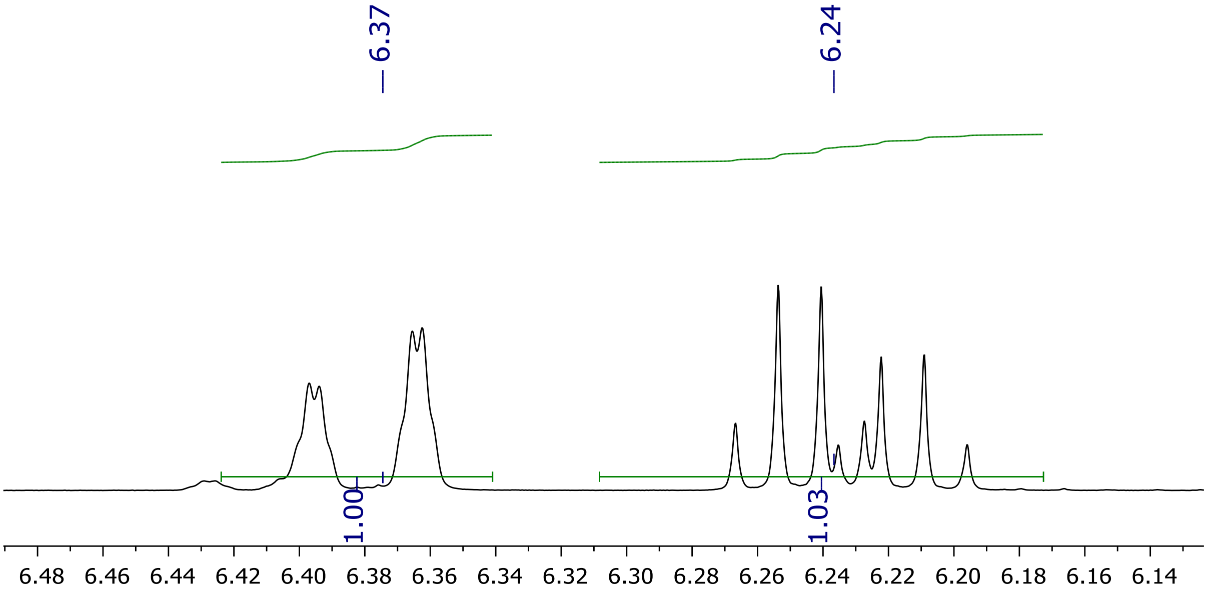

$^{13}C\{^{1}H\}$

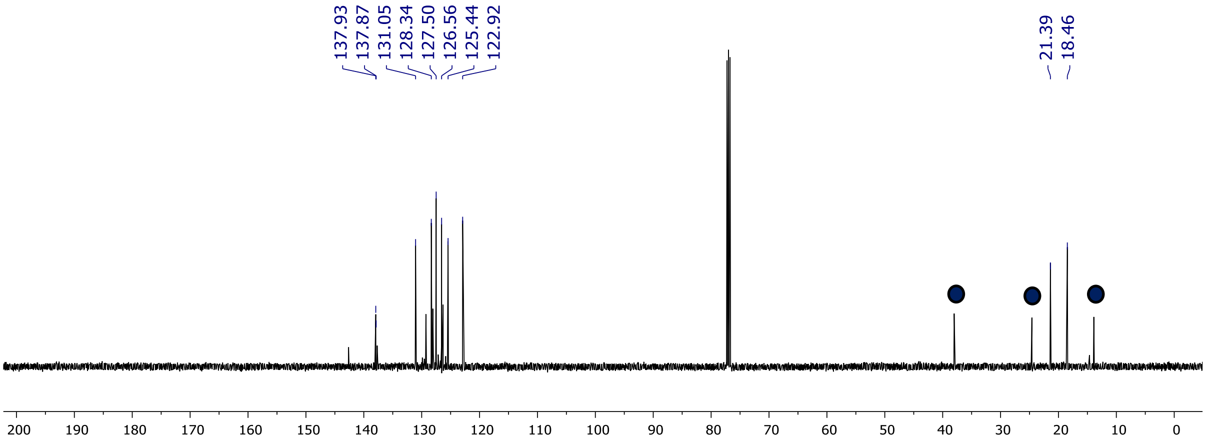

3D

(E)-1-(4-methylbenzyl)prop-1-ene

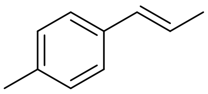

Trace amounts of hydrogenated side-product observed – alkyl peaks marked with ● (^{1}H 2.56, 1.65, 0.96 ppm and $^{13}C\{^{1}H\}$ 37.9, 24.5, 13.9 ppm respectively)

^{1}H

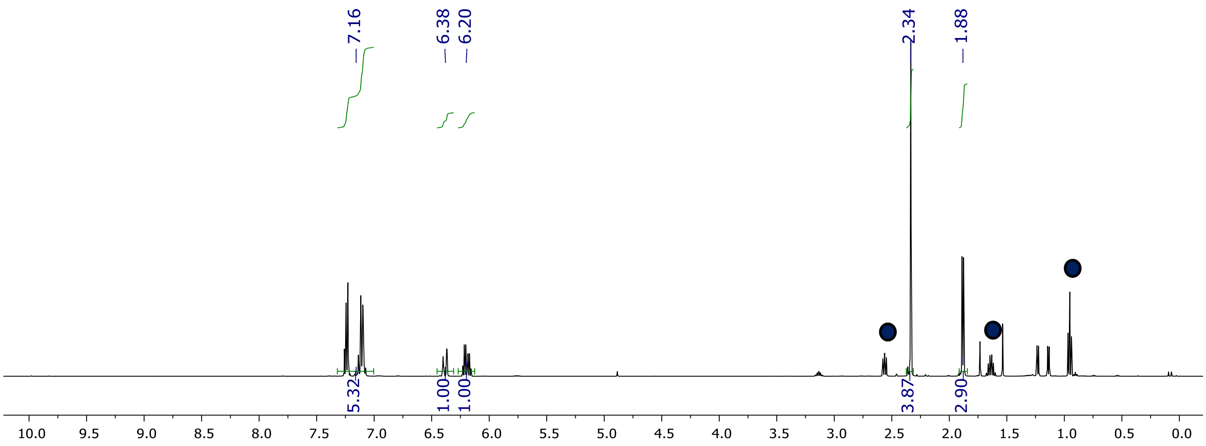

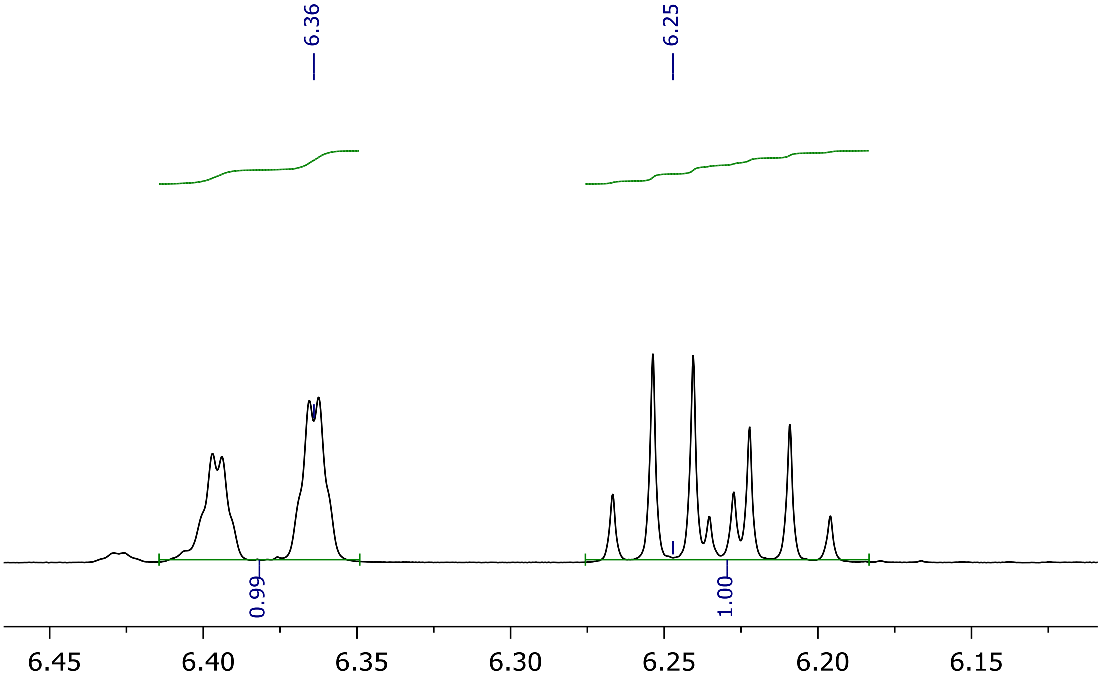

$^{13}C\{^1H\}$

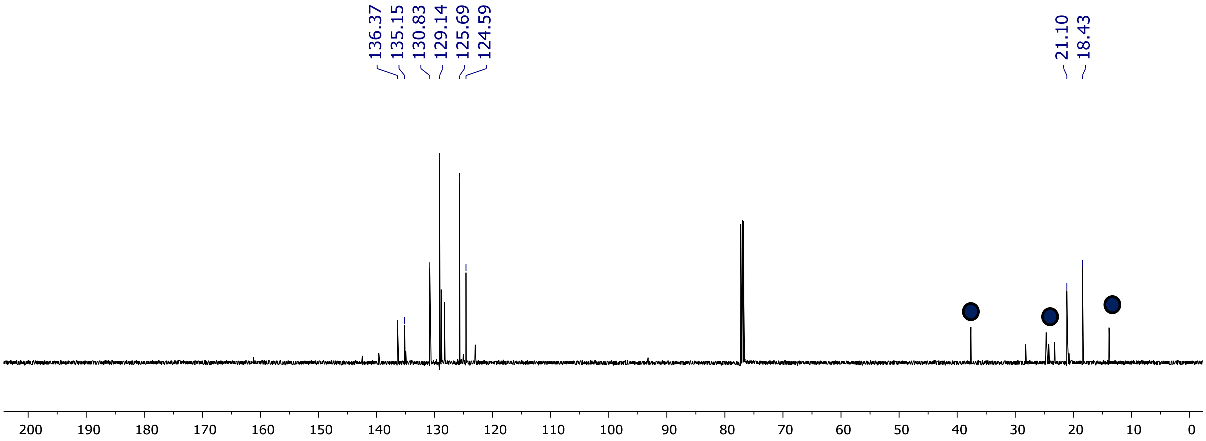

3E

(*E*)-1-(2-methoxybenzyl)prop-1-ene

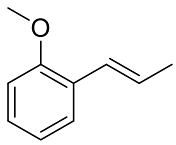

Trace amounts of hydrogenated side-product observed – alkyl peaks marked with ● ([1]H 2.60, 1.63, 0.97 ppm and $^{13}C\{^{1}H\}$ 32.2, 22.9, 14.1 ppm respectively)

[1]H

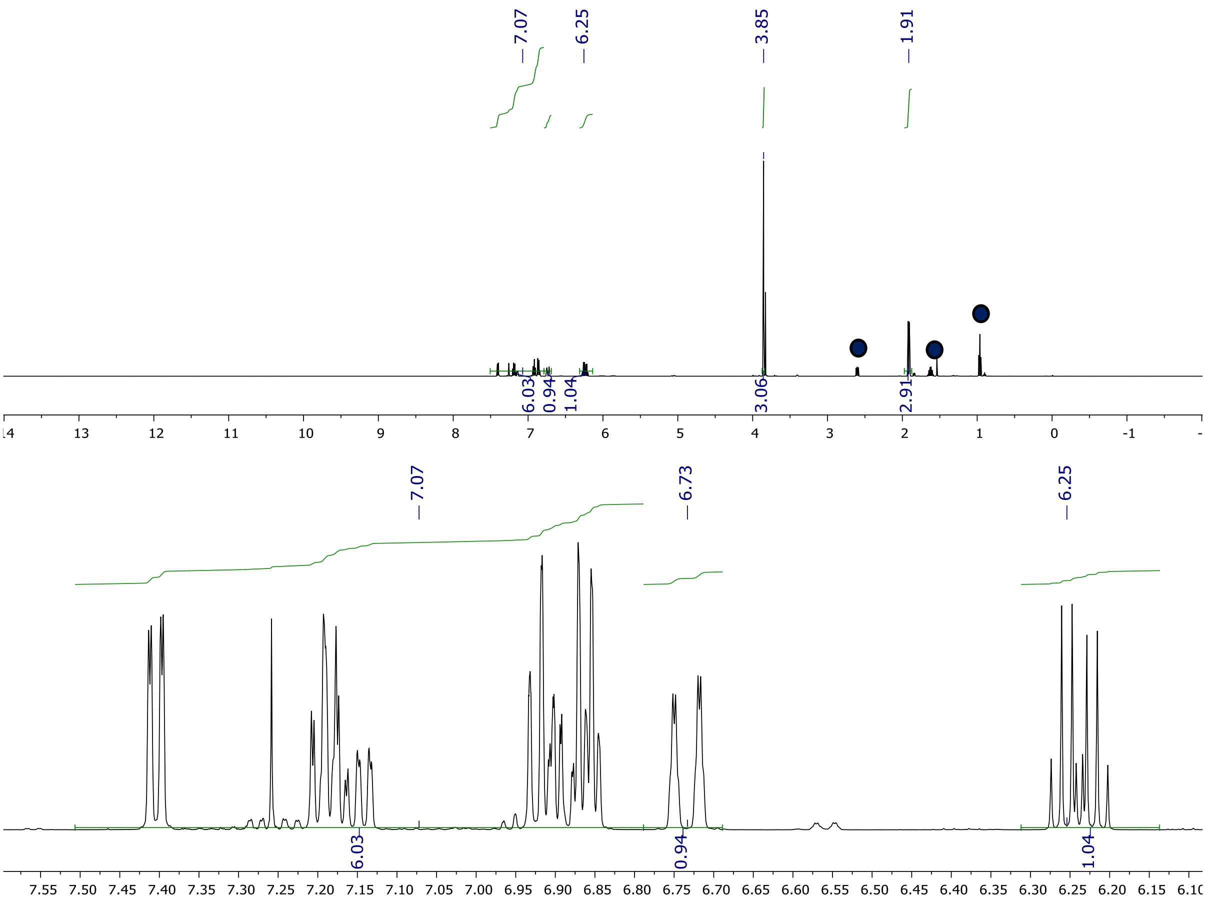

$^{13}C\{^{1}H\}$

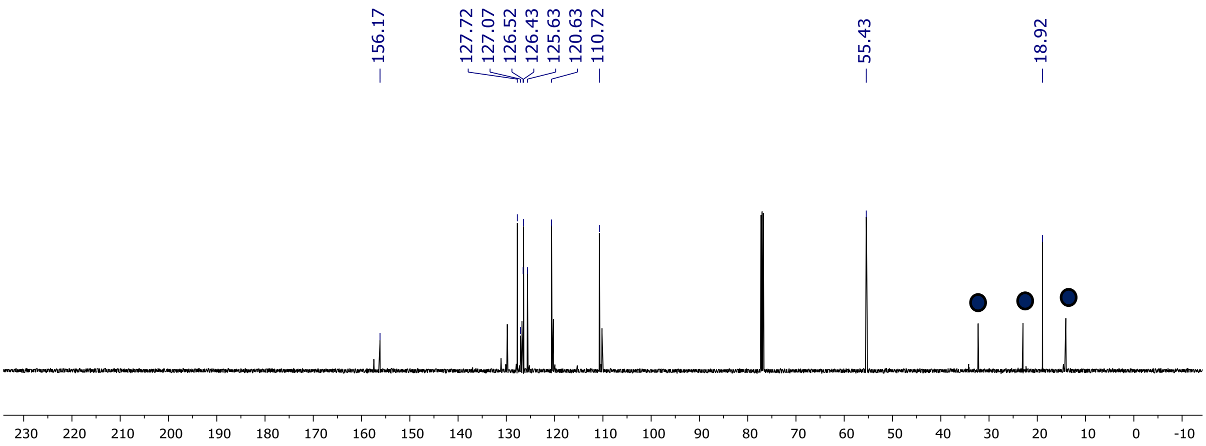

3F

(*E*)-1-(4-methoxybenzyl)prop-1-ene

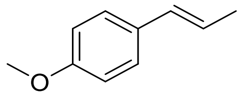

Trace amounts of hydrogenated side-product observed – alkyl peaks marked with ● (^{1}H 2.63, 1.64, 0.94 ppm and $^{13}C\{^{1}H\}$ 37.1, 24.8, 13.8 ppm respectively)

^{1}H

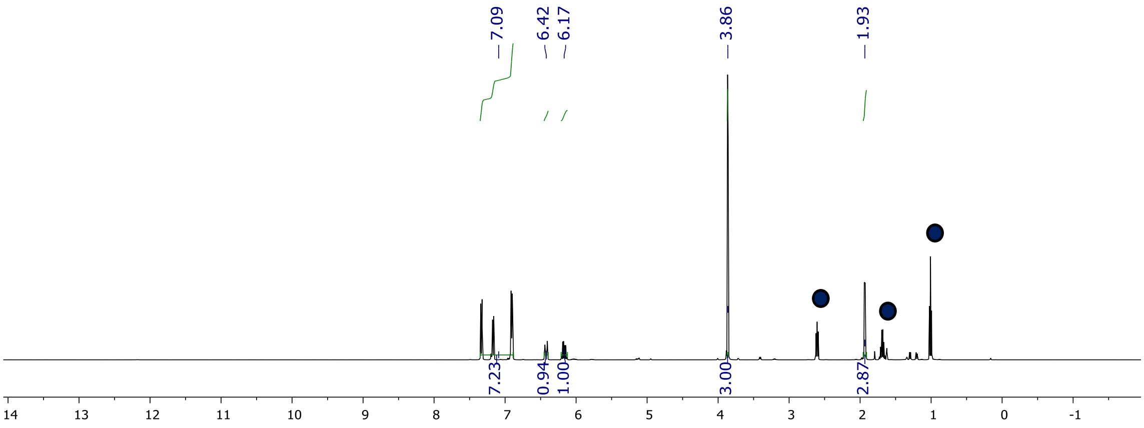

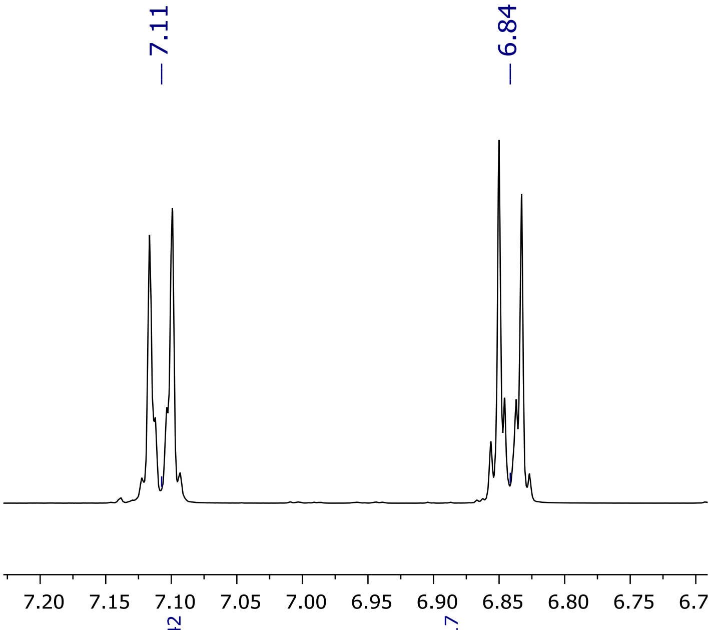
7.11
6.84
7.20 7.15 7.10 7.05 7.00 6.95 6.90 6.85 6.80 6.75 6.7

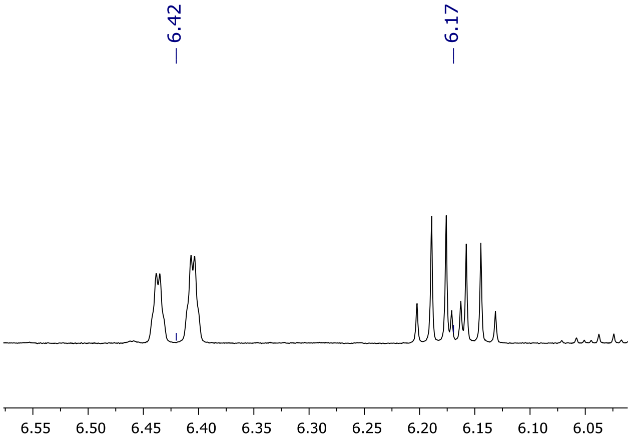
6.42
6.17
6.55 6.50 6.45 6.40 6.35 6.30 6.25 6.20 6.15 6.10 6.05

$^{13}C\{^1H\}$

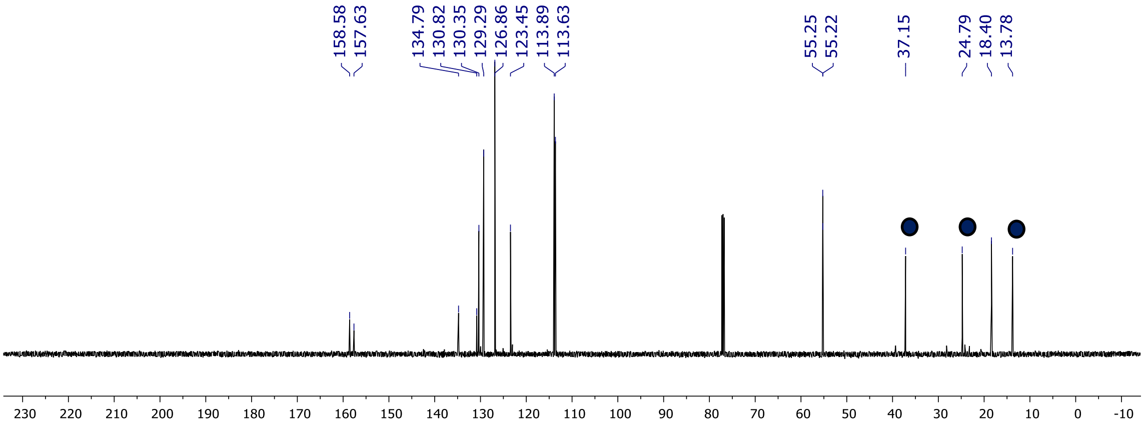
158.58
157.63
134.79
130.82
130.35
129.29
126.86
123.45
113.89
113.63
55.25
55.22
37.15
24.79
18.40
13.78
230 220 210 200 190 180 170 160 150 140 130 120 110 100 90 80 70 60 50 40 30 20 10 0 -10

3G

(*E*)-1-(4-Fluorophenyl)prop-1-ene

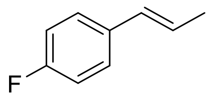

Trace amounts of hydrogenated side-product observed – alkyl peaks marked with ● (^{1}H 2.56, 1.63, 0.94 ppm, ^{13}C{^{1}H} 37.2, 24.6, 13.7 ppm and ^{19}F{^{1}H} 118.2 ppm respectively)

The minor Z product is indicated in the alkenyl region with ● (^{1}H 5.95, 5.78 ppm and ^{19}F{^{1}H} 115.95 respectively), and the starting material is indicated with ● (^{1}H 5.07, 3.37 ppm and ^{19}F{^{1}H} 117.5 ppm respectively)

^{1}H

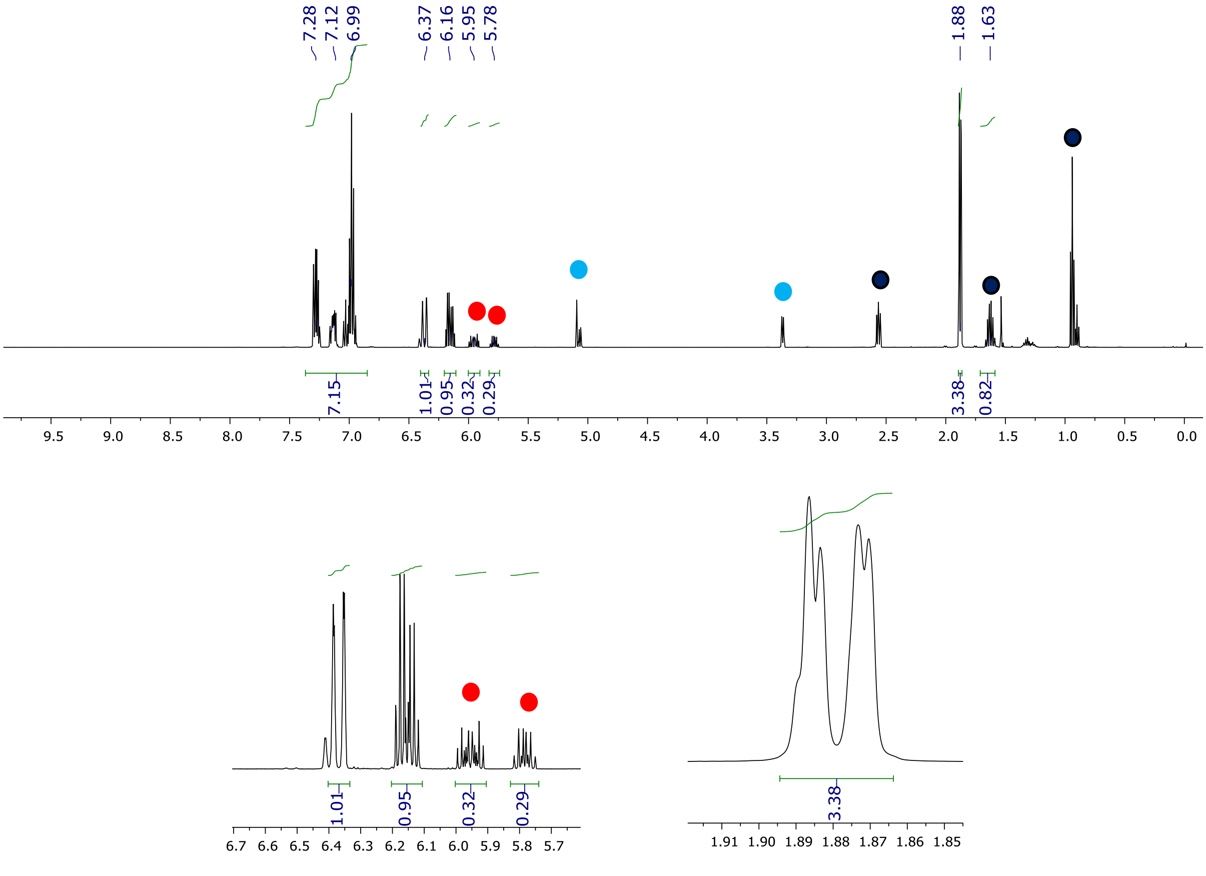

$^{13}C\{^{1}H\}$

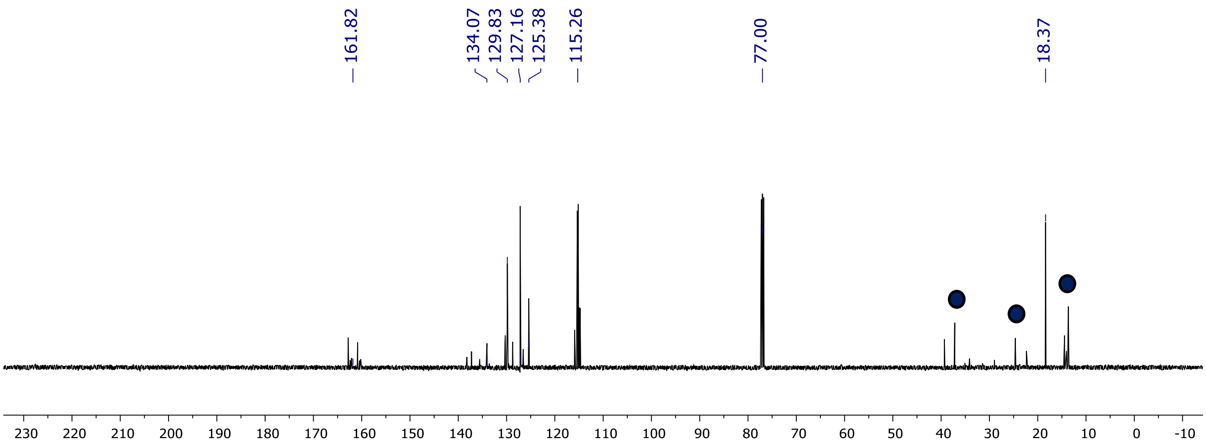

$^{19}F\{^{1}H\}$

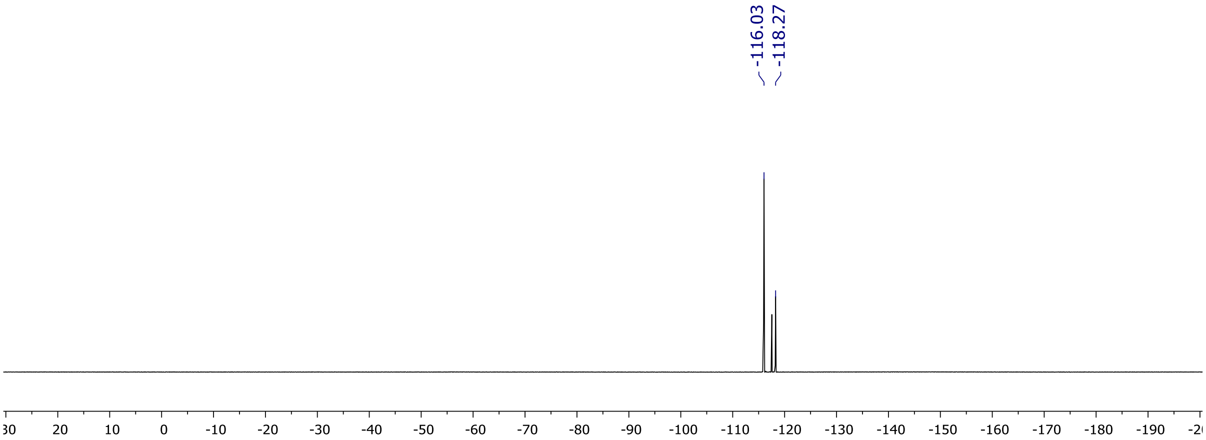

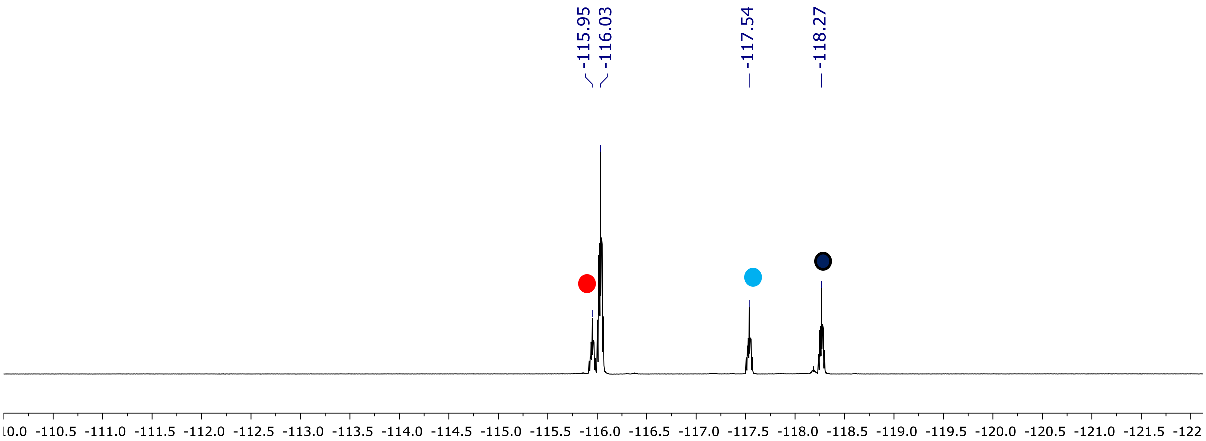

3H

(*E*)-1-(4-Trifluorophenyl)prop-1-ene

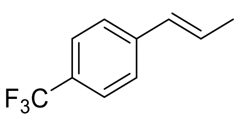

Trace amounts of hydrogenated side-product observed – alkyl peaks marked with ● (^{1}H 2.65, 1.68, 0.96 ppm, $^{13}C\{^{1}H\}$ 39.9, 24.2, 14.0 ppm and $^{19}F\{^{1}H\}$ -62.40 ppm respectively)

The minor Z product is indicated in the alkenyl region with ● (^{1}H 5.96, 5.92 ppm and $^{19}F\{^{1}H\}$ -62.40 respectively), and the starting material is indicated with ● (^{1}H 5.12, 3.45 ppm and $^{19}F\{^{1}H\}$ -62.48 respectively)

^{1}H

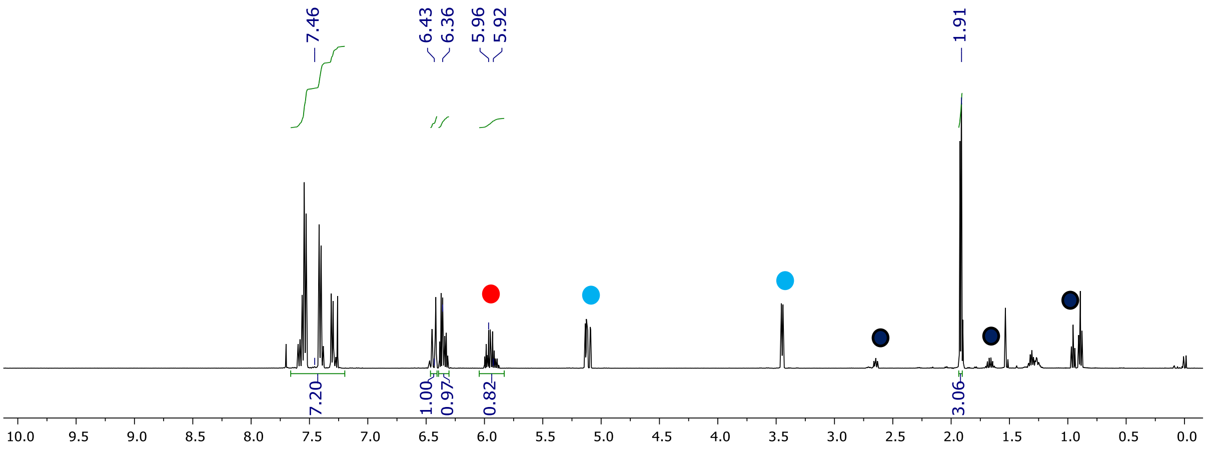

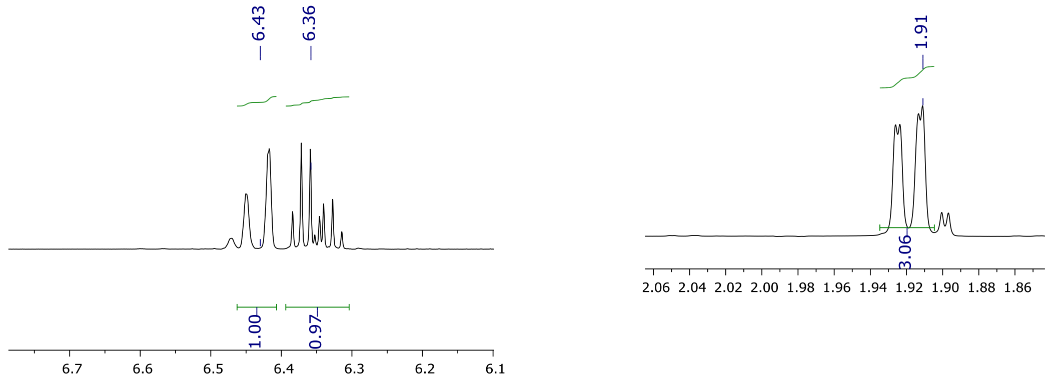

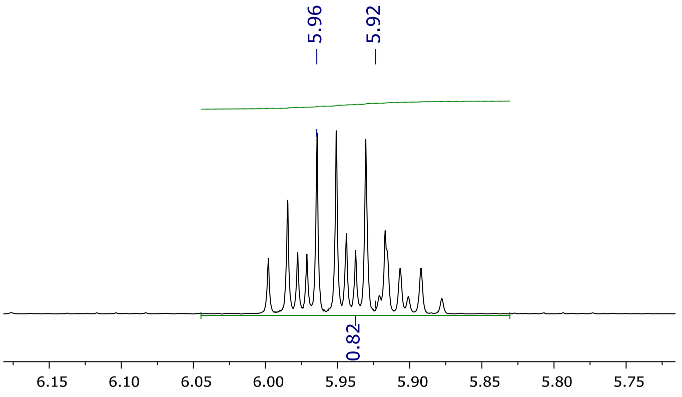

$^{13}C\{^{1}H\}$

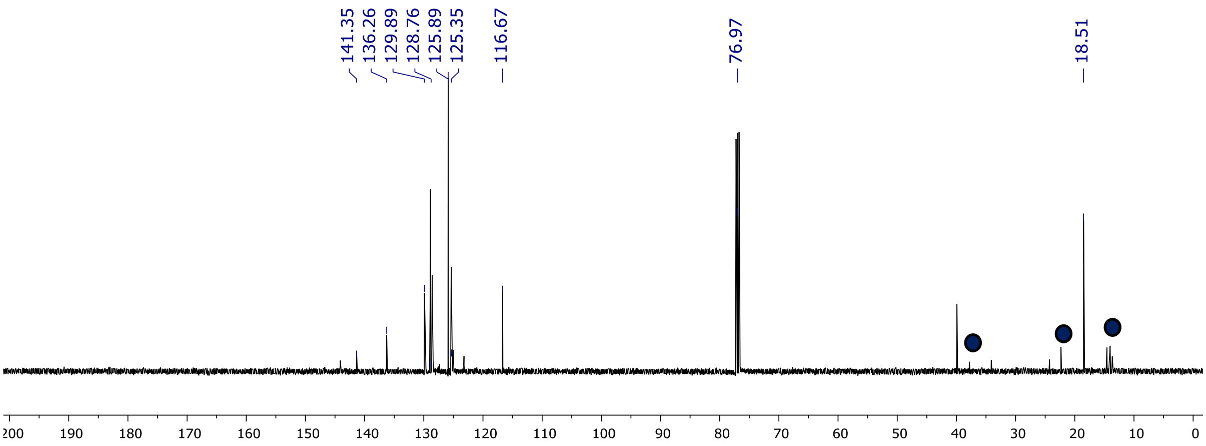

$^{19}F\{^{1}H\}$

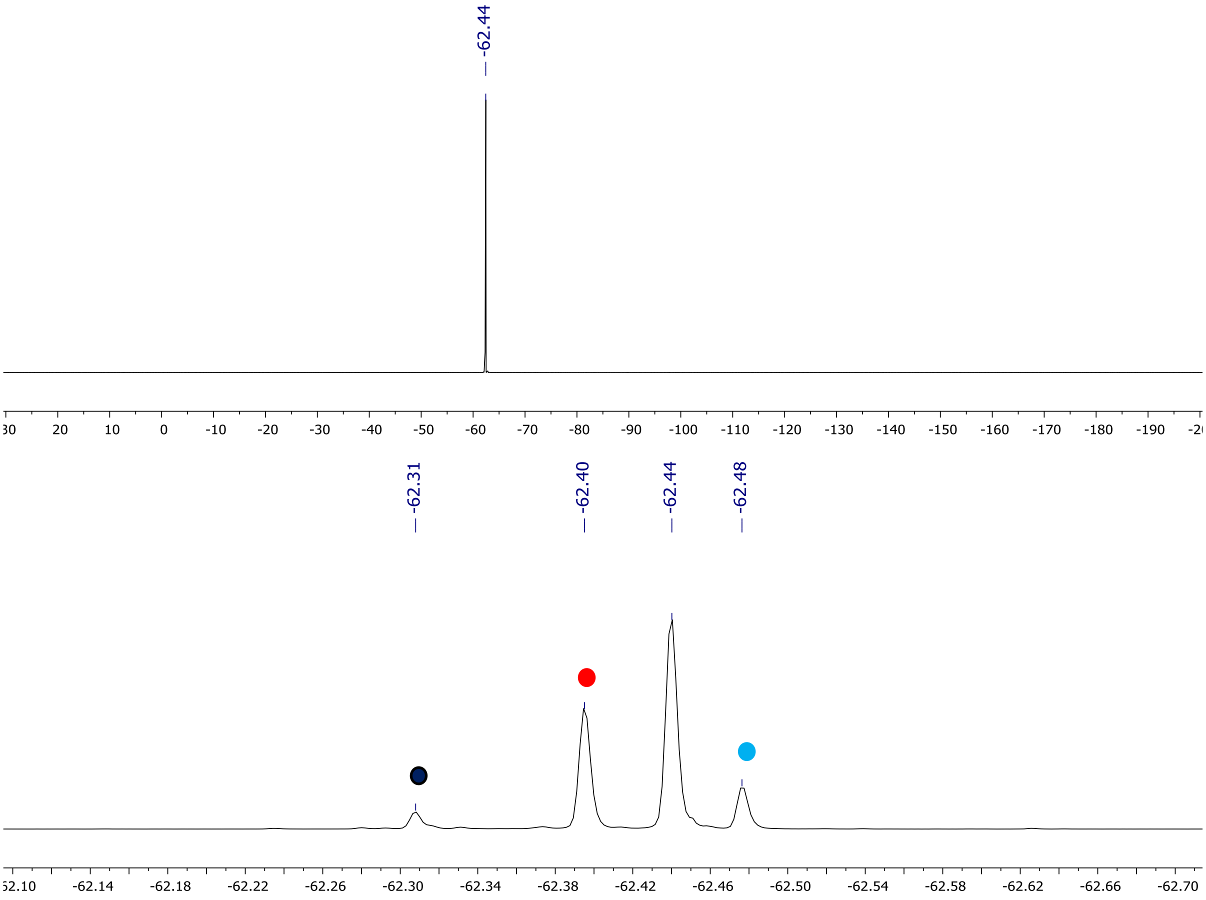

3J

Mixture of Valencene and Isomerised Product – analysed peaks identified

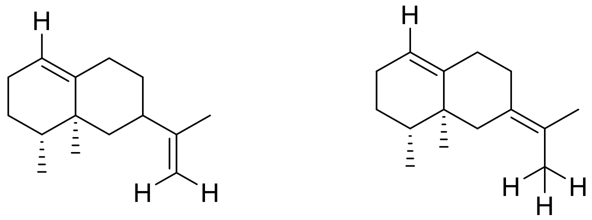

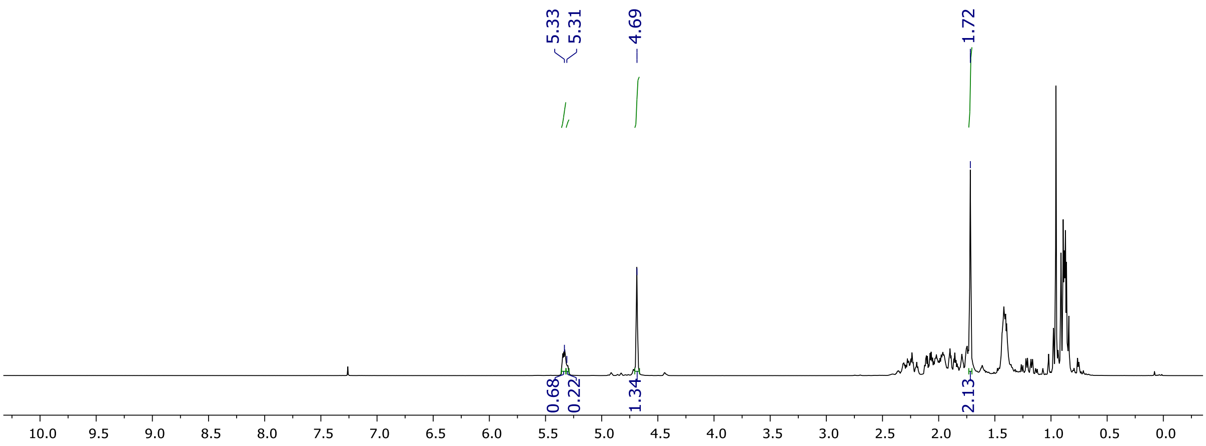

3K

Isosafrole

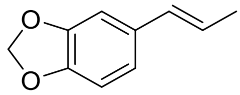

Trace amounts of hydrogenated side-product observed – alkyl peaks marked with ● (^{1}H 2.52, 1.61, 0.94 ppm and ^{13}C{^{1}H} 38.1, 25.0, 14.1 ppm respectively

^{1}H

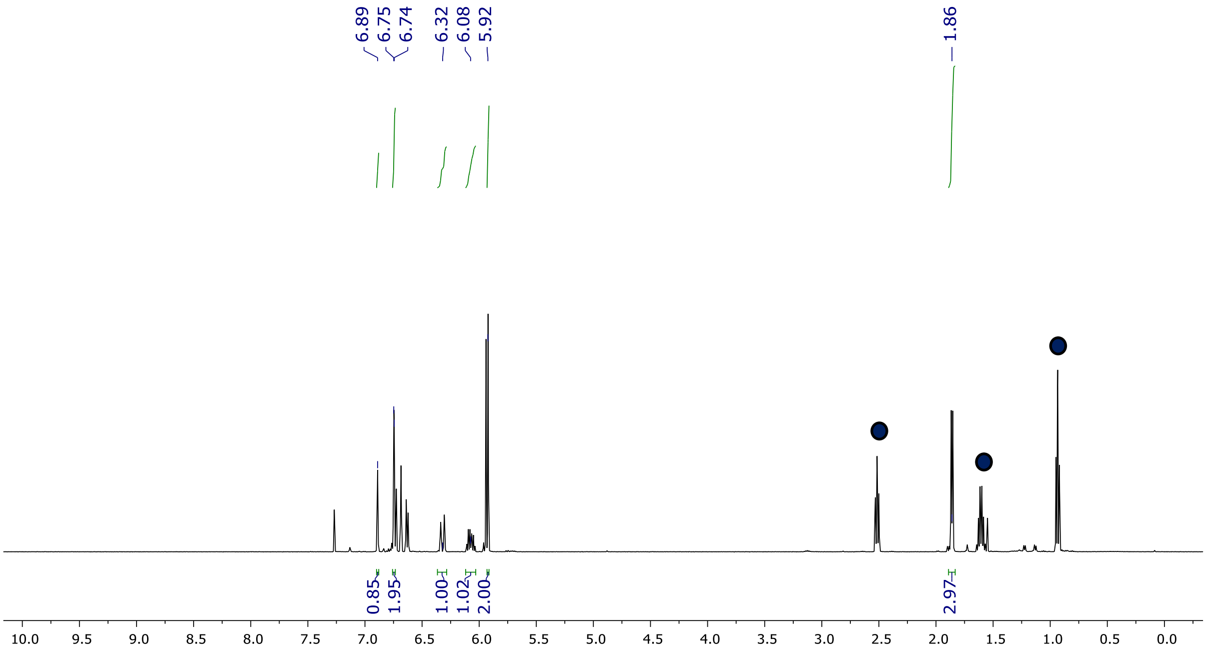

$^{13}C\{^1H\}$

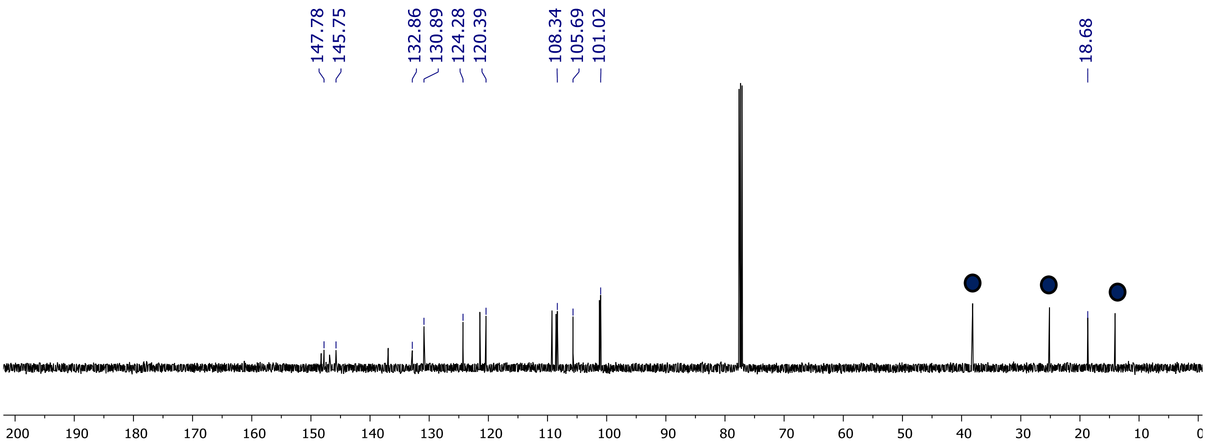

3K*

Piperonal

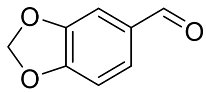

^1H

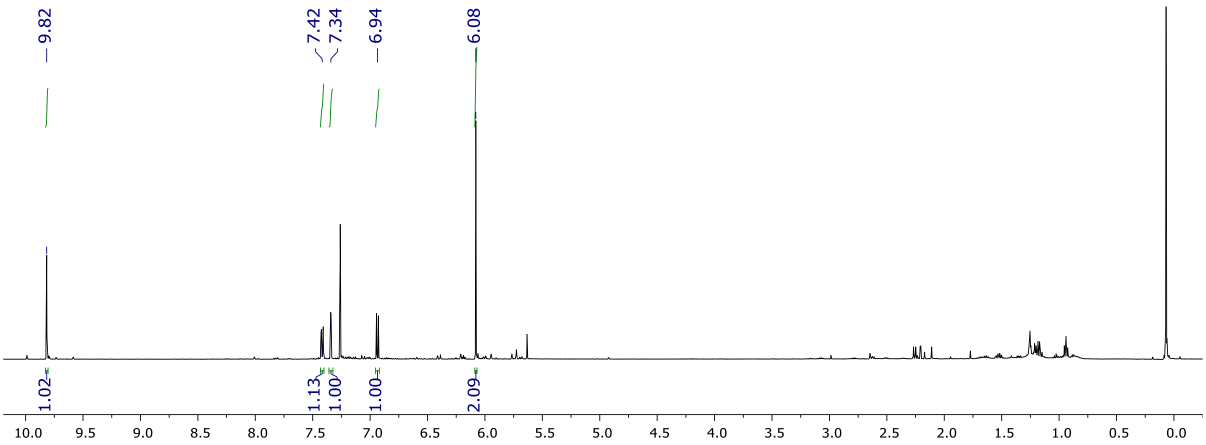

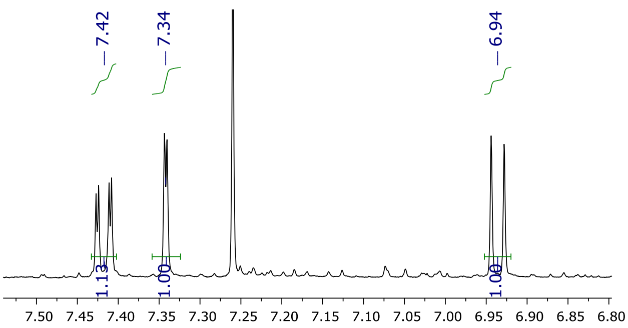

$^{13}C\{^{1}H\}$

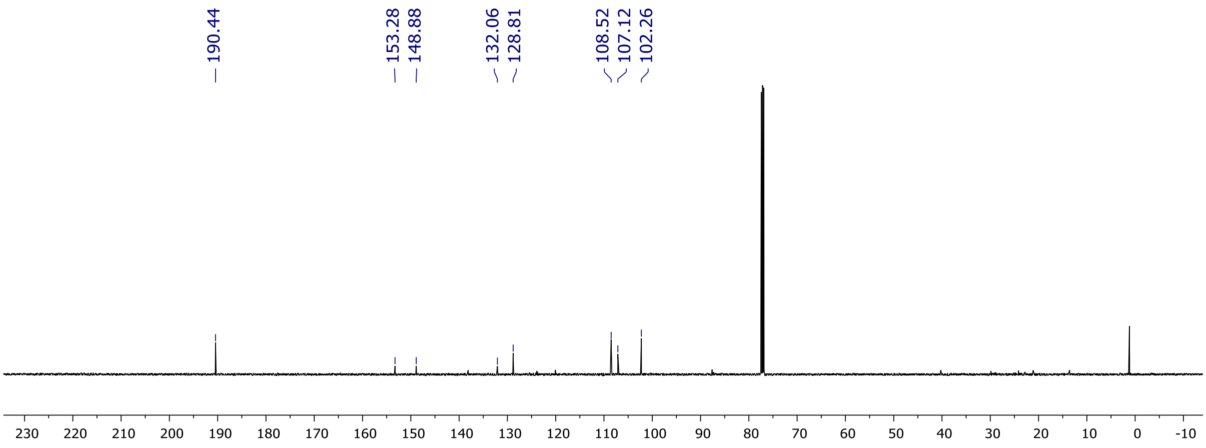

10.5 Linear Alkenes

All reactions monitored through $^{13}C\{^{1}H\}$ NMR

1-Hexene

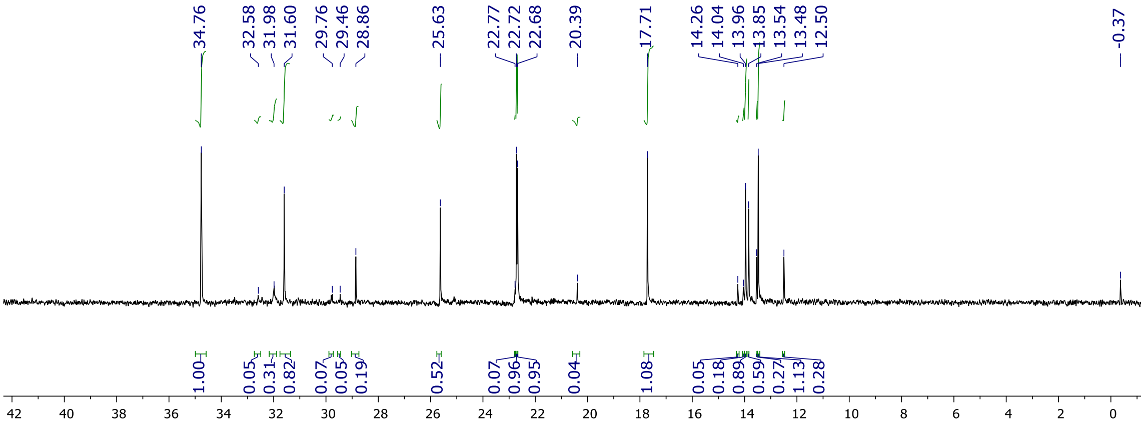

Trans-2-Hexene

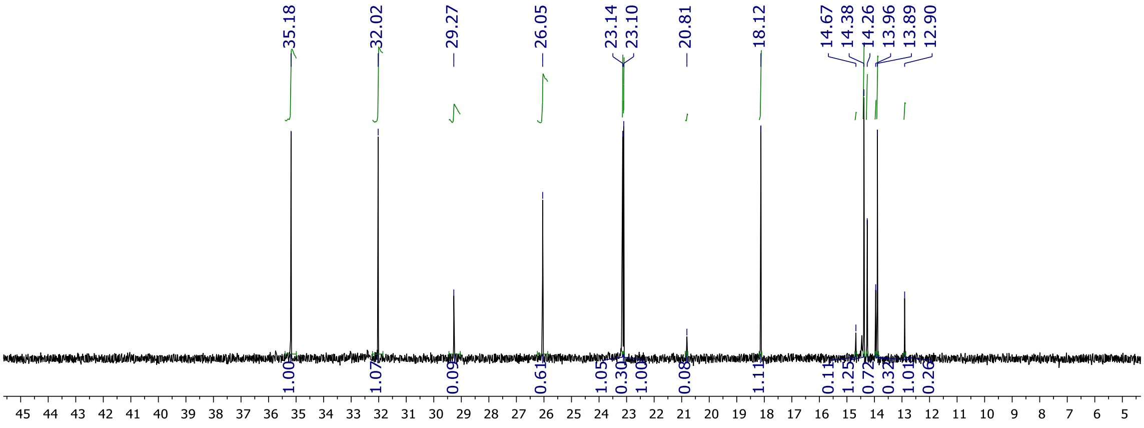

Cis-2-Hexene

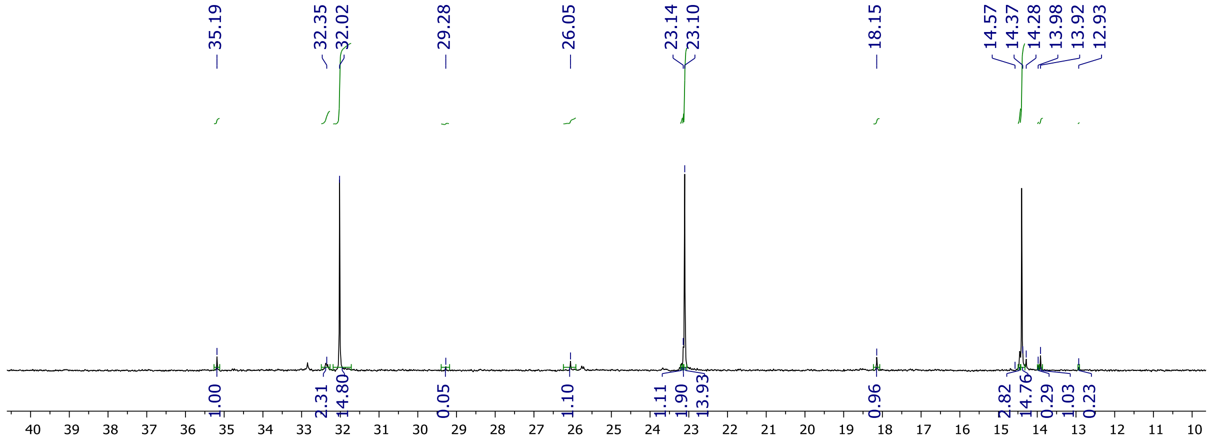

Trans-3-Hexene

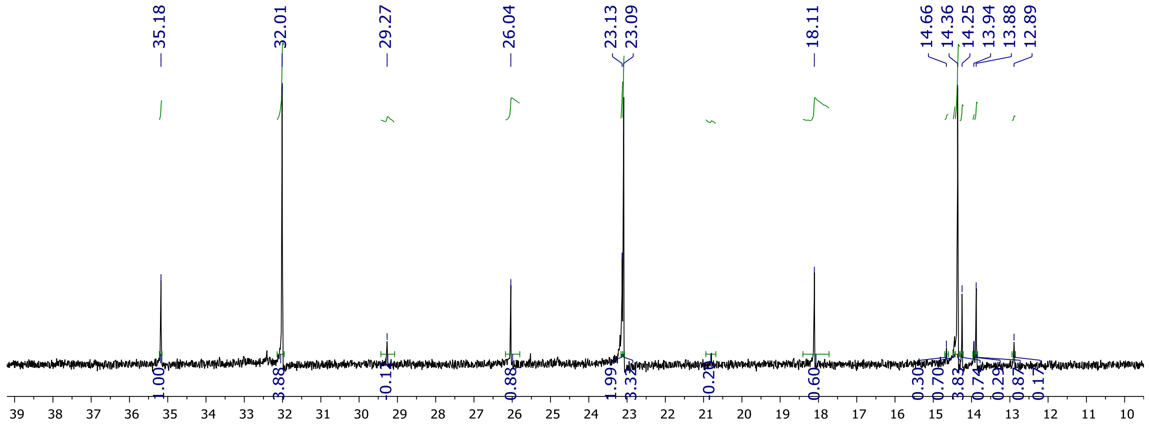

Cis-3-Hexene

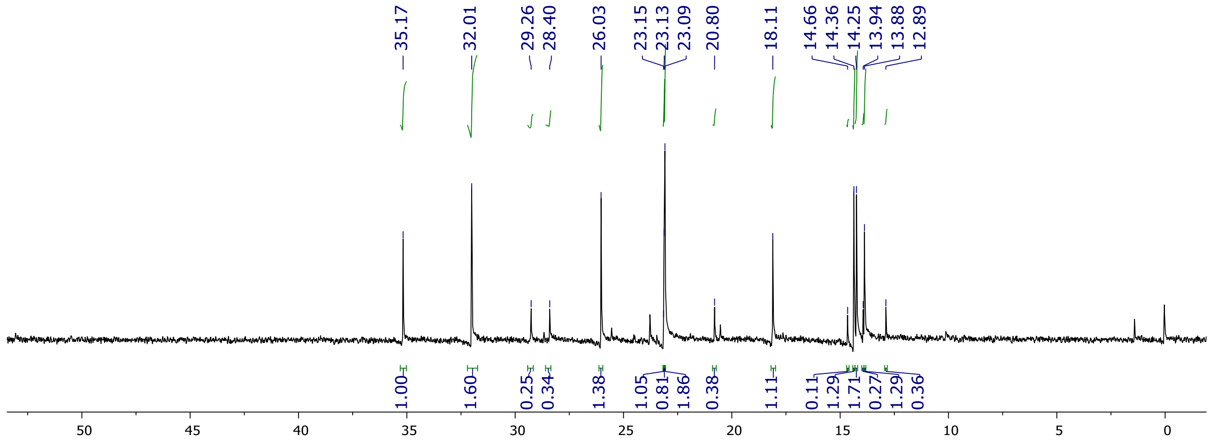

10.6 Reactions with (NACNAC)Fe-Toluene-μ6

(NACNAC)Fe-Toluene-μ6

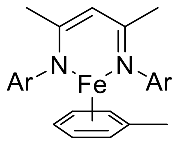

^{1}H

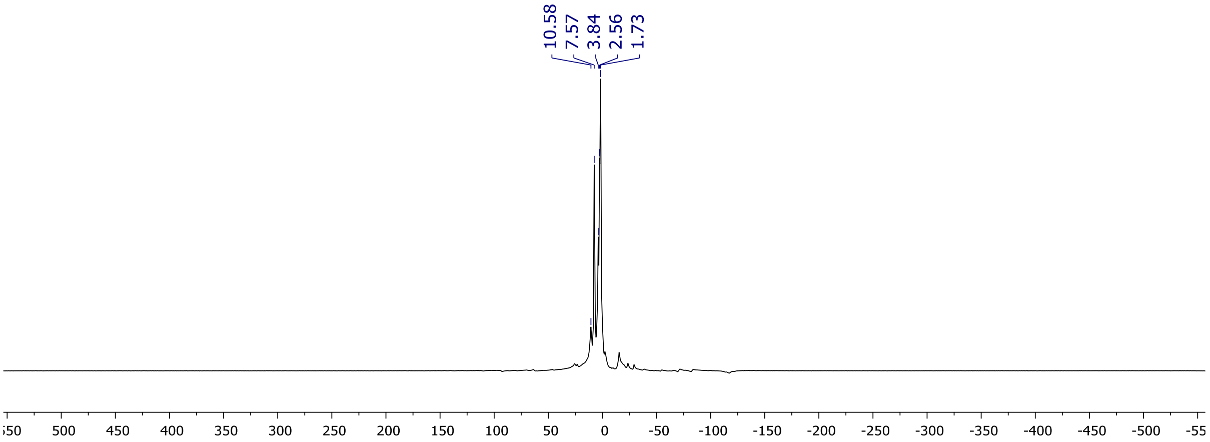

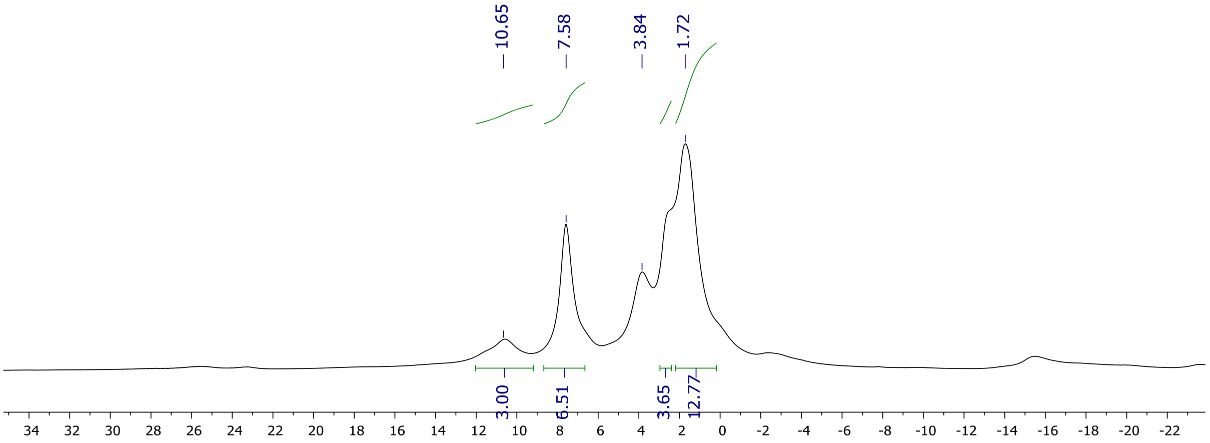

Reaction after 2 h, 60 °C (500 MHz, $CDCl_3$)

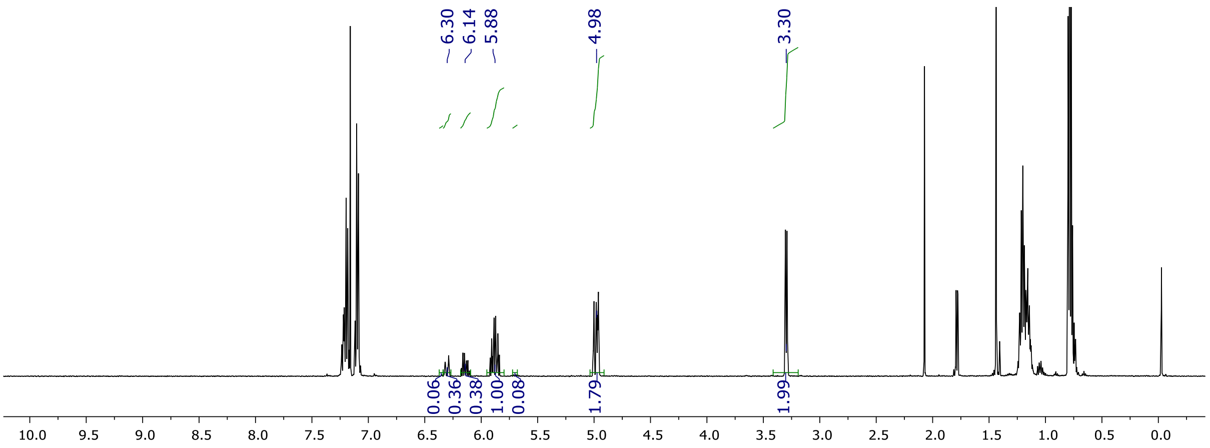

Reaction after 16 h, 60 °C (500 MHz, $CDCl_3$)

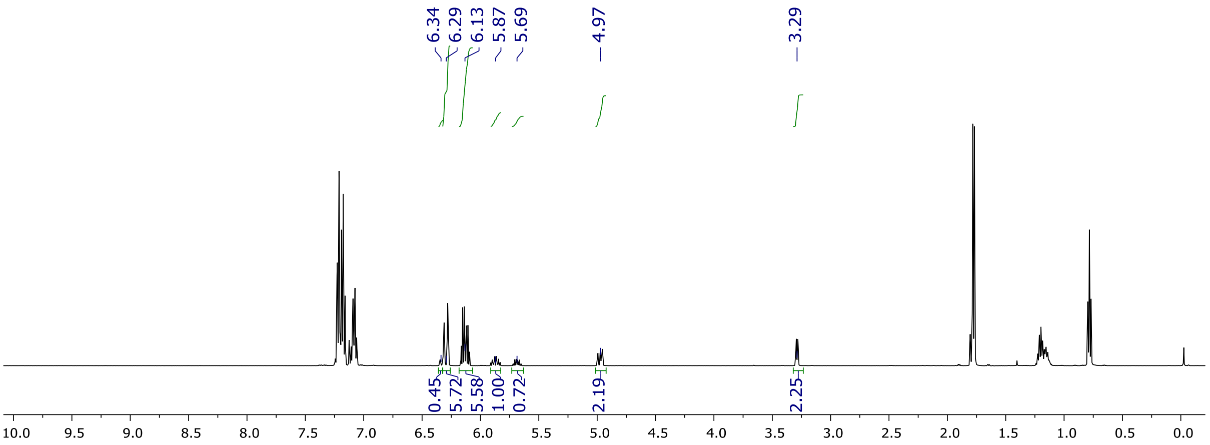

11. EPR Spectroscopy

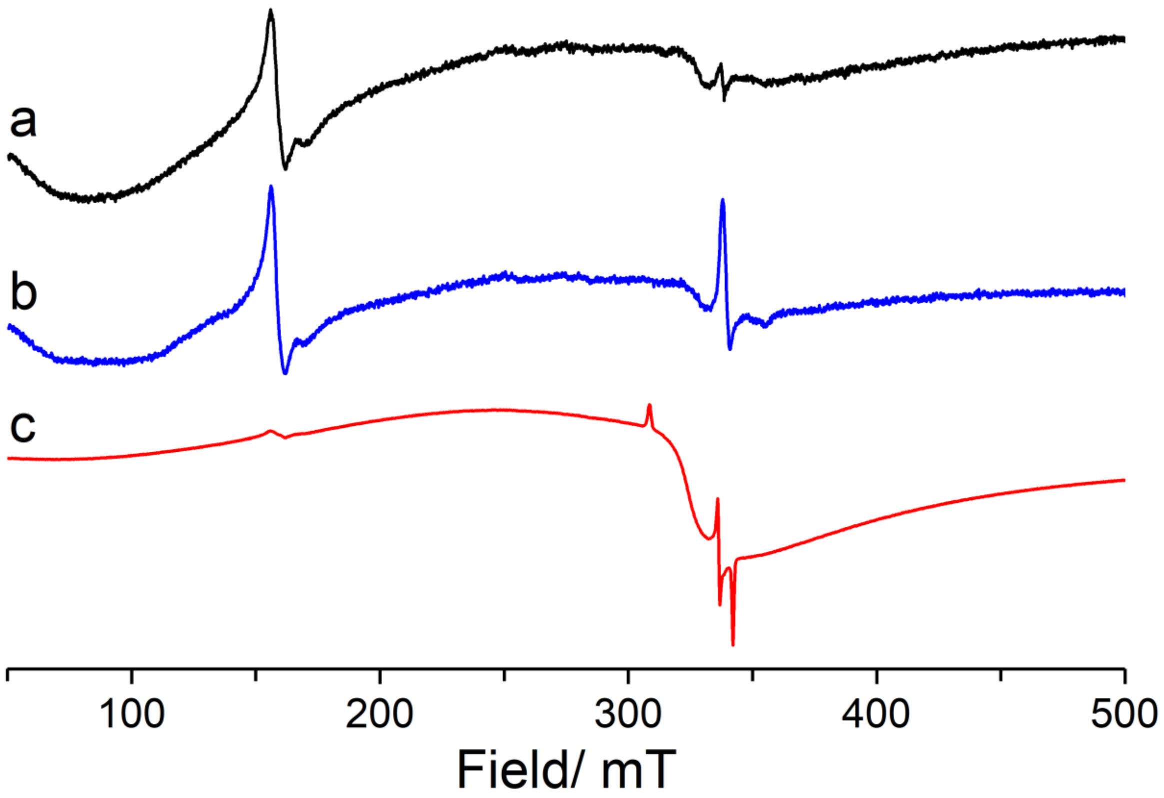

Fig S1 CW X-band EPR spectrum [T = 140 K] of (a) **1**, with (b) allylbenzene and (c) HBPin.

The frozen toluene EPR spectrum of **1** displays a low-intensity rhombic signal across a broad magnetic field range (**Fig S1a**). This is well reproduced by a simulation of an S = 5/2 high-spin state in the weak-field limit, in which the magnitude of the zero-field interaction between the unpaired electrons is larger than the available microwave quantum (i.e. D > 9.5 GHz = 0.31 cm^{-1}). The six-fold degeneracy from the five unpaired electrons splits into three doubly degenerate Kramer's doublets, with allowed transitions only *between* the levels within each doublet. Hence, the Kramer's doublet system is described by an effective *S'* = ½ system, and is characterised by g_{eff} = [5.76, 4.34, 2.01].

Experimental parameters:
Temperature = 140 K; MW power = 10 mW; Receiver gain = 3.56×10^4; modulation frequency = 100 kHz; modulation amplitude = 4.0 G.

Simulation parameters (performed using the Easyspin toolbox in Matlab): [18]
g_{iso} = [2]; gStrain = [0.007 0.005 0.005]; |E/D| = 0.07; DStrain = [3000]; lwpp = [3 1]

Upon addition of allylbenzene to 1, there is no change in the resulting EPR spectrum (**Fig S1b**). However, upon addition of HBpin to a benzene solution of 1, the appearance of a new

rhombic signal is observed in the EPR spectrum (**Fig S1c**), superimposed on a broad featureless signal (assigned to an Fe(III) centre). The rhombic signal is assigned to species $\mathbf{1_A}$, i.e. a low-spin Fe(I) d^7 centre, displayed in **Fig S2** focussing on the centre-field region. The spin Hamiltonian parameters for this species were extracted from simulation and are listed in Table S1, along with the calculated values for $\mathbf{1_B}$ and $\mathbf{1_D}$. All species are predicted to display a rhombic g profile, with one component (g_3) slightly lower than the free spin value of g_e (2.0023). This corresponds to a considerable $3d_{z^2}$ character (with some admixture from $3d_{x^2-y^2}$) in the SOMO, in agreement with the Loedwin reduced orbital MO population analysis calculated using the ORCA SCF-MO package from the geometry optimised structure (see **Fig S3**). The overall spin density is predominantly localised on the iron metal centre, with very little delocalisation onto the NacNac backbone, explaining the lack of observation of any ligand superhyperfine coupling (iron is only 2 % nuclear spin active, $I(^{57}Fe) = ½$, explaining the lack of metal hyperfine; see **Fig S3**). The relatively small Δg shifts from g_e observed for the $g_{1,2}$ parameters result from the small spin-orbit coupling constant for Fe (ζ_{Fe+} = 255 cm^{-1}). [19]

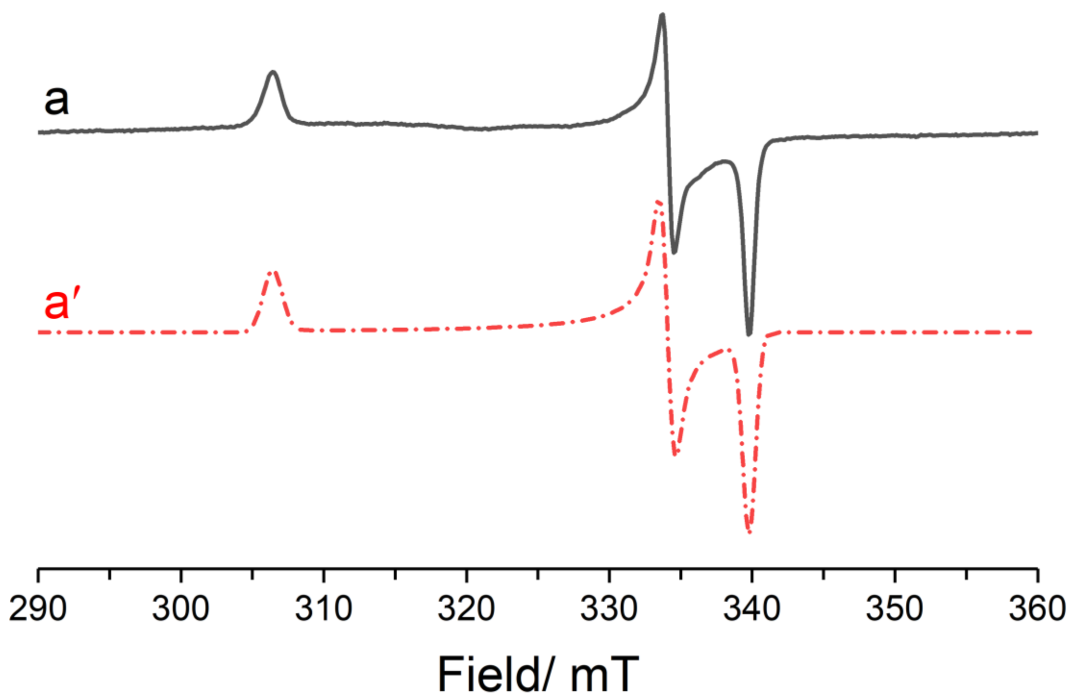

Fig S2 (a) Experimental, and (a’) simulation of CW X-band EPR spectrum [T = 140 K] of **1** + HBPin, focussing on the centre-field region. The broad signal originating from high-spin Fe(III) (seen in Figure 1 of the main text, and also in Fig S1) has been background subtracted.

Experimental parameters:
Temperature = 140 K; MW power = 10 mW; Receiver gain = 3.56 × 10^4; modulation frequency = 100 kHz; modulation amplitude = 4.0 G.

Simulation parameters (performed using the Easyspin toolbox in Matlab)[15]*:*
See Table S1; gStrain = [0.007 0.005 0.005]

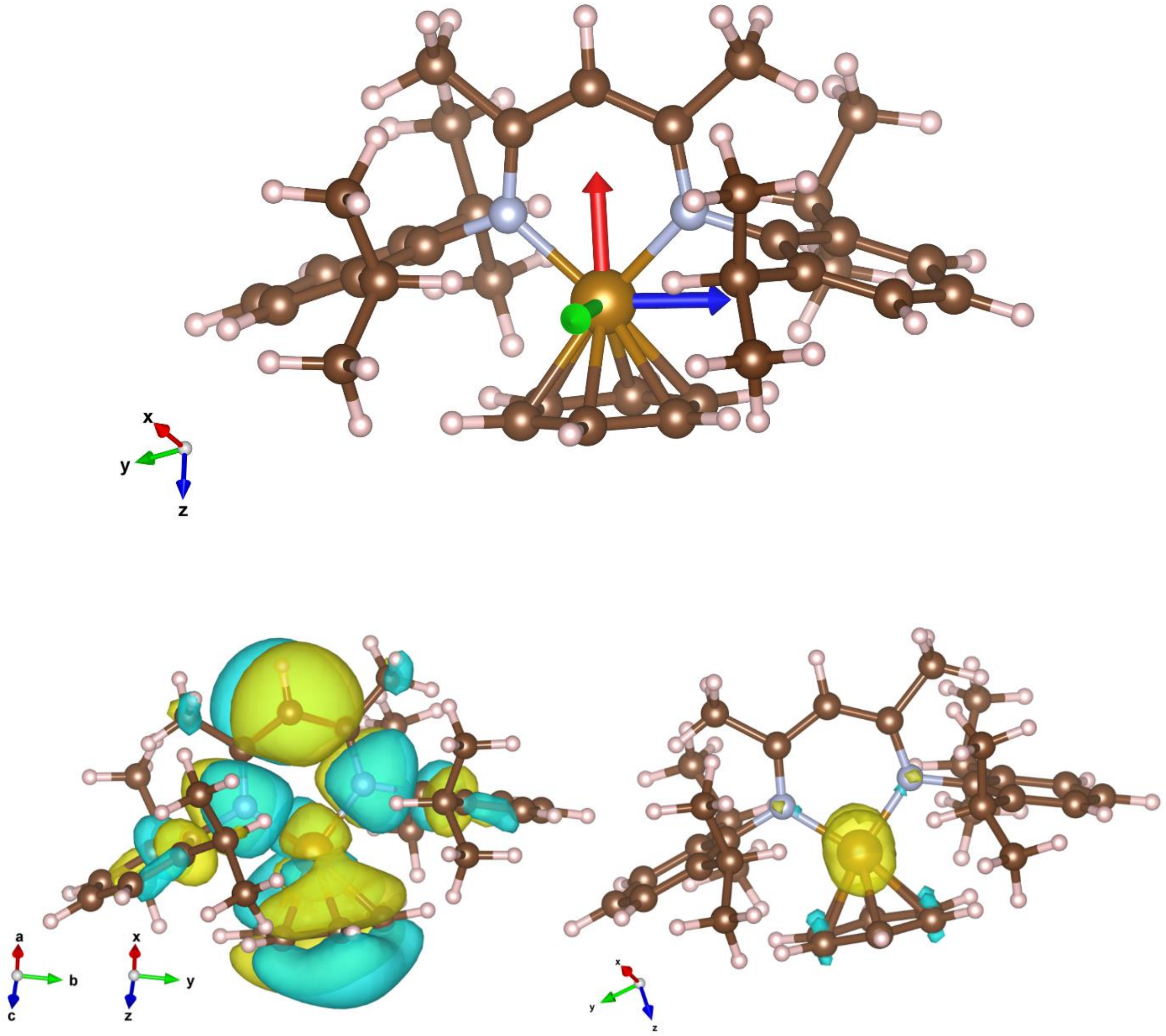

Fig S3 The orientation of the g-frame (arrows superimposed on structure) is plotted in the molecular frame (legend coordinate axis) for **1_A** (top). Spin density contour plots (right) and representation of the SOMO (left) for **1_A**.

Table S1 Spin Hamiltonian parameters of Fe(I) species (low spin, d^7) formed during the catalytic cycle (see Scheme 2 main text for labelling).

	**g-values**				**Euler angles/ rad**		
	g_1	g_2	g_3	g_{iso}	α	β	γ
**1 + HBPin**							
**Expt**	1.984	2.018	2.200	2.067	-1.900	1.490	0.669
**Species 1_A**							
**DFT**	1.979	2.015	2.128	2.041	-1.900	1.490	0.669

**Species 1_B**							
**DFT**	2.010	2.089	2.254	2.118	1.843	1.937	3.038
**Species 1_D_cis**							
**DFT**	1.979	2.094	2.221	2.098	1.793	1.480	-3.095

Table S2 Anisotropy and rhombicity parameters for the doublet Fe(I) species generated during the catalytic cycle.

	**g-values**				**Anisotropy parameters**			
	g_1	g_2	g_3	g_{iso}	$\Delta g_3 - g_1$	Δg_3-g_2	Δg_2-g_1	Δg_{rel}
**1 + HBPin**								
**Expt**	1.984	2.018	2.200	2.067	0.216	0.182	0.034	84.3
**Species 1_A**								
**DFT**	1.979	2.015	2.128	2.041	0.149	0.113	0.036	75.8
**Species 1_B**								
**DFT**	2.010	2.089	2.254	2.118	0.244	0.165	0.079	67.6
**Species 1_D_cis**								
**DFT**	1.979	2.094	2.221	2.098	0.242	0.127	0.115	52.5

$\Delta g_{rel} = (\Delta g_3 - g_2 / \Delta g_3 - g_1) \times 100$

EPR Spectroscopy Experimental:

Samples for EPR measurements were prepared under an N_2 atmosphere in a glovebox. A solution of complex **1** was prepared by dissolving ca. 4 mg in 200 µL of dry benzene (in all cases, a further 10 µL of dry toluene was also added to improve the quality of the polycrystalline glass formed in frozen solution, and thereby enhance the quality of the EPR spectra). The solutions were transferred to a Young's EPR tube (Wilmad Labglass 727-LPV-250M) in the glove box and then cooled to 77 K before rapid transfer to the pre-cooled EPR cavity. The X-band CW EPR measurements were performed on a Bruker EMX spectrometer utilizing an ER4119HS resonator, 100 kHz field modulation at 140 K.

EPR Density Functional Theory

All density functional theory (DFT) calculations were carried out with ORCA (version 4.1.0). [20] A combination of basis sets was used for calculation of the spin Hamiltonian EPR parameters, including CP for Fe, and def2-TZVP on C/ H/ N atoms, in conjunction with the B3LYP density functional and RIJCOSX approximation. [21] The grid sizes were set to Grid5 and "normal" SCF criteria (with Slow Convergence, maximum of 500 iterations). The spin–orbit mean field

operator (SOMF(1X)) was used, and the origin for the g-tensor was taken at the centre of the electronic charge.

12. UV-Vis Spectroscopy

Experiments were carried out on a Cary 60 UV-Vis Spectrometer at approximately 1 $mmoldm^{-3}$ in dry benzene solvent. A bespoke cuvette with a J-Young tap adaptor was used to perform measurements in. Samples were prepared under an argon atmosphere – in cases where catalytic solutions were utilised, an aliquot of catalytic mixture was diluted for analysis. A solution of benzene was used as a zero reference.

[Fe]-CH2TMS λ_{max} =498 nm

[Fe]-CH2TMS λ_{max} =490 nm

Catalytic Mixture with $NH_3 \cdot BH_3$ λ_{max} = 494 nm, 552 nm

Catalytic Mixture with HBpin λ_{max} = 497 nm, 547 nm

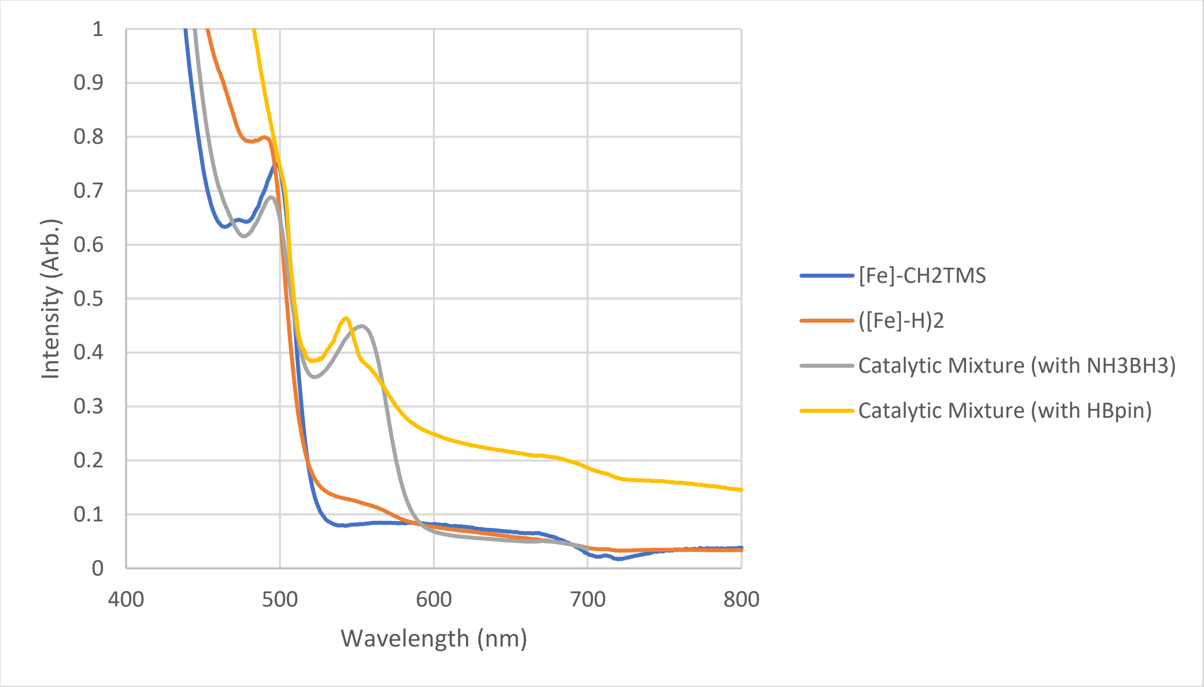

13. Further Studies into Catalyst Activation and Active Species

We have previously reported that combination of **1** and HBpin leads to iron hydride complex **4** forming [2], as observable by both ^{1}H and ^{11}B NMR (standard reaction setup without substrate added). As we have discussed elsewhere, **4** is inactive in catalysis and given the presence of additional oxidation states present in the reaction we believe a further reaction of HBpin and **4** occurs. **4** has previously been shown to react with BMe_3 and B^tBu_3 under similar conditions, [22] and we postulate that HBpin reacts with and assists in a disproportionation of **4** into Fe(I) and Fe(III) species (Scheme 13.1). This is supported by the

observation that 2 equivalents of HBpin relative to **1** are needed to achieve high conversions in catalysis.

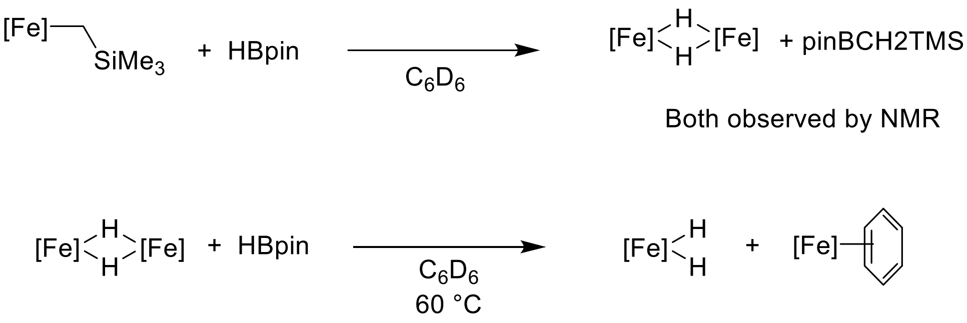

Scheme 13.1 Proposed catalytic activation with HBpin

Reactions with **1** and $H_3N{\cdot}BH_3$ are considerably more complex and lead to a range of signals produced, including but by no means exclusively **4**. We can isolate bridging iron amine-borane complexes crystallographically that are catalytically inactive, which we postulate arise from further reactions of **4** with dehydrocoupled ammonia borane, and account for Fe(III) signals observable in EPR.

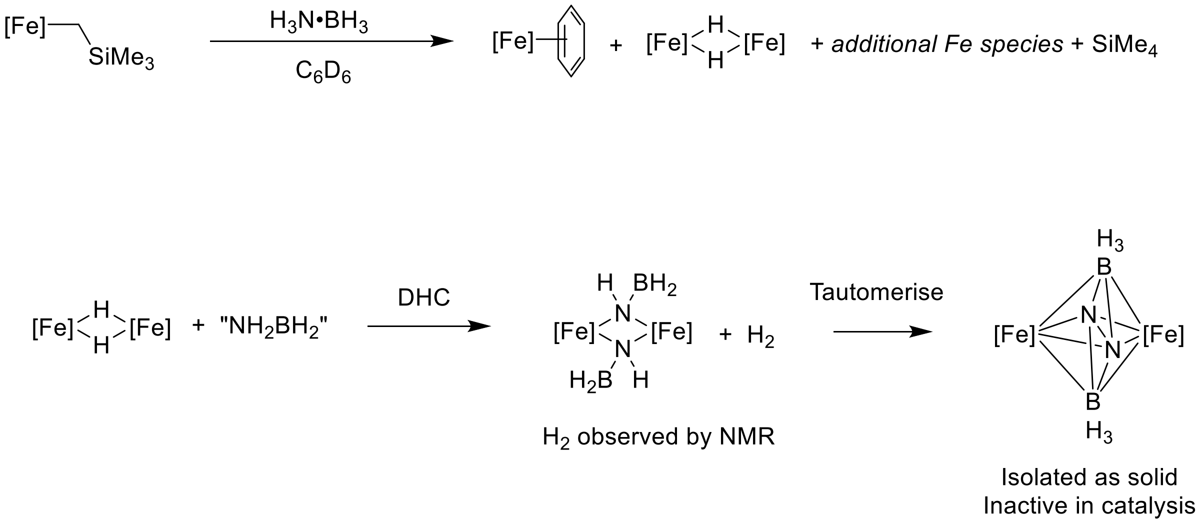

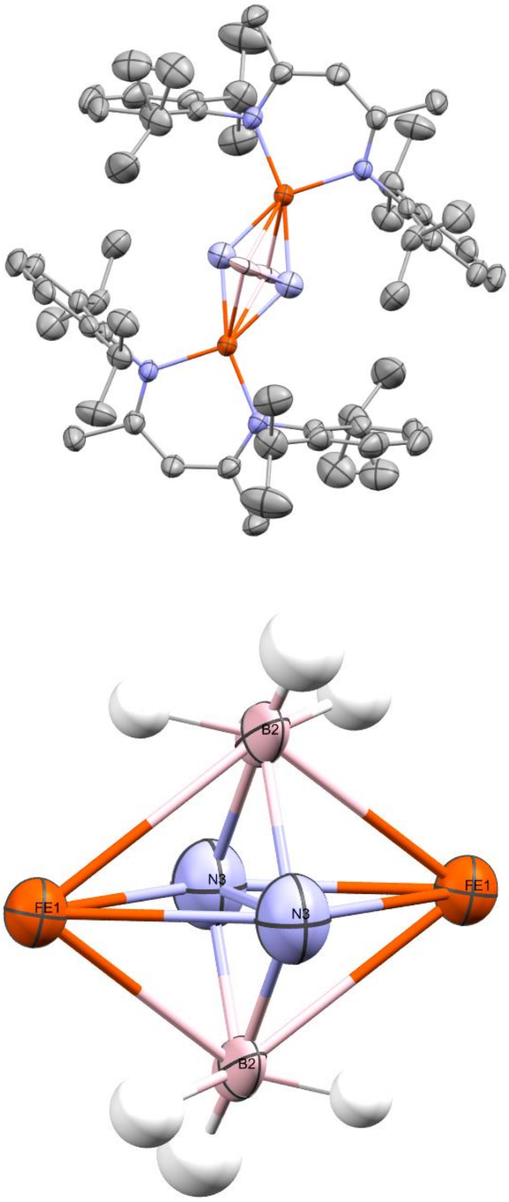

Scheme 13.2 Proposed catalytic activation with ammonia borane, and proposed route of formation for bridging iron amine-borane species. Crystal structure depicted at 50% probability, but complete confidence in nitrogen vs. boron assignment has not be possible therefore final cif file is not provided and structure is not deposited in the CSD. Bond lengths (Å) Fe1-B2 2.421; B2-N3 1.585; N3-N3 1.768; Fe1-N3 2.080.

Wide sweep width NMR experiments were undertaken under catalytic conditions to determine if any common species were present in solution with both HBpin and $NH_3{\cdot}BH_3$, or if key species such as $\mathbf{1_A}$ or $\mathbf{1_B}$ could be determined using NMR spectroscopy. As stated above, the spectrum of a stoichiometric reaction of $H_3N.BH_3$ and **1** in C_6D_6, a complex mixture is formed. However, common species are also observed in the ^{1}H NMR spectrum of the catalytic reaction (**1** (5 mol%) + $H_3N.BH_3$ (10 mol%) + allylbenzene (0.5 mmol) + C_6D_6 (0.6 mL); see stacked spectrum (Figure S4.4). Comparing the two catalytic reactions using HBpin or $H_3N{\cdot}BH_3$ as the reductant, there are common peaks also observed. Rather than attribute these

common iron species as active intermediates in catalysis, we attribute these to off-cycle species.

Fig S4.1 Wide sweep ^{1}H NMR of reaction of $H_3N{\cdot}BH_3$ and **1** (500 MHz, C_6D_6)

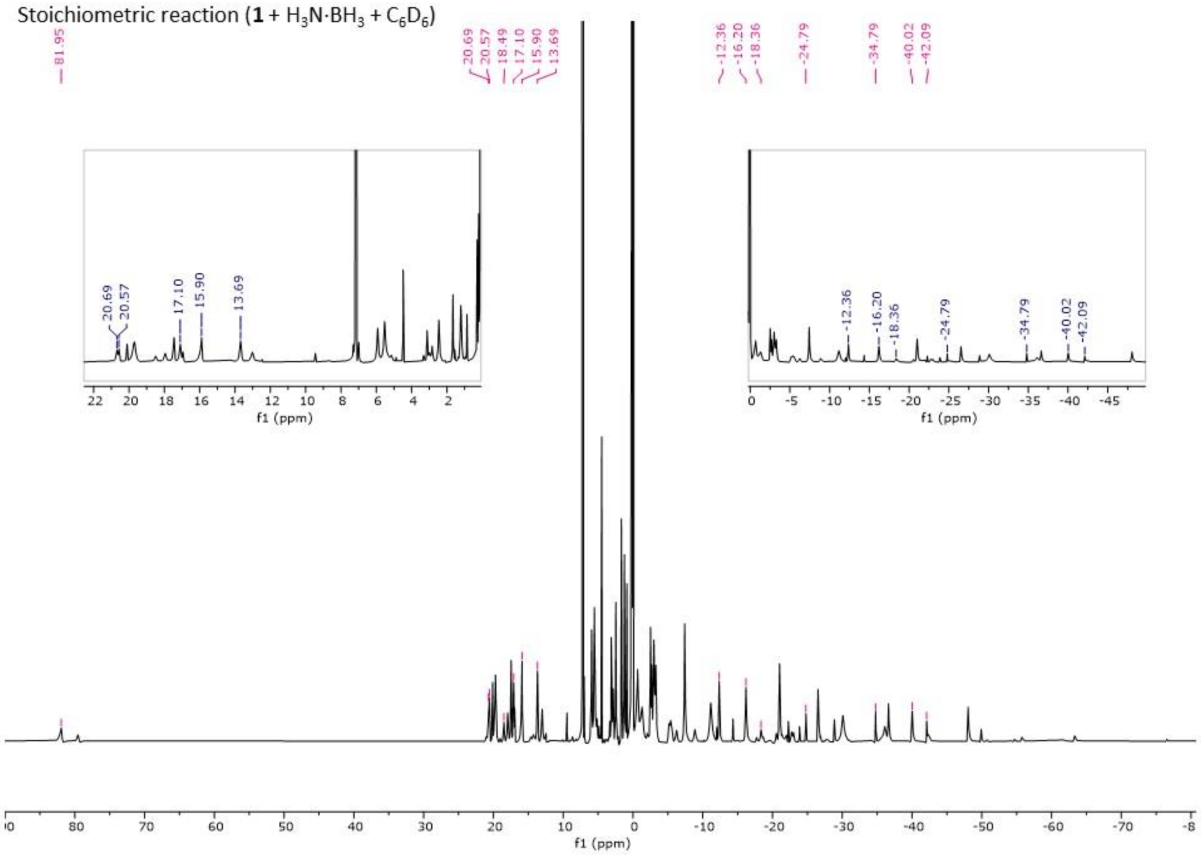

Fig S4.2 Wide sweep ^{1}H NMR of standard reaction conditions using HBpin (500 MHz, C_6D_6)

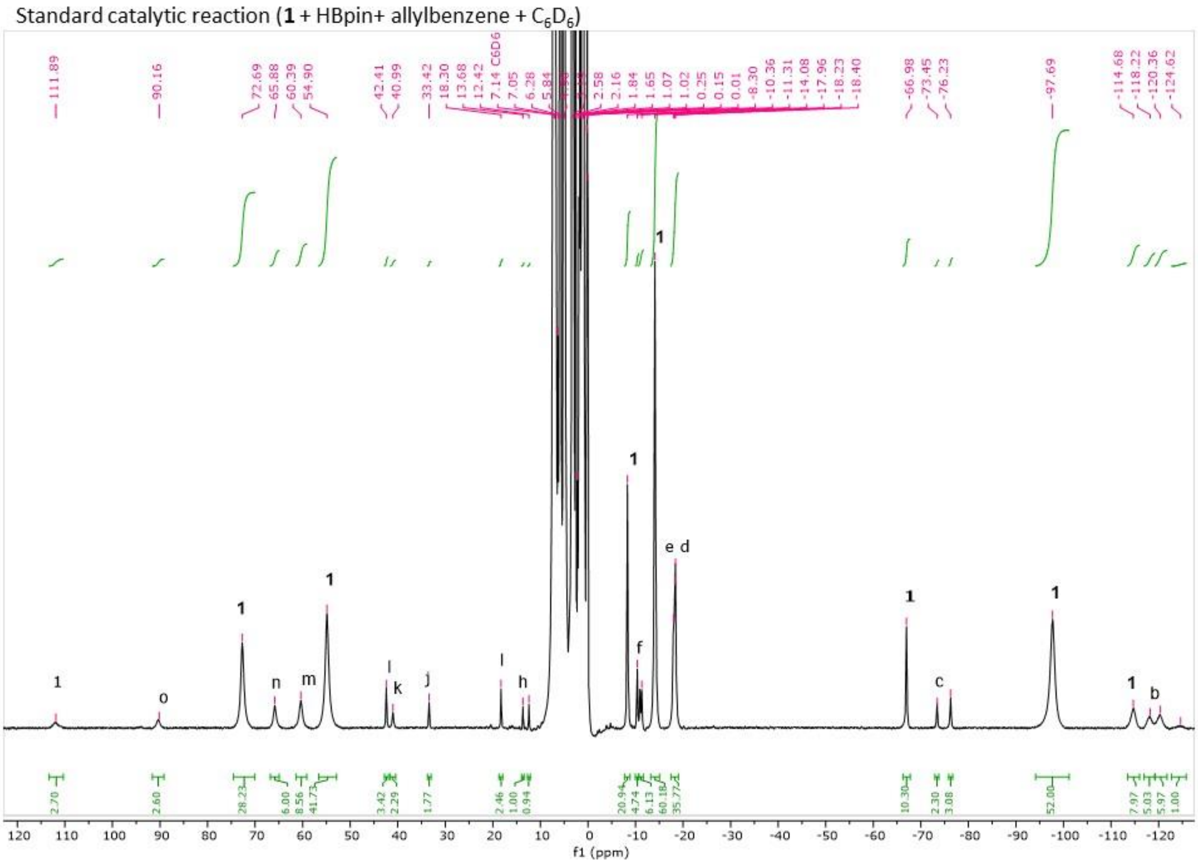

Fig S4.3 Wide sweep ^{1}H NMR of standard reaction conditions using $H_3N{\cdot}BH_3$ (500 MHz, C_6D_6)

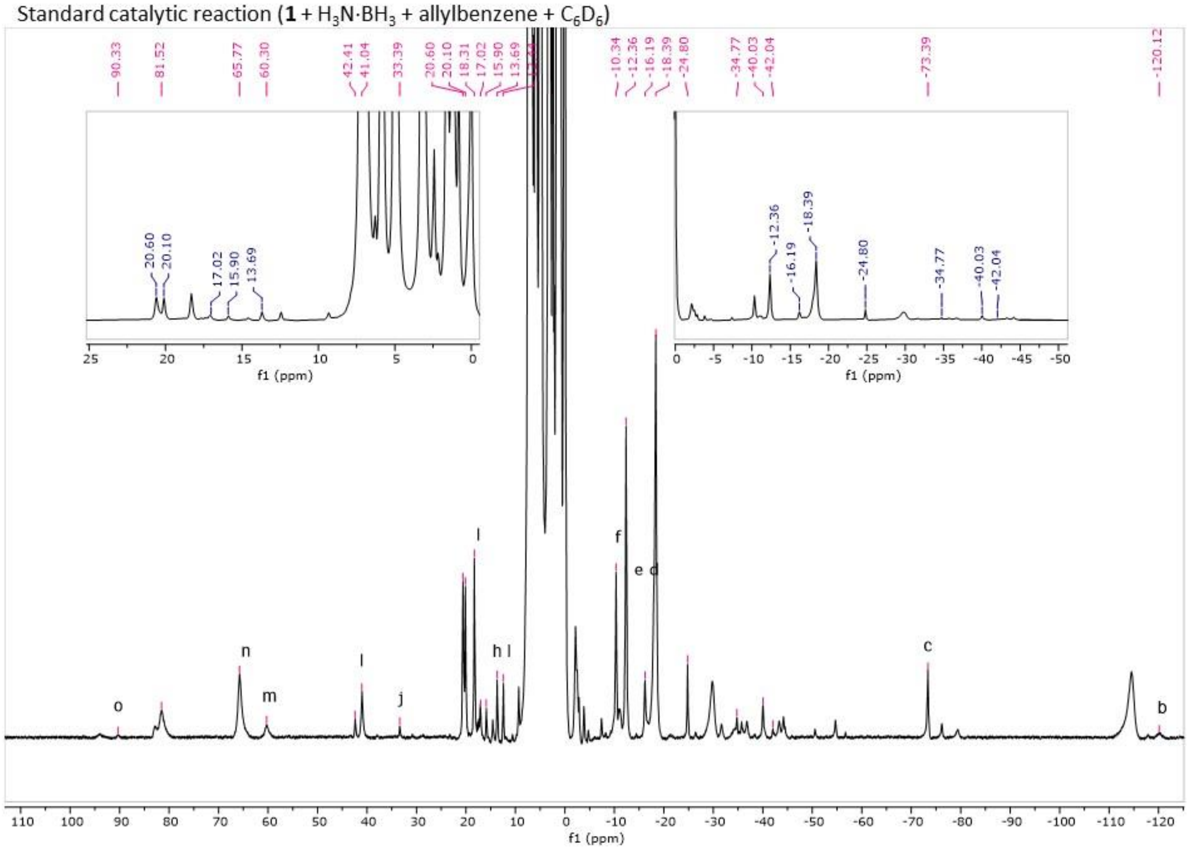

Fig S4.4 Overlaid wide sweep 1H NMR spectra indicating common peaks

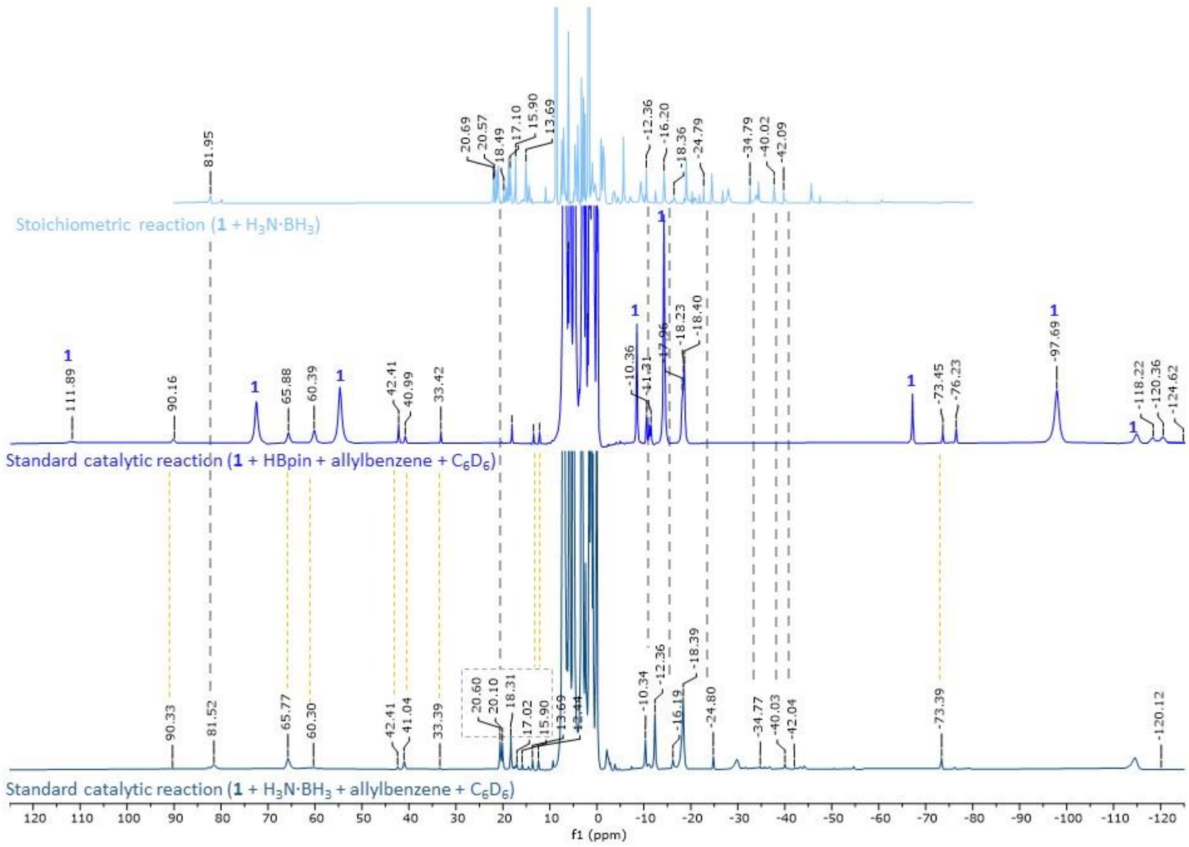

14. Computational Studies

Computational Details:

Fe(I/III) Mechanism:

Calculations of the Fe(I/III) catalytic cycle were carried out using the Jaguar software package using the B3LYP density functional with Grimme's D3 dispersion correction, with frequency calculations carried out at 298K.[21, 23] Fe was modelled using the triple-zeta Los Alamos effective core potential as implemented in Jaguar, with all other atoms modelled using the 6-31G* basis set. Implicit solvation was accounted for using a polarisable continuum model with benzene as the solvent. [24]

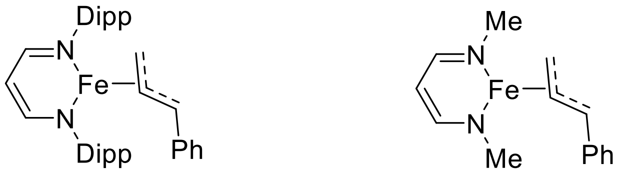

$\mathbf{1}_{C\_trans}$ $\mathbf{1}_{C\_trans\_Me}$

Fig S5 Full (left) and simplified (right) $\mathbf{1}_{c\_trans}$ species for which MECP calculations were attempted

We attempted to calculate minimum energy crossing points (MECPs) for the Fe(I/III) cycle, which were calculated using Gaussian 16[25] at the B3LYP/6-31G* or B3LYP/LANL2DZ theory level with the MECP optimisation software developed by Harvey *et al.*[26] Convergence for the full system was unsuccessful, so we attempted to locate crossing points using a simplified ligand system (NacNac Dipp groups replaced by Me, see above, Fig. S5) for the crossing between the doublet and sextet Fe(III) surface for a geometry slightly post the doublet 1_B-1_C transition state, with a crossing for a sextet to doublet pre-TS 1_c intermediate also attempted. Unfortunately, these failed to complete as well.

<u>Fe(II) Mechanism:</u>

Computational studies of the Fe(II) catalytic cycle were carried out with a computational approach similarly to the Fe(I/III) mechanism described above, although Grimme's D3 dispersion correction was not used and frequency calculations were carried out at 333.15K for the Fe(II) cycle to simulate experimental conditions and explore the temperature effect on the thermodynamic well for species B.

Fe (I/III) Mechanism:

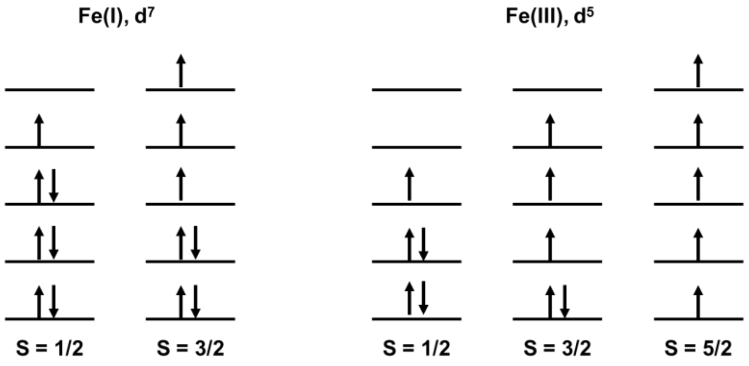

Figure S6 possible spin-states for d^7 and d^5 metal complexes, with the multiplicity of a given state described by 2S+1.

All energies are reported in kcal mol^{-1}, relative to the doublet ground state species $\mathbf{1}_{B\_neutral}$. Potential and free energies in Hartrees/a.u for structures can be found in the xyz files of the ESI.

The calculated stability of the quartet electronic configuration throughout the catalytic cycle would initially indicate that this is the active catalyst species, however, the activation barrier to oxidative addition from the initial on-cycle species $\mathbf{1}_{B\_trans}$ in this spin state is too large (45.0 kcal mol^{-1}) to be accessible under the experimental conditions. Additionally, the EPR studies completed clearly indicate the presence of an Fe (I) d^7 low-spin species.

Spin Crossover Evidence:

The available experimental data suggest that the likely active species for this reaction is predominantly the doublet for Fe(I) and Fe(III). Our computational investigation suggests that the reaction could proceed either solely on the doublet surface, or involve surface crossover with the sextet surface in the region of the intermediate trans-allyl complex ($\mathbf{1}_{C\_trans}$). We have attempted to locate minimum-energy crossing points (MECPs) for this part of the catalytic cycle, but these calculations failed to converge from several guess geometries after 100 steps. The EPR results indicating the presence of both low-spin Fe(I) and high-spin Fe(III) species suggest that such a crossover could occur, and this is supported by only small calculated energy differences between the two electronic configurations. Both the doublet and sextet surfaces calculated support the experimentally observed *trans*-selectivity for the isomerisation of allylbenzene into *trans*-β-methylstyrene.

To further support this, we calculated vertical excitation energies (calculation of the excitation of an electron from one electronic configuration to another without allowing for any structural rearrangement) using the optimised geometries of the $\mathbf{1}_{B\_C}$, $\mathbf{1}_{C}$ and $\mathbf{1}_{C\_D}$ species for both the *cis* and *trans* pathways in the doublet and sextet multiplicities, and the results of these calculations strengthen our hypothesis that any spin-crossover event is likely to occur close to the $\mathbf{1}_{C\_trans}$ geometry, particularly for the *trans* selective pathway. The calculated VEE's for both the doublet → sextet and sextet → doublet geometries of $\mathbf{1}_{C\_trans}$ are very small (< 0.1 kcal mol^{-1}), and these structures are very similar structurally.

Table S3 Vertical excitation energies between doublet and sextet spin configurations for Fe(III) species.

Doublet Geometries

**Doublet**	**Doublet (Hartrees)**	**Sextet (Hartrees)**	**Vertical Excitation Energy[1] (kcal mol^{-1})**
$\mathbf{1}_{B\_C\_trans}$	-1711.818856	-1711.740846	48.95
$\mathbf{1}_{C\_trans}$	-1711.832469	-1711.83242	0.03
$\mathbf{1}_{C\_D\_trans}$	-1711.826656	-1711.78593	25.56
$\mathbf{1}_{B\_C\_cis}$	-1711.811178	-1711.741678	43.61

[1] Vertical Excitation Energy = ΔE for sextet spin configuration at the optimised geometry for the doublet spin configuration and *vice versa* for sextet optimised geometry.

**1**$_{C\_cis}$	-1711.829418	-1711.77108	36.61
**1**$_{C\_D\_cis}$	-1711.81716	-1711.759504	36.18

Sextet Geometries

Sextet	Sextet (Hartrees)	Doublet (Hartrees)	Vertical Excitation Energy (kcal mol^{-1})
**1**$_{B\_C\_trans}$	-1711.795903	-1711.81526	-12.15
**1**$_{C\_trans}$	-1711.828351	-1711.82832	0.02
**1**$_{C\_D\_trans}$	-1711.807268	-1711.813527	-3.93
**1**$_{C\_cis}$	-1711.833967	-1711.829265	2.95
**1**$_{C\_D\_cis}$	-1711.805309	-1711.81897	9.41

As no optimised geometry for the **1**$_{B\_C\_cis}$ transition state in the sextet spin-state could be located, no vertical transition energy to the doublet species were calculated.

Calculated Energies for the Fe (I/III) Catalytic Cycle:

The *cis* selective oxidative addition transition state (**1**$_{B\_C\_cis}$) could not be located for the quartet and sextet surfaces, so the energies of these were estimated by using single-point energy calculations from the optimised doublet **1**$_{B\_C\_cis}$ geometry. The transition states for the sextet surface were also calculated assuming that the d^5 configuration of the Fe(III) species is preserved, which is unlikely to be the case during an electron transfer process like oxidative addition or reduction elimination, so it is probable that these transition states are not true stationary points on the potential energy surfaces of other multiplicities.[2]

Table S4 Calculated free and potential energies for optimised species in the doublet spin configuration for the Fe(I/III) catalytic cycle.

Doublet (Spin = 1/2)

Species	ΔE	ΔE_{solv}	ΔG_{solv} (298.15K)
**1**$_{B\_neutral}$	0.00	0.00	0.00
**1**$_{B\_trans}$	-0.37	-0.06	0.32
**1**$_{B\_C\_trans}$	24.22	24.58	25.99

[2] These transition states are unlikely to be real and/or not be spin-contaminated by the doublet spin configuration, as the sextet spin configuration is not possible for d^7 species

**1**$_{C\_trans}$	15.60	16.03	18.33
**1**$_{C\_D\_trans}$	20.02	19.69	20.96
**1**$_{D\_trans}$	-5.88	-5.55	-4.63
**1**$_{B\_cis}$	-0.44	-0.74	1.39
**1**$_{B\_C\_cis}$	29.43	29.40	29.44
**1**$_{C\_cis}$	17.47	17.95	21.04
**1**$_{C\_D\_cis}$	25.22	25.64	25.84
**1**$_{D\_cis}$	-4.52	-4.39	-3.29

Table S5 Calculated free and potential energies for optimised species in the quartet spin configuration for the Fe(I/III) catalytic cycle.

Quartet (Spin = 3/2)

**Species**	**ΔE**	**ΔE$_{solv}$**	**ΔG$_{solv}$ (298.15K)**
**1**$_{B\_neutral}$	-20.37	-20.26	-20.37
**1**$_{B\_trans}$	-20.80	-20.63	-18.71
**1**$_{B\_C\_trans}$	29.71	28.96	26.33
**1**$_{C\_trans}$	4.16	4.46	3.04
**1**$_{C\_D\_trans}$	7.59	6.93	3.50
**1**$_{D\_trans}$	-26.77	-26.32	-16.46
**1**$_{B\_cis}$	-20.20	-19.71	-18.72
**1**$_{B\_C\_cis}$	**29.13**[3]	**29.70**[3]	-
**1**$_{C\_cis}$	19.03	19.00	19.27
**1**$_{C\_D\_cis}$	14.50	12.54	10.41
**1**$_{D\_cis}$	-23.20	-22.95	-1.95

[3] These values are an estimate calculated from single-point calculations of the doublet optimised **1**$_{B\_C\_trans}$ with the quartet or sextet spin configuration, as the quartet & sextet transition states could not be found.

Table S6 Calculated free and potential energies for optimised species in the sextet spin configuration for the Fe (I/III) catalytic cycle.

Species	Sextet (Spin = 5/2)		
	ΔE	ΔE_{solv}	ΔG_{solv} (298.15K)
$\mathbf{1}_{B\_C\_trans}$	39.25	38.98	34.05
$\mathbf{1}_{C\_trans}$	18.99	18.62	15.24
$\mathbf{1}_{C\_D\_trans}$	32.68	31.85	26.23
$\mathbf{1}_{B\_C\_cis}$	**69.80**[3]	**73.01**[3]	-
$\mathbf{1}_{C\_cis}$	15.84	15.10	11.21
$\mathbf{1}_{C\_D\_cis}$	32.94	33.08	28.53

As discussed in the main report, the calculated energy surfaces for the different spin configurations, as well as our experimental evidence, point to the reaction proceeding largely on the doublet surface for both Fe(I) and Fe(III) species. While the quartet spin configuration is calculated to be more stable, the activation of this species to undergo oxidative addition is not likely ($\Delta G^{\ddagger}$ = 45.0 kcal mol^{-1}) and EPR evidence suggests the intermediate spin quartet is not present under experimental conditions. The reaction is also unlikely to proceed mostly on the sextet surface, despite a method evaluation (discussed below) showing that it is likely that 1_C is possibly more stable in the sextet spin configuration. This is due to the larger activation barriers in the sextet spin configuration to reductive elimination compared to the doublet spin configuration (for the *trans* selective reductive elimination, $\Delta G^{\ddagger}_{sextet}$ = 11.0 kcal mol^{-1} vs $\Delta G^{\ddagger}_{doublet}$ =2.6 kcal mol^{-1}).

Computational Method Testing for Fe(I/III) catalytic cycle:

To further assess the likelihood of spin crossover events near $\mathbf{1}_{C\_trans}$ and for the reaction overall, a computational method evaluation was carried out. All energies shown in this method evaluation are single-point solvated potential energies (ΔE_{solv}) of the B3LYP-D3/6-31G* (LACV3P*) optimised geometries.

The method evaluation shows that while there are significant differences between the results obtained with each method, general trends remain relatively constant. The doublet spin configuration is calculated to have accessible barriers to oxidative addition, while the quartet, which is consistently calculated to be more stable, has activation barriers that make oxidative addition unlikely. While the sextet spin configuration is calculated to be higher in energy than the doublet, we accept that the relative stability of $\mathbf{1}_{C\_trans}$ for the doublet and sextet spin configurations tends to be small, although the EPR evidence still supports that our conclusion that spin-crossover is likely to occur.

Table S7 Single point solvated potential energies of B3LYP-D3/6-31G(d)/LACV3P* geometries using different functionals for the doublet (S = 1/2) spin configuration.

	ΔE$_{solv}$ (kcal mol^{-1})			
**Species**	**B3LYP-D3**	**BP86 [27]**	**PBE0 [28]**	**PBE0-D3**
**1**$_{B\_neutral}$	0.00	0.00	0.00	0.00
**1**$_{B\_trans}$	-0.06	2.41	0.53	0.18
**1**$_{B\_C\_trans}$	24.58	21.46	25.72	23.65
**1**$_{C\_trans}$	16.04	19.24	24.92	20.67
**1**$_{C\_D\_trans}$	19.69	20.31	25.15	21.96
**1**$_{D\_trans}$	-5.55	1.77	-0.16	-3.16
**1**$_{B\_cis}$	-0.74	4.91	1.60	-0.14
**1**$_{B\_C\_cis}$	29.40	23.47	30.14	29.25
**1**$_{C\_cis}$	17.95	17.58	22.76	19.80
**1**$_{C\_1Dcis\ (TS)}$	25.64	23.24	27.89	25.40
**1**$_{Dcis}$	-4.39	0.97	-0.71	-3.77

Table S8 Single point solvated potential energies of B3LYP-D3/6-31G(d)/LACV3P* geometries using different functionals for the quartet (S = 3/2) spin configuration.

	ΔE$_{solv}$ (kcal mol^{-1})			
**Species**	**B3LYP-D3**	**BP86**	**PBE0**	**PBE0-D3**
**1**$_{B\_neutral}$	-20.26	-11.73	-17.98	-17.27
**1**$_{B\_trans}$	-20.63	-11.47	-26.71	-24.88
**1**$_{B\_C\_trans}$	28.96	34.69	35.82	33.76
**1**$_{C\_trans}$	4.46	14.85	6.33	3.20
**1**$_{C\_D\_trans}$	6.93	16.40	14.25	11.07
**1**$_{D\_trans}$	-26.32	-11.22	-23.39	-26.39
**1**$_{B\_cis}$	-19.71	-5.95	-22.48	-23.46
**1**$_{B\_C\_cis}$[4]	-	-	-	-
**1**$_{C\_cis}$	19.00	30.66	33.46	30.51
**1**$_{C\_1Dcis\ (TS)}$	12.54	18.98	18.50	16.02
**1**$_{Dcis}$	-22.95	-10.31	-19.79	-22.46

Table S9 Single point solvated potential energies of B3LYP-D3/6-31G(d)/LACV3P* geometries using different functionals for the sextet (S = 5/2) spin configuration.

	ΔE$_{solv}$ (kcal mol^{-1})			
**Species**	**B3LYP-D3**	**BP86**	**PBE0**	**PBE0-D3**
**1**$_{B\_C\_trans}$	38.98	-10.00	65.04	62.97

[4] As single-point optimisations of the doublet optimised **1**$_{B\_C\_trans}$ geometry were used, these structures were not included in the method evaluation.

**1_{C_trans}**	18.62	38.22	19.72	16.40
**$1_{C\_D\_trans}$**	31.85	-26.19	41.52	38.34
**$1_{B\_C\_cis}$**	-	-	-	-
**1_{C_cis}**	15.10	-35.52	49.72	46.77
**$1_{C\_1Dcis\ (TS)}$**	33.08	-11.18	55.46	52.97

Beyond testing the density functional used, we also explored the effect of introducing a higher quality basis set, 6-311+G(d,p). Determining the relative stability of our intermediate species **1_{C_trans}** is important, particularly for the doublet and sextet surfaces, where our initial 6-31G* calculations suggest that the sextet is more stable, providing evidence to support the experimentally observed high-spin Fe(III) species. Single-point calculations show that this relative stability is sensitive to computational method, as sextet **1_{C_trans}** is calculated to be 2.1 kcal mol^{-1} less stable than the doublet using 6-311+G(d,p), whereas our 6-31G(d) calculations produce the same trend with the sextet 3.4 kcal mol^{-1} less stable than the doublet for ΔE, but this is reversed for the ΔG values, where the sextet 1_{C_trans} is 3.1 kcal mol^{-1} than the doublet.

Table S9 Single point potential energies calculated using the 6-311+G(d,p) basis set on geometries optimised using B3LYP-D3/6-31g(d)/LACV3P*.

	ΔE_{solv} (kcal mol^{-1})		
**Species**	**Doublet**	**Quartet**	**Sextet**
**$1_{B\_neutral}$**	0.00	-21.15	-
**1_{B_trans}**	0.54	-21.50	-
**$1_{B\_C\_trans}$**	24.35	27.76	54.35
**1_{C_trans}**	14.70	3.32	16.77
**$1_{C\_D\_trans}$**	18.12	5.76	37.60
**1_{D_trans}**	-2.42	-25.74	-
**1_{B_cis}**	0.27	-19.39	-
**$1_{B\_C\_cis}$**	28.67	-	-
**1_{C_cis}**	17.54	19.01	29.76
**$1_{C\_D\_cis}$**	24.44	11.09	51.68
**1_{D_cis}**	-2.03	-22.39	-

Fe (II) mechanism:

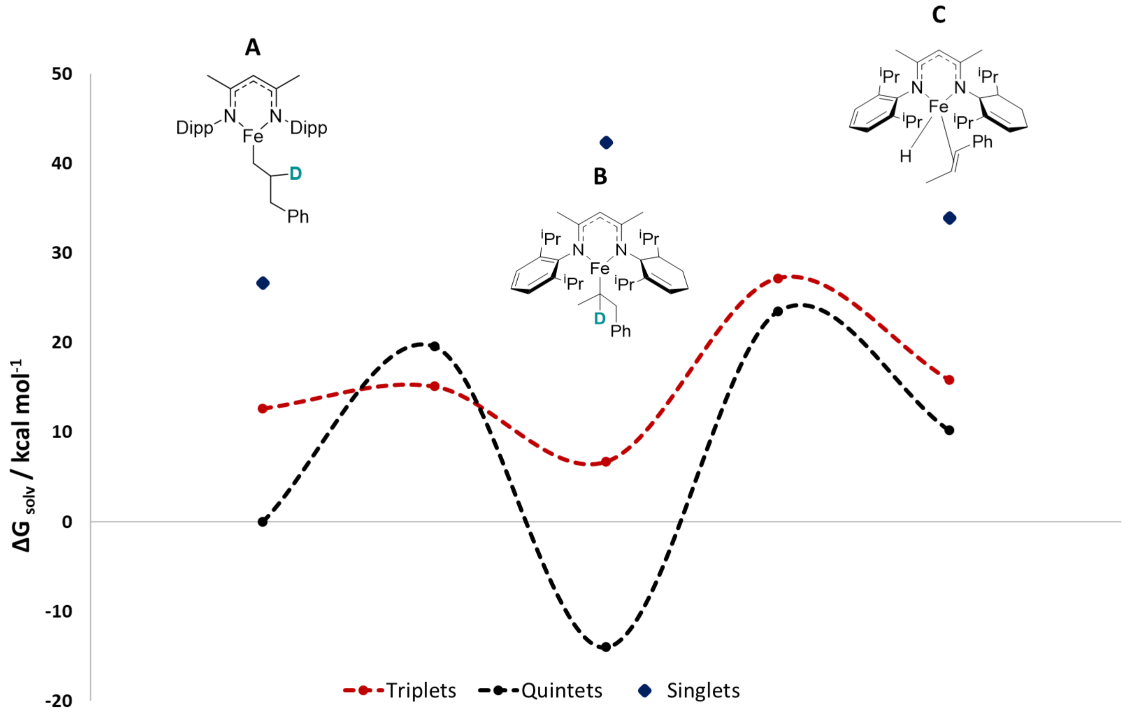

Figure S7: Calculated free energy surface for the discounted Fe(I) catalytic cycle for the isomerisation of allylbenzene to make trans-β-methylstyrene, showing the singlet, triplet, and quintet spin configurations.

The Fe(II) cycle shown above in **Figure S7** was discounted as the mechanism of catalysis due to experimental results indicating that no incorporation of D-labelled proton sources into the substrate was occurring during the reaction, and our computational investigation supports the exclusion of a redox-neutral cycle, as shown in the main report (**Scheme 2d**). The stability of species **B** in the high-spin quartet state forms a thermodynamic well which impedes the progress of the reaction ($\Delta G^{\ddagger}$ = 37.4 kcal mol^{-1} to the *trans*-selective β-hydride elimination). Additionally, the experimental EPR studies show that upon addition of HBPin or $H_3N.BH_3$ to **1**, the main signal observed is consistent with the formation of a Fe(I), d^7 low-spin species, as well as a broad signal which suggests the presence of an Fe(III) species. These results, as well as cyclic voltammetry studies which showed this reduction to Fe(I) from Fe(II) to be irreversible, suggest that the active catalyst species is not an Fe(II) species.

This was not expected, however, due to recently published work by the groups of Turculet, Ess and others, which computationally and experimentally explored an Fe(II) mechanism for alkene isomerisation-hydroboration, as well as a publication by Cundari and Holland exploring spin-crossover during β-H elimination in Fe (II) species.[29] However, our experimental and computational results indicate that such a mechanism is not likely for the reported catalytic activity to we observe.

All energies are reported in kcal mol^{-1}, relative to the quintet ground state species A.

Table S10 Calculated free and potential energies for optimised species in the singlet spin configuration for the Fe(II) catalytic cycle.

**Species**	**ΔE**	**ΔE_{solv}**	**ΔG_{solv} (333.15K)**
**A**	17.99	22.50	25.51
**A_B (TS)**	-	-	-
**B**	29.37	33.88	41.07
**B_C_trans (TS)**	-	-	-
**C**	22.15	26.67	32.55

Table S11 Calculated free and potential energies for optimised species in the triplet spin configuration for the Fe(II) catalytic cycle.

**Species**	**ΔE**	**ΔE_{solv}**	**ΔG_{solv} (333.15K)**
**A**	9.38	10.86	11.16
**A_B (TS)**	9.34	13.86	14.45
**B**	-0.77	0.67	5.28
**B_C_trans (TS)**	19.10	23.62	25.40
**C**	11.10	12.53	14.39

Table S12 Calculated free and potential energies for optimised species in the quintet spin configuration for the Fe(II) catalytic cycle.

**Species**	**ΔE**	**ΔE_{solv}**	**ΔG_{solv} (333.15K)**
**A**	0.00	0.00	0.00
**A_B (TS)**	17.57	18.50	19.59
**B**	-18.68	-17.42	-13.98
**B_C_trans (TS)**	21.82	22.48	23.45
**C**	3.95	1.82	2.44

15. References

[1] J. F. Sonnenberg, R. H. Morris, *Catal. Sci. Technol.* **2014**, *4*, 3426-3438.

[2] M. Espinal-Viguri, C. R. Woof, R. L. Webster, *Chemistry - A European Journal* **2016**, *22*, 11605-11608.

[3] K. A. K, B. Antoine, M. M. F, W. R. L, *Chemistry – A European Journal*, *21*, 15960-15963.

[4] D. Gasperini, A. King, N. T. Coles, M. F. Mahon, R. L. Webster, *ACS Catalysis* **2020**.

[5] F. Spitzer, C. Graßl, G. Balázs, E. M. Zolnhofer, K. Meyer, M. Scheer, *Angewandte Chemie International Edition* **2016**, *55*, 4340-4344.

[6] N. T. Coles, M. F. Mahon, R. L. Webster, *Organometallics* **2017**, *36*, 2262-2268.

[7] M. Espinal-Viguri, S. E. Neale, N. T. Coles, S. A. Macgregor, R. L. Webster, *Journal of the American Chemical Society* **2019**, *141*, 572-582.

[8] E. M. Leitao, I. Manners, *European Journal of Inorganic Chemistry* **2015**, *2015*, 2199-2205.

[9] Q. Wu, L. Wang, R. Jin, C. Kang, Z. Bian, Z. Du, X. Ma, H. Guo, L. Gao, *European Journal of Organic Chemistry*, *2016*, 5415-5422.

[10] M. J. Spallek, S. Stockinger, R. Goddard, O. Trapp, *Advanced Synthesis & Catalysis*, *354*, 1466-1480.

[11] M. Mayer, A. Welther, A. Jacobi von Wangelin, *ChemCatChem* **2011**, *3*, 1567-1571.

[12] N. Zhu, J. Zhao, H. Bao, *Chem. Sci.* **2017**, *8*, 2081-2085.

[13] H. Liu, M. Xu, C. Cai, J. Chen, Y. Gu, Y. Xia, *Organic Letters* **2020**, *22*, 1193-1198.

[14] X.-J. Wei, I. Abdiaj, C. Sambiagio, C. Li, E. Zysman-Colman, J. Alcázar, T. Noël, *Angewandte Chemie International Edition* **2019**, *58*, 13030-13034.

[15] A. M. del Hoyo, A. G. Herraiz, M. G. Suero, *Angewandte Chemie International Edition*, *56*, 1610-1613.

[16] J.-H. Jeon, J.-Y. Yang, N. Chung, H.-S. Lee, *Journal of Agricultural and Food Chemistry* **2012**, *60*, 12349-12354.

[17] G. A. Strohmeier, I. C. Eiteljörg, A. Schwarz, M. Winkler, *Chemistry – A European Journal* **2019**, *25*, 6119-6123.

[18] S. Stoll, A. Schweiger, *Journal of Magnetic Resonance* **2006**, *178*, 42-55.

[19] G. M. Cole, B. B. Garrett, *Inorg. Chem.* **1970**, *9*, 1898-1902.

[20] F. Neese, *WIREs Computational Molecular Science* **2018**, *8*, e1327.

[21] aS. H. Vosko, L. Wilk, M. Nusair, *Can. J. Phys.* **1980**, *58*, 1200-1211; bC. Lee, W. Yang, R. G. Parr, *Physical Review B* **1988**, *37*, 785-789; cP. J. Stephens, F. J. Devlin, C. F. Chabalowski, M. J. Frisch, *The Journal of Physical Chemistry* **1994**, *98*, 11623-11627; dA. D. Becke, *The Journal of Chemical Physics* **1993**, *98*, 5648-5652.

[22] Y. Yu, W. W. Brennessel, P. L. Holland, *Organometallics* **2007**, *26*, 3217-3226.

[23] a, Schrodinger. Inc, New York, NY, **2014**; bS. Grimme, J. Antony, S. Ehrlich, H. Krieg, *The Journal of Chemical Physics* **2010**, *132*, 154104; cL. Goerigk, S. Grimme, *PCCP* **2011**, *13*, 6670-6688.

[24] S. Miertuš, E. Scrocco, J. Tomasi, *Chem. Phys.* **1981**, *55*, 117-129.

[25] M. J. Frisch, G. W. Trucks, H. B. Schlegel, G. E. Scuseria, M. A. Robb, J. R. Cheeseman, G. Scalmani, V. Barone, G. A. Petersson, H. Nakatsuji, X. Li, M. Caricato, A. V. Marenich, J. Bloino, B. G. Janesko, R. Gomperts, B. Mennucci, H. P. Hratchian, J. V. Ortiz, A. F. Izmaylov, J. L. Sonnenberg, Williams, F. Ding, F. Lipparini, F. Egidi, J. Goings, B. Peng, A. Petrone, T. Henderson, D. Ranasinghe, V. G. Zakrzewski, J. Gao, N. Rega, G. Zheng, W. Liang, M. Hada, M. Ehara, K. Toyota, R. Fukuda, J. Hasegawa, M. Ishida, T. Nakajima, Y. Honda, O. Kitao, H. Nakai, T. Vreven, K. Throssell, J. A. Montgomery Jr., J. E. Peralta, F. Ogliaro, M. J. Bearpark, J. J. Heyd, E. N. Brothers, K. N. Kudin, V. N. Staroverov, T. A. Keith, R. Kobayashi, J. Normand, K. Raghavachari, A. P. Rendell, J. C. Burant, S. S. Iyengar, J. Tomasi, M. Cossi, J. M. Millam, M. Klene, C. Adamo, R. Cammi, J. W. Ochterski, R. L. Martin, K. Morokuma, O. Farkas, J. B. Foresman, D. J. Fox, Wallingford, CT, **2016**.

[26] J. N. Harvey, M. Aschi, H. Schwarz, W. Koch, *Theoretical Chemistry Accounts* **1998**, *99*, 95-99.

[27] aJ. P. Perdew, *Physical Review B* **1986**, *33*, 8822-8824; bA. D. Becke, *Physical Review A* **1988**, *38*, 3098-3100.

[28] C. Adamo, V. Barone, *The Journal of Chemical Physics* **1999**, *110*, 6158-6170.

[29] aC. M. Macaulay, S. J. Gustafson, J. T. Fuller, D.-H. Kwon, T. Ogawa, M. J. Ferguson, R. McDonald, M. D. Lumsden, S. M. Bischof, O. L. Sydora, D. H. Ess, M. Stradiotto, L. Turculet, *ACS Catalysis* **2018**, *8*, 9907-9925; bS. M. Bellows, T. R. Cundari, P. L. Holland, *Organometallics* **2013**, *32*, 4741-4751.
